# Multifocal fluorescence video-rate imaging of centimetre-wide arbitrarily shaped brain surfaces at micrometric resolution

**DOI:** 10.1038/s41551-023-01155-6

**Published:** 2023-12-06

**Authors:** Hao Xie, Xiaofei Han, Guihua Xiao, Hanyun Xu, Yuanlong Zhang, Guoxun Zhang, Qingwei Li, Jing He, Dan Zhu, Xinguang Yu, Qionghai Dai

**Affiliations:** 1 Department of Automation, Tsinghua University, Beijing, China; 2 Institute for Brain and Cognitive Sciences, Tsinghua University, Beijing, China; 3 Beijing National Research Center for Information Science and Technology, Tsinghua University, Beijing, China; 4 Department of Neurosurgery, The First Medical Center of Chinese PLA General Hospital, Beijing, China; 5 School of Medicine, Tsinghua University, Beijing, China; 6 Britton Chance Center for Biomedical Photonics - MoE Key Laboratory for Biomedical Photonics, Wuhan National Laboratory for Optoelectronics - Advanced Biomedical Imaging Facility, Huazhong University of Science and Technology, Wuhan, China; 7 IDG/McGovern Institute for Brain Research, Tsinghua University, Beijing, China

**Keywords:** Wide-field fluorescence microscopy, Fluorescence imaging

## Abstract

Fluorescence microscopy allows for the high-throughput imaging of cellular activity across brain areas in mammals. However, capturing rapid cellular dynamics across the curved cortical surface is challenging, owing to trade-offs in image resolution, speed, field of view and depth of field. Here we report a technique for wide-field fluorescence imaging that leverages selective illumination and the integration of focal areas at different depths via a spinning disc with varying thickness to enable video-rate imaging of previously reconstructed centimetre-scale arbitrarily shaped surfaces at micrometre-scale resolution and at a depth of field of millimetres. By implementing the technique in a microscope capable of acquiring images at 1.68 billion pixels per second and resolving 16.8 billion voxels per second, we recorded neural activities and the trajectories of neutrophils in real time on curved cortical surfaces in live mice. The technique can be integrated into many microscopes and macroscopes, in both reflective and fluorescence modes, for the study of multiscale cellular interactions on arbitrarily shaped surfaces.

## Abstract (web summary)

A wide-field fluorescence microscope leveraging a spinning disc and high-speed cameras enables the recording of neural activities and neutrophil trajectories at micrometric resolution on curved cortical surfaces in live mice.

## Main

A system-level study of neural interactions across multiple brain areas necessitates high-throughput recording of cellular activities with high spatial and temporal resolution. Recent progress in skull window techniques has facilitated direct optical access to extensive regions of the mouse cortex, achieved by employing curved glass^1^ or polymer optical windows^2^ or through the use of biocompatible reagents for cranium clearance^3–6^. However, a substantial challenge lies in imaging the cortex with cellular resolution on its intricate and non-planar surface, particularly when investigating widespread cellular correlations in functionally distinct cortical areas.

Mesoscopic cellular imaging of complex-shaped surfaces can be accomplished through either serial or parallel approaches. Serial approaches involve scanning the entire surface and achieving focus shift using fast focal adjustment or point spread function (PSF) engineering^7,8^. For example, the two-photon random access mesoscope^9^ allows fast 3D imaging of neural activity in arbitrary regions of interest across the entire imaging volume by efficient sampling, which has made an advance in serial approaches. Nonetheless, the throughput of serial approaches remains limited by factors such as raster scanning, laser repetition rate and detection bandwidth, typically reaching up to 100 million pixels per second. Parallel approaches, on the other hand, enable simultaneous recording of fluorescence intensity from multiple sources using charge-coupled device or complementary metal oxide semiconductor (CMOS) technology^1,10^ and allow for video-rate imaging with microscale resolution and a centimetre-scale field of view (FOV) by utilizing multiple cameras^11^. Spike-inference algorithms, such as the extended constrained non-negative matrix factorization (CNMF-E)^12^ and deep wide-field neuron finder (DeepWonder)^13^, have been employed to detect and extract cellular activities from wide-field images, enabling high-throughput analysis. However, achieving high-speed imaging across the entire complex-shaped surface remains a formidable challenge, primarily due to the difficulty in rapidly changing the focal plane throughout the entire FOV.

Several methods have been developed to increase the depth of field (DOF) in wide-field fluorescence microscopy. One common approach is fast axial scanning. Mechanical axial scanning with piezo stages is not able to handle the weight of the objective lens or specimen at high speed^14^. Remote focusing techniques, which use additional relay optics, are impractical for mesoscale wide-field detection because they result in more system complexity, decreased light transmission and higher cost^15^. Most electrically tunable lenses and the tunable acoustic gradient index of refraction lenses (TAG lenses) have trade-offs between the FOV, resolution and speed, making them unsuitable for mesoscale high-throughput imaging^7,15–17^. Although multi-actuator adaptive lens has been developed with several hundred-Hertz rates^18,19^, it has not yet been applied to mesoscale microscopy due to its limited aperture size. One remarkable advance in mesoscale imaging on complex-shaped surfaces is cortical observation by synchronous multifocal optical sampling microscopy, which has achieved high-speed near-cellular resolution imaging of the entire dorsal neocortical surface of an awake mouse, but cellular dynamics are not distinguishable due to its large pixel size^20^. The second approach elongates the PSF to increase the axial range: one straightforward way is to reduce the numerical aperture (NA) to increase DOF^21^, which results in lower resolution, lower signal-to-noise ratio and increased photobleaching and toxicity; spherical aberration^22^, diffraction-free beams such as Bessel beams^23,24^ and Airy beams^25,26^ and the aperture division method^27^ have also been used to increase DOF, but these methods also result in sidelobes, lower signal-to-noise ratio or reconstruction artefacts. The third approach involves computational methods such as light field microscopy^10,28–32^ and *z*-encoding methods^33,34^, which capture volumetric information simultaneously. These methods usually require additional prior information such as sparsity for high-resolution reconstruction, so they are prone to reconstruction artefacts. None of the above techniques has effectively targeted imaging to a non-planar surface at high speed under the constraint of a centimetre-scale FOV and micrometre-scale resolution.

In this Article, to address these challenges, we have developed a wide-field fluorescence microscope, spinning-disc multifocal fluorescence imaging of arbitrary surfaces (MFIAS), that enables imaging on non-planar surfaces while preserving high spatial resolution across a large FOV. We have developed an active imaging framework that includes automatic detection of the surface profile, active control of spatial–temporal coded illumination, high-speed spinning disc scanning and multiplexed detection. Leveraging the high optical throughput of the spinning disc and high-speed cameras (as in the real-time ultra-large-scale high-resolution macroscopy (RUSH) system^11^ and micro-camera array microscope^35^), MFIAS can achieve a scanning rate of 16.8 billion voxels per second, allowing for nearly continuous tuning of the focal depth over a maximum range of approximately 2 mm and video-rate acquisition. We have demonstrated the capability of MFIAS macroscopy by imaging neural activity and neutrophil migration across the intact superficial cortex of a mouse as well as other dynamic processes on complex surfaces. MFIAS is reliable for providing biological images without artefacts, flexible for diverse experimental systems and conditions and cost-effective in implementation, rendering it a valuable tool for biological research.

## Results

### MFIAS operating principle

**Fig. 1:**
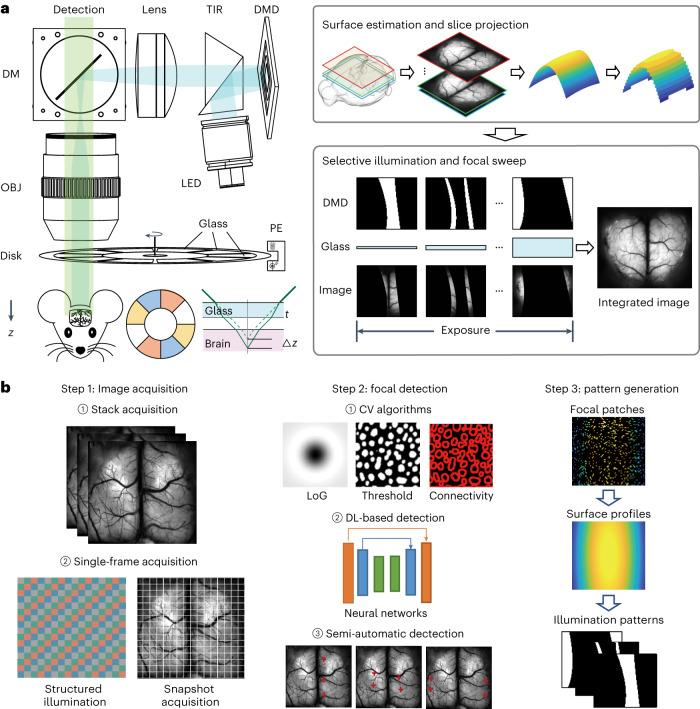
System schematic diagram. **a**, The MFIAS system comprises two components: selective illumination and focal modulation. The selective illumination component incorporates a DMD that is conjugated with the image plane and modulates the illumination patterns. The focal modulation component is a spinning disc with varying thicknesses of cover glass, which shifts the focal image to different depths. Before frame acquisition, a depth map is generated and converted into a series of illumination patterns displayed on the DMD. During each exposure period, the spinning disc completes one full rotation, while the DMD displays one pattern at each glass thickness. The resulting image is an integration of focal areas from different depths. TIR, total internal reflection prism; DM, dichroic mirror; OBJ, objective; LED, light-emitting diode; PE, photon emitter. **b**, Various techniques can be employed to generate illumination patterns. Image acquisition can be a stack for static or slow-varying surfaces or a single frame for fast-changing surfaces. The depth of each position can be determined using algorithms and/or refined through manual or semiautomatic detection algorithms. Red points in Step 2.3 are manually selected 3D coordinates. Finally, the depth map is generated, and the illumination pattern for each layer is calculated. CV, computer vision; DL, deep learning; LoG, Laplacian of Gaussian.

The MFIAS system, which integrates computer vision and optical design, has been developed to facilitate high-resolution dynamic imaging on surfaces of arbitrary shapes. The imaging process involves the initial estimation of surface profiles, which are subsequently utilized to design a spatial–temporal selective illumination sequence. To enable efficient axial scanning within a single frame exposure time, a spinning disc mechanism is employed, allowing for the acquisition of surface features with depth encoding (Fig. 1a and Supplementary Video 1). The acquired images, in conjunction with the corresponding illumination sequence, are then utilized to accurately decode cellular information.

We scan the focal length in a single exposure time. The spinning disc enables a rapid focus shift through continuous rotation, which is challenging in mesoscopic systems. To achieve high-resolution imaging with a large FOV during disc rotation, flat optics are employed instead of conventional lenses to change the position of the focal plane. By utilizing a spinning disc composed of glasses with varying thicknesses, a focus shift similar to the ‘broken pencil illusion’ in physics is induced^36^. All regions of the FOV are treated equally by the flat optical elements in a telecentric system, thereby enabling the spinning disc to change the focal length with high resolution across the entire FOV. Additionally, a temporal multiplexing detection method is employed, wherein a digital micromirror device (DMD) is utilized to spatiotemporally modulate the illumination of the sample. This ensures that regions with features are exclusively illuminated when they are in focus. The rotation of the spinning disc is monitored by an infra-red (IR) detector, recorded by a data acquisition device and synchronized with the DMD and camera.

The shape of the surface needs to be determined before image acquisition. Our previous work^37^ reviewed several types of depth detection approaches. Depth from focus/defocus infers scene depth from focus cues^38–43^. Focal sweep^44^, coded apertures^45^ and chromatic lens aberration^46^ approaches induce depth-independent PSFs to obtain the depth information. In this study, we adhere to the depth from focus framework for depth detection. As shown in Fig. 1, we first capture an image stack encompassing various depths, followed by the application of computer vision descriptors^47^ or machine learning approaches^48–50^ to identify the in-focus regions. Subsequently, the surface is reconstructed through smooth fitting and converted into DMD illumination patterns (Fig. 1b). For rapidly changing shapes, we provide an example of single-frame depth detection in Supplementary Note 4.1.

### MFIAS macroscopy enables micrometre-resolution and centimetre-scale imaging

**Fig. 2:**
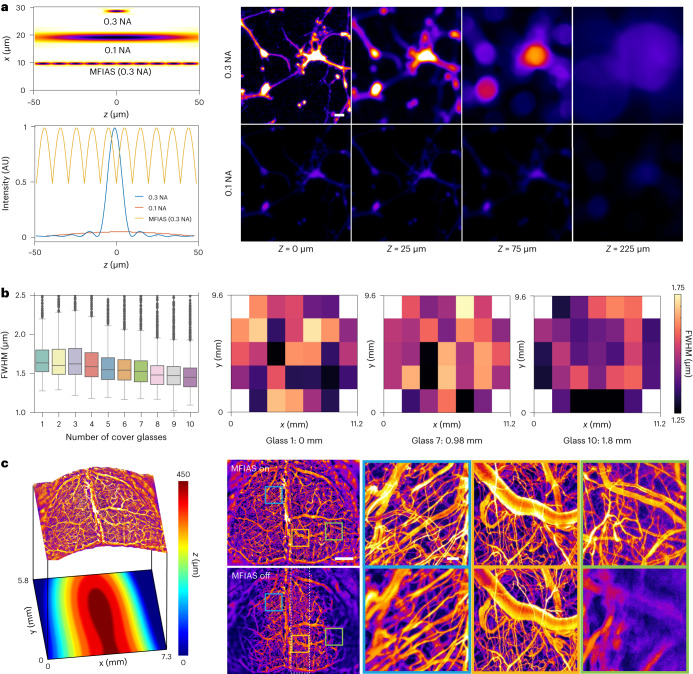
Characterization of the system. **a**, Theoretical simulations. Left top: PSFs of different systems. Systems with a high NA (0.3) have a high lateral resolution but a very small DOF (10 μm). Systems with low NA (0.1 NA) have low lateral resolution and a moderate DOF (90 μm). The MFIAS system achieves high NA and large DOF simultaneously. Left bottom: axial intensity distributions of different systems. Right: a simulation of a neuron with different levels of out-of-focus blur ranging from 0 to 225 μm. For high-NA systems, the image quality is acceptable within a 25-μm range of defocus, whereas for low-NA systems, the detection efficiency is reduced. Scale bar, 20 μm. **b**, Validation of MFIAS on RUSH system. Left: FWHM changes between 1.4 and 1.6 μm with the insertion of glass of different thicknesses, from 0 to 1.8 mm, measured with 0.5 μm fluorescent microspheres and 0.8 μm pixel size. Central black mark: median. Bottom and top edges: 25th and 75th percentiles. Whiskers extend to extreme points excluding outliers (1.5 times above or below the interquartile range). Right: FWHMs across the entire FOV (except for the four corner cameras, which are left blank in the figure) for glass thicknesses of 0 (minimal thickness), 0.98 mm (thickness for system design) and 1.8 mm (largest thickness). Beads number, *n* = 802, 961, 1,158, 1,269, 1,303, 1,294, 1,469, 1,435, 1,430 and 1,500 for each glass thickness. **c**, MFIAS enables the capture of a mouse brain image with FITC injection in vessels. Left: the brain image and its depth map. Middle: top projection of the mouse brain from MFIAS using a customized macroscope with SLR lenses. Three local zoom-ins on the right demonstrate that while conventional microscopes have out-of-focus blur, MFIAS enables globally all-in-focus imaging (*n* = 3). The dashed box indicates the in-focus area without MFIAS. Scale bars, 1 mm for the global views and 100 μm for the local views.

To compare the advantages of MFIAS macroscopy over conventional microscopes, we evaluated their PSFs using simulated fluorescent beads. Conventional microscopes typically face a trade-off between lateral resolution, DOF and light collection efficiency, as depicted in Fig. 2a. A microscope with an NA of 0.3 offers a lateral resolution of approximately 1 μm at the wavelength of 500 nm, but its DOF is limited, and the peak intensity drops notably at a distance of approximately 10 μm from the focal point. Conversely, objectives with a 0.1 NA provide a larger DOF of approximately 100 μm, but their lateral resolution is three times poorer compared with 0.3 NA objectives. Furthermore, the peak intensity drops to nearly 1% of that achieved by high-NA objectives. This necessitates larger pixel sizes or higher excitation power to compensate for photon loss, which also compromises optical resolution or increases photobleaching. Figure 2a further compares the simulated neural images captured by high- and low-NA systems at various focal depths. MFIAS scans the whole axial range and detects only in-focus signals, so it surpasses other solutions with its extensive DOF, high resolution and high photon efficiency at different depths.

To experimentally validate our predictions, we employed the RUSH macroscope equipped with a 0.3 NA objective^11^ (10 × 12 mm^2^ FOV, 1.2 μm resolution, 14,000 × 12,000 pixels) to provide a comprehensive demonstration of MFIAS’s capability for micrometre-resolution and centimetre-scale imaging. We tested the system’s parameters using 0.5-μm fluorescent beads positioned beneath cover glasses of varying thicknesses ranging from 0 to 1.8 mm. Supplementary Fig. 1 indicates a linear relationship between the focus shift and the cover glass thickness. By calculating the refractive index of the glass based on the focal plane shifts, we obtained a value of 1.54 ± 0.04 (mean ± standard deviation), consistent with the known refractive index of 1.52 for a wavelength of 515 nm. Additionally, we demonstrated that the lateral PSFs remained almost unchanged across the entire FOV, which is an advantage over conventional lens-based methods. The average full width at half maximum (FWHM) of the fluorescent beads, shown in Fig. 2b, exhibited a uniform distribution. Furthermore, Fig. 2b depicting FWHM distributions at different cover glass thicknesses confirmed that the PSFs did not deteriorate with increasing glass thickness.

**Extended Data Fig. 1:**
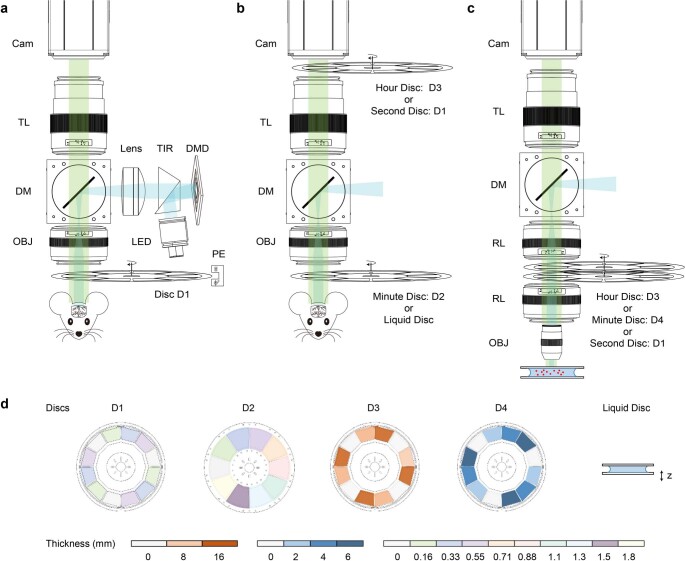
A Schematic diagram of the MFIAS-SLR systems. **a**, MFIAS-SLM can be adopted with a slight modification from a conventional SLR system. Cam, camera. TL, tube lens. DM, dichroic mirror. OBJ, objective. TIR, total internal reflection lens. LED, light-emitting diode. PE, photon emitter. **b**, The technique was extended by using multiple spinning discs in both the objective space and image space, including an “hour” disc in the image space for a large depth of field, a “minute” disc in the objective space as previously described, or a “second” disk for small shifts. Alternatively, a liquid disc can be used for continuous modulation. **c**, A Variation of the MFIAS Technique for High Numerical Aperture Systems. This configuration utilizes a 40X/0.95 objective and a 200-mm lens as the tube lens. To achieve quick refocusing, a pair of 50-mm lenses are inserted between the objective and the tube lens, and a set of spinning discs are placed between the 50-mm lenses. RL, Relay lens. **d**, The spinning discs were implemented with glass plates of different thicknesses. A liquid version of the disc is also shown.

**Extended Data Fig. 2:**
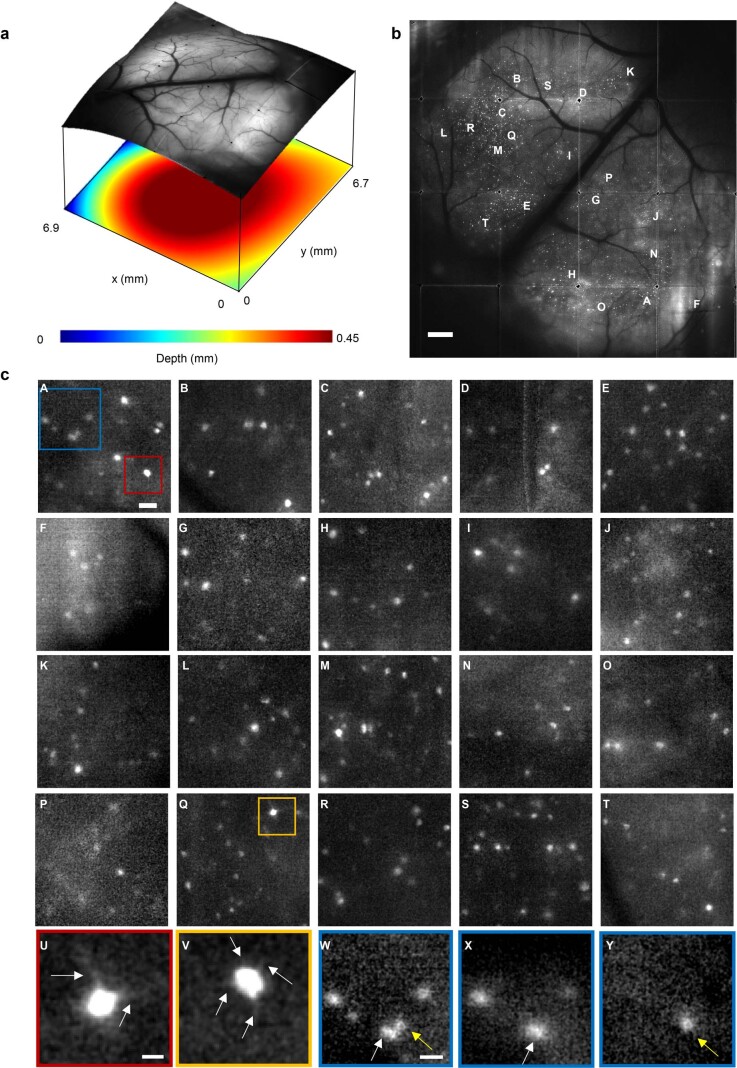
In vivo dynamic neural activity captured through MFIAS-RHSH microscopy. **a**, A surface plot of a snapshot image with the depth of each part plotted at the bottom. **b**, The standard deviation of the time sequence, which is captured by an sCMOS camera array at a rate of 10 frames per second. The original image size is 8352 ×8616 pixels. Scale bar, 1 mm. Sample size, n = 1 mouse. **c**, 20 subregions in panel (**b**). A – T, 20 subregions extracted from panel (**b**). Scale bar, 50 μm. U–V, Subregions extracted from (**c-A**) and (**c-Q**), where subcellular, dendrite-like structures can be observed. Scale bar, 10 μm. W, A subregion extracted from (**c-A**), in which two different cells with a lateral distance of 10 μm can be distinguished. X-Y, Highlights of the firing events of individual neurons. Scale bar, 25 μm.

MFIAS is a flexible technique that can be easily integrated into RUSH or other macroscopes, such as digital single lens reflex (SLR) systems^42^ (Extended Data Fig. 1a). The MFIAS-RUSH system is able to resolve subcellular structures and detect calcium signals on the curved mouse cortex at 10 frames per second (f.p.s.), as shown in Extended Data Fig. 2 and Supplementary Fig. 2. However, given the limited accessibility of the RUSH system to the broader scientific community, we also utilized MFIAS-SLR, a customized macroscope constructed from two SLR lenses, to showcase the advantages of MFIAS macroscopy. This system offers a lateral resolution of 7.4 μm and a FOV of 7 mm, enabling cellular resolution imaging in live mouse brains. To demonstrate this capability, we acquired image stacks from an awake, head-fixed mouse that had been injected with fluorescein isothiocyanate (FITC)–dextran^51^. The MFIAS microscope provided high-resolution images of the entire superficial brain, while conventional macroscopes could only focus on the central part of the brain, as shown in Fig. 2c and Supplementary Video 2.

### MFIAS macroscopy enables fast cellular-level functional imaging of the mouse brain

To demonstrate the superiority of MFIAS macroscopy over existing wide-field techniques, we employed MFIAS macroscopy on a customized macroscope to capture fast, micrometre-resolution spontaneous activity in the superficial dorsal cortex of the mouse brain. We conducted brain imaging on an awake Rasgrf2-2A-dCre;Ai148D mouse^52–54^ that expressed the calcium indicator GCaMP6f predominantly in superficial layer neurons (layer 2/3, as depicted in Supplementary Fig. 3). In single-photon microscopy, signals from deep neurons are heavily scattered by the brain tissue, so we focus on imaging neurons located at a specific depth of 130–200 µm below the dura^13^.

**Fig. 3:**
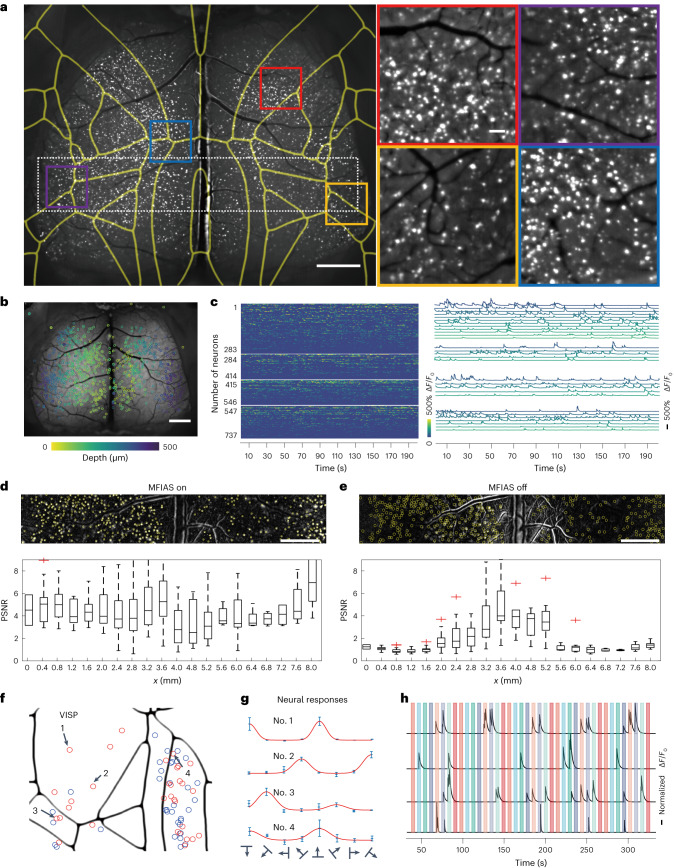
Images of a transgenic mouse brain that expresses GCaMP6f in neurons. **a**, The standard deviation of neural activity captured through MFIAS macroscopy and aligned with the Allen Mouse Brain Common Coordinate Framework. Four zoomed-in views are displayed on the right. Scale bars, 1 mm (global view) and 100 μm (local views). **b**, In the calibration stage, the mouse brain was axially scanned with different glass thicknesses. The neural position of each image depth was detected. Different colours in the image represent different glass thicknesses. Scale bar, 1 mm. **c**, The activities of 737 neurons from the four zoomed-in regions shown in **a**, sorted by *Z*-score. The right panel displays the traces of randomly selected neurons sorted from high to low *Z*-scores. Neural signals were detected in both central and corner regions using MFIAS macroscopy (sample size *n* = 6 mice). **d**, The distribution of PSNR for all sources across the FOV by MFIAS. **e**, The PSNR distribution for the same sources by a conventional microscope. The PSNR for the neural signal is almost constant for MFIAS but drops to the baseline at the lateral edge regions for the conventional microscope. **d** and **e** are representative micrographs out of three biological replicates obtained (source numbers 302, 337 and 668, in the 1,000-frame sequences), and the additional micrographs are included in Supplementary Note 6. Central black mark: median for neural sources. Bottom and top edges: 25th and 75th percentiles. Whiskers extend to extreme points excluding outliers (1.5 times above or below the interquartile range). Scale bars, 1 mm. **f**, Visual stimulus of drifting gratings was performed on the mouse, and visually responsive neurons in the visual cortex were detected in three mice using one-way ANOVA (*P* < 0.01). Blue circles, visually responsive but OSI <0.8 neurons. Red circles, OSI ≥0.8 neurons. **g**, The neural responses of four typical high OSI neurons in different brain areas. Error bars represent the trimmed standard deviation of the five trials. **h**, Traces of the neurons in **g**. Colour stripes in the figure represent periodic visual stimulus moments with angles of 270°, 135°, 315°, 225°, 0°, 180°, 45° and 90°. **e** and **g** are representative micrographs out of three biological replicates obtained, and the additional micrographs are included in Supplementary Note 6.

As demonstrated in Supplementary Video 3, MFIAS captured neural responses from the entire superficial cortex in a single snapshot. We identified cortex-wide light source distributions using the CNMF-E algorithm and deconvoluted calcium spikes from the calcium traces using the Online Active Set method to Infer Spikes algorithm (OASIS)^55,56^. The maximum imaging speed of MFIAS was limited by the current scientific complementary metal oxide semiconductor (sCMOS) camera speed, which could achieve up to 50 f.p.s. In our experiment, we set the speed to 10 Hz to match the dynamics of the calcium indicator. These light sources were evenly distributed across the entire FOV, as shown in Fig. 3a. In comparison, the conventional microscope with a 0.3 NA only detected light sources within its DOF under the same parameters (Supplementary Video 3). We also zoomed in on four selected regions in detail in Fig. 3a, where the contours of all light sources were visible at high resolution. Figure 3b illustrates that the light sources were located at different depths, which were detected from a series of 50 images at each depth. We obtained neural calcium signals from different sub-FOVs at different depths throughout the mouse brain, as depicted in Fig. 3c.

To compare the performance of MFIAS with conventional microscopes, we defined the peak signal-to-noise ratio (PSNR) of the light source as its peak signal intensity divided by the background standard deviation of the temporal variations. We plotted the PSNR distribution along the *x* axis for both MFIAS and conventional macroscopes in Fig. 3d,e. The study conducted with MFIAS observed that the PSNR of neural signals remained relatively constant. In contrast, when using conventional microscopes, the PSNR at the lateral edge dropped to the baseline PSNR of 1 (sample size *n* = 3 mice; additional micrographs are included in Supplementary Note 6.1). This suggests that MFIAS provides better signal quality on the curved brain surface than conventional microscopes. Furthermore, through visual stimulus, we detected visually responsive and orientation-selective sources in the visual cortex and plotted the distribution of these light sources in the visual-related areas of the mouse brain (additional micrographs are included in Supplementary Note 6.2). In all three mice, we observed a high percentage of orientation-selective sources in visually responsive sources in the primary visual cortex (VISP) area. Further, we show an example of neural imaging through the cleared skull^4^ in Supplementary Fig. 4.

MFIAS macroscopy can capture calcium signals from high-PSNR neurons in the sparsely labelled superficial mouse cortex. Previous studies have demonstrated that cellular calcium signals from a depth of 130–200 μm can be extracted using wide-field microscopy with 0.3 NA objectives^11,13^. These signals exhibit a high correlation with signals obtained from two-photon microscopy. The SLR system^42,43^ achieves a lateral resolution below 10 μm in a 7-mm-diameter FOV (Supplementary Note 1.3.2) and has demonstrated cellular resolution for fixed NSC-34 neurons^57^ (Supplementary Fig. 5). Supplementary Fig. 6 illustrates that the contour of two cells with a boundary distance of ~10 μm can be distinguished from the temporal standard deviation of the image stack. To further compare the calcium signals from the SLR lens and two-photon microscopy, we established a system for simultaneous single-photon and two-photon imaging. Similar to previous findings^13^, the analysis shows that a correlation of 0.8 can be achieved when the PSNR >1.5. However, signal crosstalk can occur from neighbouring neurons or out-of-focus neurons (Supplementary Note 3.2). In Rasgrf2-2A-dCre;Ai148D mouse^52,53^, sparse layer-specific GCaMP expression in the cortex reduces the cross-contamination between neurons. Furthermore, we demonstrated that the pairwise neural correlations from single-photon and two-photon microscopy exhibit similar strengths and patterns in Supplementary Note 3.3 when the cross-contamination and background signals have been properly eliminated by the CNMF-E algorithm. Finally, we show an example of analysing cortex-wide neural correlations from MFIAS imaging of a mouse in Supplementary Fig. 7a, which shows that the average pairwise correlations were close to zero in almost all brain regions, similar to the results from electrodes^58^. Similar to anatomical neural connectivity^59^, its functional neural correlations decrease with spatial distance (Supplementary Fig. 7b). The interactions between sources can be represented by the burst of spike trains. Supplementary Fig. 7c demonstrates this pattern, with a frequency of spikes that exhibits a long-tailed distribution compared with randomly shuffled spike trains^58^. As a result, MFIAS has provided a neural imaging approach for computational neuroscience.

### MFIAS macroscopy enables real-time tracking of neutrophils in blood vessels and on the surface of the mouse brain

To further demonstrate the potential of MFIAS macroscopy in in vivo cellular imaging, we conducted an immune cell tracking experiment in a B6/C57 mouse with a crystal skull implant. The procedure involved labelling the neutrophils by administering an intravenous injection of Ly-6G monoclonal antibody 2 h after the craniotomy^60,61^. Cells of interest were located at the surface of the cortex, allowing for their axial positions to be analysed with the aid of visible blood vessels.

Understanding the migration and collective behaviours of neutrophils is crucial for unravelling the mechanisms underlying acute inflammatory responses. Neutrophils, being the most abundant leukocyte type in human circulation, play a vital role in pathogen elimination during inflammation. However, uncontrolled neutrophil accumulation can lead to excessive inflammation by secreting chemoattractants that bind to G protein-coupled receptors on neighbouring cells^62^. The coordination and termination of these collective behaviours are still not fully understood. Conventional microscopy techniques enable the observation of immune cell trafficking from circulation to peripheral tissues in vivo, which is important for identifying the step-by-step process of neutrophil recruitment into inflamed tissues^62^. However, the observation of neutrophil migration using conventional microscopy is limited to the submillimetre scale due to FOV limitations^63^. In contrast, MFIAS macroscopy enables visualization of processes such as intravascular trafficking, vessel adhesion, surface locomotion, aggregation and dispersion in different brain regions, so researchers can gain further insights into the coordinated behaviours of neutrophils and better understand the underlying mechanisms of inflammation and the immune response.

**Fig. 4:**
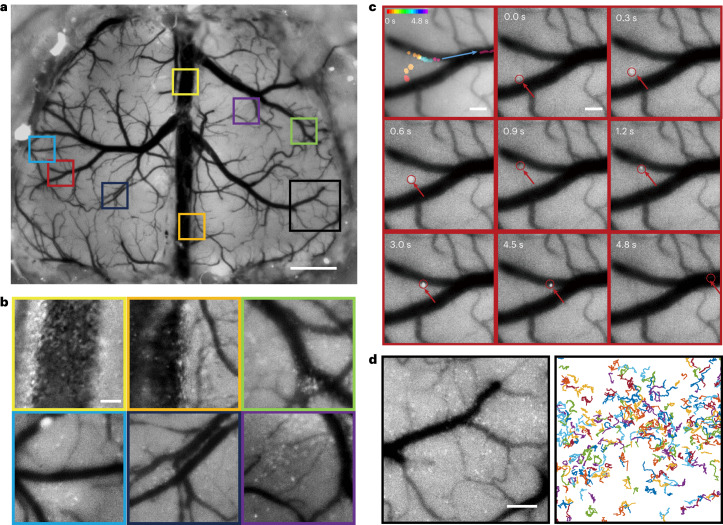
The recording of neutrophil trafficking in a mouse brain after craniotomy. **a**, MFIAS enables snapshot imaging of neutrophils across the dorsal cortex (sample size *n* = 4 mice). Scale bar, 1 mm. **b**, Zoomed-in views of the areas in **a**. Scale bar, 100 μm. **c**, A time-lapse sequence of a zoomed-in view in **a** showing an immune cell migrating from a capillary to a branch vessel. Scale bar, 100 μm. **d**, A zoomed-in view of an area in **a** showing the tracks of neutrophils. Scale bar, 200 μm.

**Extended Data Fig. 3:**
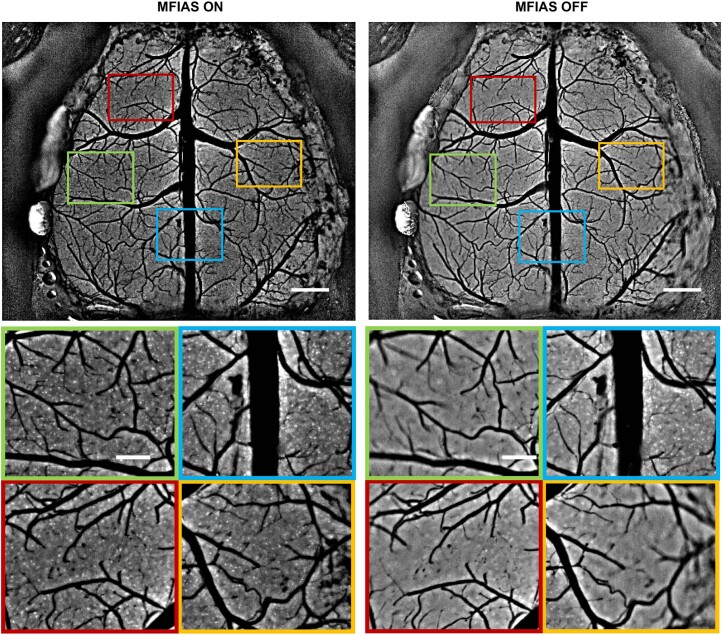
Imaging of immune cells in a Cx3cr1-GFP mouse. **a**, A global comparison of MFIAS and conventional microscopy. **b**, Local regions in **(a)**. The bright dots in the figures are microglia in the superficial cortex. Scale bars: 1 mm in **(a)** and 300 μm in **(b)**. Frame rate, 10 frames per second. Sample size, n = 1 mouse.

We demonstrated the capability of MFIAS macroscopy to capture events occurring at different time scales in Fig. 4a and Supplementary Video 4. Additionally, MFIAS macroscopy enables imaging of neural dynamics at various locations and depths within the brain, as illustrated in Fig. 4b. In the vasculature, we observed the flow of immune cells with blood at subsecond to second time scales across all mice in the study (sample size *n* = 4 mice). Notably, some cells exhibited adherence to vascular walls, indicating extravasation from the bloodstream, as the multistep process of ‘leukocyte adhesion cascade’^62^. Furthermore, we observed neutrophils in the superficial cortical tissue displaying migration patterns at time scales ranging from seconds to tens of seconds in three out of four mice. Previous studies have reported distinct neutrophil states in barrier tissues, wherein they exhibit random migration in the steady state but are rapidly recruited to the site of injury^64^. Neutrophil migration towards tissue injury involves a multistep process characterized by scouting, amplification, stabilization and resolution phases^65^. To investigate the state of neutrophils, we employed cellular motion tracking using TrackMate software^66^ in Fig. 4d. The analysis of 364 long tracks in the *xy* plane revealed various cellular dynamic properties (Supplementary Fig. 8). Although the averaged mean squared distance showed a linear relationship with time delay (Supplementary Fig. 8j), the medium velocity in the *xy* plane (Supplementary Fig. 8i), and the averaged autocorrelation (Supplementary Fig. 8m) was close to zero, abnormal superdiffusion and subdiffusion behaviours were observed in certain cells (Supplementary Fig. 8k), indicating diverse motion patterns during the early stage of swarming. As the local population of neutrophils increased, we observed collective motion^67^ in Supplementary Fig. 8f–i and cell aggregation in Supplementary Fig. 8a–c. In Fig. 4c, we show the observation of a cell aggregation moving at the junction of a branch vessel. The aggregation travels quickly in the deep capillary at the beginning but slows down when it nears the crossing. After the aggregation enters the branch vessel at 4 s, it accelerates again and flows away in less than 0.3 s. The migration of cell aggregates is a rare event that was observed only once and can hardly be observed for conventional systems due to the limited FOV. The ability of MFIAS macroscopy to simultaneously capture slow events in brain tissue and fast events in vessels makes it a valuable tool in the fields of immunology and haematology. We also showed the microglia in a Cx3cr1–GFP mouse^68^ in Extended Data Fig. 3.

### MFIAS achieves a high resolution in the millimetre-scale DOF with a modest trade-off from spherical aberration

**Extended Data Fig. 4:**
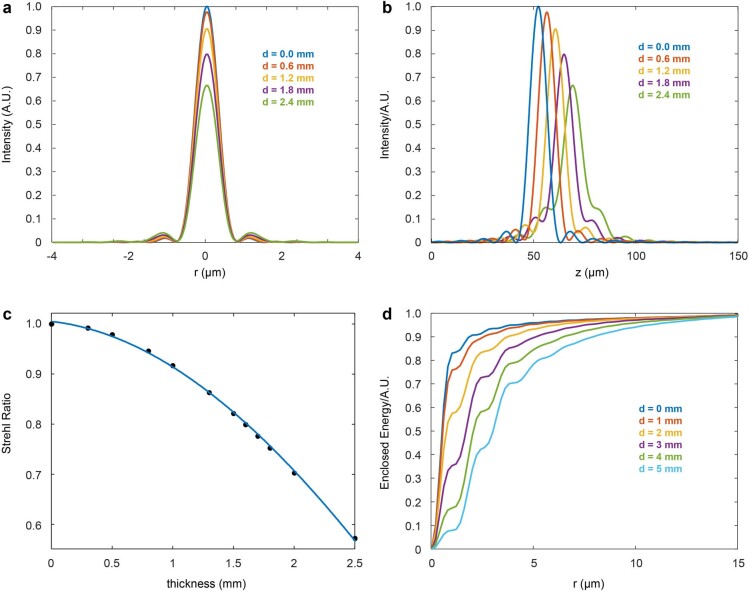
Analysis of spherical aberration in MFIAS. **a**, Variation in the lateral PSF profiles as the glass thickness d ranges from 0 to 2.4 mm. **b**, Variation in the axial PSF profiles with changing glass thickness. **c**, Strehl ratio of the point spread function with changing glass thickness. **d**, Enclosed energy in a circle as the radius varies from 0 to 15 μm, accounting for the additional cover glass.

The MFIAS microscope faces the issue of additional spherical aberration caused by the cover glass. To assess the impact of these additional cover glasses on the optical system, in Supplementary Note 1.1, we calculated the extra-optical path for a practical optical ray compared with a paraxial ray using the Debye approximation, as described in previous papers and textbooks^69–71^. Based on this work, we derived the expression for spherical aberration as $\left({{n}}^{2}-1\right)/\left(8{{n}}^{3}\right){\rm{d}}{{\rm{\theta }}}_{0}^{4}$, which approximates ${\rm{d}}{{\rm{\theta }}}_{0}^{4}/20$ for *n* = 1.5. We simulated the PSF and enclosed energy of the system under the condition of 0.3 NA (Extended Data Fig. 4a–d). The simulation involved varying glass thicknesses from 0 to 2.4 mm, corresponding to a focus shift from 0 to 0.8 mm. Previous research, such as SPED microscopy^22^, has demonstrated that, while the FWHM remains almost unchanged with increasing glass thickness, the energy disperses more with increasing glass thickness. In our simulation (Extended Data Fig. 4c), the Strehl ratio fell below 80% with a glass thickness of 1.8 mm (when an 80% Strehl ratio is considered diffraction limited in optical design^72^). However, over 90% of the total energy was still contained within a sample space circle with a radius of 5 μm (Extended Data Fig. 4d).

To increase the maximum focal shift, relay systems can be employed. Spherical aberration has a fourth-order dependence on the angle and a linear dependence on the thickness. For an imaging system with magnification *M*, the chief ray angle in the image space is 1/*M* of the angle in the objective space, while the axial displacement in the image space is *M*^2^ times that in the objective space. Consequently, for a glass plate, the induced focus shift in the image space is 1/*M*^2^ of that in the objective space due to the magnification *M* of the microscopic system, as illustrated in Supplementary Note 1.3. Therefore, the maximum focal shift can be increased by a factor of *M*^2^ if the cover glass is inserted in the imaging space. We further showed a proof-of-concept experiment that shows MFIAS can near-continuously scan the millimetre-scale depth-of-field (Extended Data Fig. 1b). This was achieved by designing multiple spinning discs in the objective and image spaces. The ‘minute’ disc refers to the disc in the objective space, the ‘hour’ disc refers to a disc with large-thickness glass slabs for large axial shifts, and the ‘second’ disc refers to a disc with small-thickness glass slabs in the image space for diffraction-limited point objects, which matches the DOF of the objective. The performance of the MFIAS system was evaluated using 0.5-μm fluorescent beads on a 3D-printed mouse brain model. The full depth-of-field of 1.8 mm was scanned by rotating the ‘hour’ disc (Supplementary Note 2). Additionally, the rotation of the ‘second’ disc resulted in near-continuous axial scanning.

## Discussion

The MFIAS macroscopy system offers a combination of extended DOF, large FOV and high-speed imaging across intricate surfaces while maintaining a lateral resolution comparable to that of conventional epifluorescence microscopes. The imaging speed of MFIAS macroscopy is primarily constrained by the frame rate of the camera and the rotational speed of the disc. Compared with other volumetric imaging techniques, such as light-field microscopy^28–32^ and spherical aberration-assisted extended depth-of-field microscopy^22^, MFIAS has high resolution and high detection efficiency without the need for deconvolution. It overcomes the limitations of other techniques by utilizing planar symmetry and converting the movement of a bulky objective into the rotation of a spinning disc, which allows for a large spatial-bandwidth product and fast response time. Data throughput is calculated as the scanned voxel points of the system, which equals to the product of resolved pixels on the plane and number of scanned pixels per second. The throughput is limited by the spatial bandwidth product of the lens and the response time of the system^73^. MFIAS scans 16.8 Giga voxels per second (14,000 pixels × 12,000 pixels × 10 layers × 10 f.p.s.), 100× more than the single-camera and single-planar acquisition configurations. Unlike previous configurations^36^, glass plates are perpendicular to the optical axis in the full FOV, allowing for ultrawide-field imaging in telecentric optical systems.

**Extended Data Fig. 5:**
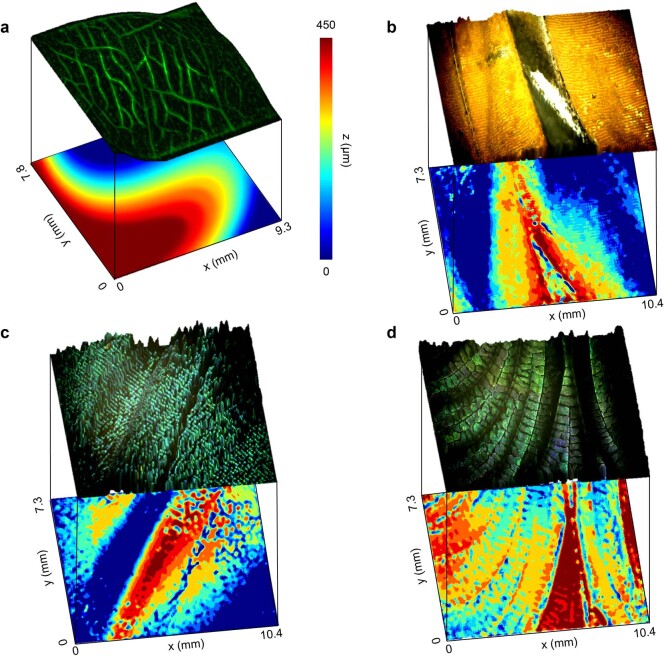
MFIAS images of complex surfaces. **a**, Image of FITC solution in a Scindapsus Aureus leaf. **b**-**d**, The wings of three butterflies captured in the reflective mode. Top images, reconstructed surfaces. Bottom images, depth maps.

We have also implemented other modes of MFIAS macroscopy. (1) Complex-shaped surfaces. We also explored the capabilities of MFIAS macroscopy on other complex-shaped surfaces. To illustrate this, we conducted an experiment involving the application of FITC solution to the stem of a fresh Epipremnum Aureum plant, followed by the capture of time-lapse images to observe the flow of fluorophores through the plant’s veins. We determined the surface profile of the leaf and then captured images at a rate of 5 Hz. Compared with conventional focal-stack acquisition methods, MFIAS macroscopy demonstrated the capability to rapidly access specific regions of interest by selectively illuminating and capturing in-focus areas. The results obtained from MFIAS macroscopy were compared with those obtained using a conventional microscope, as depicted in Extended Data Fig. 5a and Supplementary Fig. 9. (2) RGB mode. To further showcase the versatility of the MFIAS technique, we employed a reflective microscope equipped with white light illumination and an RGB camera. Using this setup, we captured images of the wings of distinct insect types, as presented in Extended Data Fig. 5b–d. This highlights the potential of our method for real-world applications, particularly in the realm of biomedical specimen diagnosis. (3) Continuous *z*-scan mode. Another approach to induce a continuous *z*-scan is to use a spiral plate with a thickness proportional to the polar angle. However, manufacturing such an optical element may cost a thousand times more than the off-the-shelf cover glass. We explored continuous *z*-scan mode using a liquid version of MFIAS as an alternative approach. Here the slow movement of heavy specimens or objectives was substituted with the rapid movement of the cover glass. The changes in liquid depth between the cover glasses induce continuous focus shifts in the specimen. The performance of this liquid MFIAS configuration was evaluated using 3-μm fluorescent microspheres, as depicted in Supplementary Note 2. (4) Short working distance mode. While the current implementation requires a working distance of several millimetres for the insertion of the spinning disc, we demonstrated its adaptability for systems with short working distances. For example, we present the design of MFIAS in a high-resolution system with an NA of 0.95, as shown in Extended Data Fig. 1c. Furthermore, we captured images of 200-nm fluorescent microspheres under various glass thicknesses in Supplementary Note 2. (5) Single-shot exposure surface detection. We developed different algorithms tailored to specific specimens. For surfaces that change slowly, such as brain tissue in head-fixed mice, we captured images or image stacks at different focal positions and subsequently identified the focused regions at each depth. For example, in neural imaging of the superficial mouse brain, we acquired 50 frames for each depth to discern neurons from the background. Through experimentation, we determined that a minimum of ten frames is needed, considering the calcium dynamics of neurons. For continuously changing surfaces, we proposed a subsecond single-exposure focus algorithm. This approach involves dividing the entire surface into small, non-overlapping patches, which are individually illuminated and detected at different depths. The surface profile is then estimated based on the focused patches, as elucidated in Supplementary Note 4.1. Additionally, we demonstrated the capability of high-speed scanning using dual-planar imaging, capturing 140 volumes per second, as demonstrated in Supplementary Note 4.2.

The MFIAS macroscopy system has some limitations. One such limitation pertains to the penetration depth, which is presently confined to imaging the superficial layer of cells within the mouse brain cortex due to tissue scattering. Previous investigations^13^ and Supplementary Note 3 have demonstrated that a single-photon microscope with an NA of 0.3 can effectively capture neural dynamics within a depth range of 130–200 μm, corresponding to superficial layer 2/3 neurons. In single-photon neural imaging, cross-section contamination from adjacent neurons and background noise elevates correlations in raw neural intensity data. Our experiment mitigates this contamination through a sparse superficial neural labelling scheme and signal extraction algorithm. Nonetheless, deep neural imaging experiments pose additional challenges that necessitate further investigation and optimization. Additionally, the system lacks optical sectioning capabilities, thereby precluding the capture of axial motion information. For instance, the MFIAS-SLR system can visualize cells within a restricted axial range of 80 μm. Consequently, when observing cells such as neutrophils, only their lateral speed can be ascertained, while their precise location above or below the dura remains indeterminate. Nevertheless, these limitations can be mitigated through the integration of optical sectioning techniques such as structured illumination^74,75^, HiLo^76^ and targeted illumination microscopy^17^. By employing these techniques, the MFIAS system can surmount its inherent constraints, thereby enabling the capture of axial information and expanding its imaging capabilities^77^. Another limitation of the MFIAS microscope pertains to its surface-detection speed. The system necessitates a calibration sequence and surface detection process to determine the surface profile, which can be time-consuming and precludes real-time imaging of rapidly varying surfaces. We found that the minimum time needed for calibration and surface detection is in the subsecond range, which means that this method is not suitable for responding to faster events. To achieve expedited imaging, potential avenues for exploration include the incorporation of an assistant camera for image acquisition and the development of hardware-based imaging processing algorithms.

In summary, MFIAS macroscopy provides the capability to observe wide-ranging neural and immune activities on both the awake mouse brain surface and other complex-shaped surfaces. This approach is cost-effective, is easy to implement and offers superior performance relative to conventional methods. Leveraging the versatile applications of spinning discs, MFIAS macroscopy may find uses across diverse fields, including neuroscience, immunology and clinical diagnosis. Furthermore, the potential for its extension into domains such as photography and stereo displays warrants consideration. We hope that the MFIAS system paves the way for the high-throughput dynamic recording of arbitrarily shaped surfaces, thereby enhancing the scope of life-science applications.

## Methods

### MFIAS implementation

MFIAS macroscopy is a technique for non-planar surface imaging on standard macroscopic systems. Briefly, MFIAS was developed on the basis of the principles of high-speed focal modulation and synchronized dynamic illumination selection. A custom spinning disc, as depicted in Supplementary Note 5, facilitated rapid focal position modulation. The disc was equipped with nine cover glasses of varying thicknesses (ranging from 0.17 to 1.8 mm), securely attached using ultraviolet-cured glue. To monitor the rotational speed of the disc, a stick connected to the disc was employed in conjunction with an IR emitter and detector system. The spinning disc was affixed to a rotation stage (IM6824H, Lika Tech), which was controlled by DC voltage. Placed between the objective and the specimen, the spinning disc played a crucial role in the modulation of focal positions. Dynamic illumination region selection was enabled through the utilization of a DMD (V-7001, Vialux) positioned in conjugation with the image plane. For each cover glass on the spinning disc, a distinct focal specimen region at the specific depth is precisely mapped onto a corresponding region on the DMD.

To construct a prototype of MFIAS-SLR microscopy, a custom macroscope setup was utilized. The objective lens employed in this setup was an SLR camera lens (Canon EF 50 mm f/1.4 USM). The tube lens consisted of a 100-mm camera lens (MINOLTA AF 100 mm f/2.8). For sample illumination, a collimated blue LED (SOLIS-470C, Thorlabs) was employed in conjunction with a shortpass excitation filter (FESH0500, Thorlabs) with a cut-off wavelength of 500 nm. The excitation beam was directed toward the DMD, which was aligned with the image plane. The beam was then relayed by an achromatic doublet (AC508-75-A, Thorlabs), reflected by a longpass dichroic mirror (DMLP505L, Thorlabs) with a cut-on wavelength of 505 nm, and subsequently passed through the objective lens to excite the sample. Fluorescence emitted by the sample was collected by the same objective lens, filtered by an emission filter (MF525-39, Thorlabs), and focused onto an sCMOS camera (Zyla 5.5, Andor) through the tube lens. Each pixel on the sCMOS camera corresponded to a spatial resolution of 3.6 µm on the image plane, providing a FOV of approximately 9 mm × 7 mm.

Another prototype of MFIAS macroscopy was constructed using the existing RUSH system^11^. The RUSH system is characterized by a customized 0.3 NA objective and a 0.17-gigapixel sCMOS array, enabling video-rate centimetre-scale imaging with 0.8-µm pixel size. This technology also serves as an excellent foundation for evaluating the PSF and FOV when combined with the spinning disc. A similar excitation light path was established to replace the illumination LED of the RUSH system and map the DMD to the native image plane of the RUSH system. A comprehensive guide for the design and implementation of MFIAS is available in Supplementary Note 5.

### Signal synchronization

The National Instrument USB-6363 data acquisition device was utilized for synchronization in the MFIAS macroscopy system, as depicted in Supplementary Note 5. The positional trigger of the spinning disc served as the input signal for the customized macroscope configuration, enabling control over both the camera exposure start and DMD transition. In the RUSH configuration, the camera output trigger was generated to trigger the DMD, and the phase and rotation frequency of the disc were calculated from the IR receiver signal. To ensure synchronization between the cameras and the spinning disc, proportional-integral-derivative control was implemented to regulate the voltage of the rotation stage.

### Object detection and surface fitting

In neural imaging, a series of 50–100 images were captured at different depths. To create an input image sequence, the temporal standard deviation of each image stack was calculated. In vessel and neutrophil imaging, a single image was acquired at each depth to serve as the input. The order of the image sequence was then rearranged based on the focus shift. To identify features such as cells within the images, the extended maximum transformation was applied using functions such as imextendedmax in MATLAB or the find Maxima function in ImageJ^78^. After identifying the cell regions, they were sorted on the basis of parameters such as area and eccentricity. For surface fitting, we used a 3D polynomial fitting for brain images and a smooth filter for features on other complex-shaped surfaces. Finally, the surface was discretized into ten height levels, and binary masks were created for each level.

### DMD and illumination calibration

Sparse bright pixels were displayed on the DMD and captured by the camera. The coordinates of these bright pixels were extracted using MATLAB software. Finally, an affine transformation was established between the camera pixels and DMD pixels using the fitgeotrans function in MATLAB.

### Selection of system parameters

To achieve the desired DOF, the MFIAS system employs a careful selection of cover glass number, glass thickness and detection objective NA. The glass thickness is determined based on the NA of the objective NA, the pixel size *p* and the feature size *d* of the specimen. For our system, the objective has an NA of 0.3, and the pixel sizes are approximately 0.8 μm and 3.6 μm for the RUSH and SLR systems, respectively. The diameter of a mouse neural soma is estimated to be approximately 15 μm, and the signals of the soma can be observed at a distance of *L =* 25 μm from the focal plane. Therefore, we set the interval of glass thickness to approximately 150 μm using the formula 2*nL*/(*n* − 1), where *n* represents the refractive index. Subsequently, the number of cover glasses is calculated as *N* = DOF/2*L* + 1 = 450/50 + 1 = 10. To maintain the same light intensity on the specimen as in conventional epifluorescence microscopy, the power of the excitation light needs to be increased by a factor of *N*, which corresponds to the number of cover glasses. By selectively illuminating only the in-focus regions, the MFIAS system reduces photobleaching and photodamage by a factor of *N* compared with traditional 3D scanning methods. Recent advancements in technology have led to the commercialization of high-speed spatial light modulators with 1,000-pixel resolution and a frame rate of 1,000 f.p.s., such as HSP1k-488-800-PC8 from Meadowlark Optics. These spatial light modulators can be used as alternatives to amplitude-modulating DMD to reduce the loss of light energy, albeit at a higher cost. Determining the minimum radius of the disc is crucial in the design process. The aperture of the glasses should be larger than the FOV or clear aperture of the lens, depending on whether the disc is positioned close to the sample plane or the lens. In this case, we select an aperture size of the glasses to be 40 mm, which exceeds the clear aperture of the lens. The minimum disc radius can be calculated, resulting in an approximate value of 100 mm. During the disc design, considerations such as weight balancing and maintaining the centre of mass close to the rotation axis are considered, as depicted in Supplementary Note 5.

### PSFs

The PSF of the system was measured using the RUSH system with a fluorescent microsphere specimen. The diameter of the beads was 0.5 µm, and approximately 10,000 beads were detected by our algorithm. The spinning disc was placed between the objective and specimen, and images were taken simultaneously from 35 cameras as the spinning disc was rotated to different positions.

### Multidisc MFIAS and high-NA MFIAS

The multidisc setup consisted of three discs designated the ‘minute’, ‘hour’ and ‘second’ discs. To achieve precise focal adjustments, a multiscale approach was adopted, wherein the minimum focus shift induced by the ‘minute’ disc was set to approximate the maximum focus shift caused by the ‘second’ disc. Similarly, the minimum focus shift induced by the ‘hour’ disc was aligned with the maximum focus shift caused by the ‘minute’ disc. The total number of scanning axial positions was determined by multiplying the number of glass plates in each disc: *m* plates in the ‘hour’ disc, *n* plates in the ‘minute’ disc and *k* plates in the ‘second’ disc, resulting in ${m}\times {n}\times {k}$ total positions. For this particular experiment, ${m}=3$, ${n}=10$ and ${k}=4$ were chosen.

In the high-NA configuration, an air objective lens with a magnification of 40× and an NA of 0.95 was utilized. The original tube lens was replaced with a 200-mm SLR lens, and a pair of 50-mm DSLR lenses was inserted as relay lenses between the objective and tube lens. To manipulate the focal depth, three discs were carefully inserted between the relay lenses.

### Animals

All animal procedures were performed by the Institutional Animal Care and Use Committee of Tsinghua University. C57BL/6 and transgenic mice, male and female, 8–12 weeks, 20–30 g, were obtained from the Jackson Laboratory. Mice were housed in standard cages with a maximum of five mice per cage. Cages were housed in an environment with a 12/12 h reverse dark/light cycle, an ambient temperature of 72 F and an ambient humidity of ~30%. Mice were provided food and water ad libitum. Both male and female mice used in the experiments were older than 8 weeks. C57BL/6J wild-type mice were injected with FITC–dextran (Sigma, MW (weighted average molar mass in gram per mole) 70,000, 2% w/v in saline, 200 mg kg^−1^) before blood vessel imaging. Cx3cr1–GFP transgenic mice (stock no. 005582, Jackson Laboratory), Ai148D transgenic mice (stock no. 030328, Jackson Laboratory) and Rasgrf2-2A-dCre transgenic mice (stock no. 022864, Jackson Laboratory) were maintained heterozygous or homozygous on a C57BL/6J background.

For microglial observation experiments, we used Cx3cr1–GFP mice. For transgenic neural activity observation experiments, we used transgenic Rasgrf2-2A-dCre; Ai148D mice expressing GCaMP6f in cortical layer 2/3 and C57BL/6J mice sparsely labelled with GCaMP6f viruses in the cortex. Double transgenic Rasgrf2-2A-dCre; Ai148D mice were crossed from Ai148D and Rasgrf2-2A-dCre mice, specifically labelled layer 2/3 cortical neurons. Before surgery, trimethoprim (TMP, 0.25 mg g^−1^, CAS#738-70-5, Sigma) was intraperitoneally injected for 2 days to induce the expression of GCaMP6f in layer 2/3. Adult C57BL/6 mice were sparsely labelled with ten injections of a mixture of diluted AAV2-9-hSyn-cre and AAV2-9-Ef1a-DIO-GCaMP6f viruses (from BrainVTA Technology). For neutrophil imaging, 10 μl Alexa Fluor 488 anti-mouse Ly-6G (eBioscience, Cas#127626, lot B350441) dissolved in 200 μl physiological saline was injected into the C57BL/6J mice by intravenous injection 15 min before imaging.

### Optical window

A flat optical cover glass was first trimmed into a trapezoidal shape (two bases *a*_1_ = 6 mm, *a*_2_ = 10 mm, height *h* = 6.5 mm). Then, it was heated on a stainless-steel mould in the shape that was fitted to the mouse skull (*h* = −0.06*x*^2^ − 0.0007*x*^4^, where the unit of *x* is millimetre).

### Craniotomy

All animal procedures were performed by the Institutional Animal Care and Use Committee of Tsinghua University. All experimental mice were anaesthetized with isoflurane (3%) delivered through an oxygen flow rate of 0.5 l min^−1^. They were then placed in a stereotaxic frame (RWD) and kept under anaesthesia with 1–1.5% isoflurane. The surgery was conducted under sterile conditions using sterile instruments. The mice were kept warm on a heating pad to maintain a body temperature of 36.5 °C. The scalp was shaved and removed, and the skin was anaesthetized with 0.1 ml of lidocaine. The fascia above the skull was also removed and cleaned with saline. A trapezoid area of skull that covered the dorsal cortex was removed with a drill and replaced with a crystal skull. The skin incision and crystal skull edge were sealed with cyanoacrylate (Vetbond, 3 M). The location of the crystal skull was recorded relative to the bregma. A head bar was implanted and secured to the skull with dental cement. The mice were given anti-inflammatory drugs (flunixin meglumine, 1.25 mg kg^−1^, and meloxicam, 5 mg kg^−1^) for 3 days after the surgery and were ready for imaging after 7 days of recovery.

### PSNR of light sources

The PSNR is defined as the ratio of the peak value of each light source to the background intensity fluctuation. The peak value was achieved by the maximum intensity of pixels in each light source, and the noise level was estimated from the temporal standard derivation of the 2D-Gaussian filtered image (kernal size σ = 10 pixels). The find Maxima function in ImageJ was utilized for the purpose of locating the positions of soma-like objects, employing manually selected thresholds to ensure accurate detection. To mitigate potential bias, the PSNR calculation incorporated neural positions from both MFIAS images and conventional images.

### Detection of soma position

A spatial–temporal Laplacian of Gaussian filter (kernel sizes *σ*_*x*1_ = *σ*_*y*1_ = 2 pixels, *σ*_*x*2_ = *σ*_*y*2_ = 15 pixels, *σ*_*t*1_ = 10 frames, *σ*_*t*2_ = 30 frames) was employed to remove the background and highlight the soma of neurons because a neuron should have a higher signal level than background pixels. Then, the neural positions were fitted from the temporal standard derivation project, according to the neural shape.

### Comparison of 1p and 2p microscopy

To validate the capability of our single-photon microscope in detecting single cellular activities, we developed a system that sequentially detects both single-photon (1p) and two-photon (2p) signals within each frame. This system configuration is illustrated in Supplementary Note 3. The detection path includes a beam splitter that allows us to detect both signals in each frame. To minimize contamination from different neurons, we implemented sparse labelling schemes by controlling the depth of GCaMP expression in transgenic mice and/or decreasing the level of virus titter, as described in our previous works^11,79^.

### Visual orientation selectivity assay

Mice were exposed to sinusoidal visual gratings using a small LCD display (14 cm × 10 cm) mounted horizontally. The display was centered at 7.5 cm in front of the right eye of the mouse at a 30° offset. To prevent stray light from reaching the cranial window, a light-blocking cone was attached to the animal’s head bar. A movie of grey sinusoidal gratings was presented sequentially, with eight stimuli separated by 45°. Each stimulus lasted 4 s with a 4-s intertrial interval, and they were presented five times in the same order. The grating pattern had a spatial frequency of 0.05° and a temporal frequency of 2 Hz.

The calculation of the orientation selectivity index (OSI) involved several steps. Initially, a one-way analysis of variance (ANOVA) was performed to compare the average response with the grating stimuli with the response during blank periods (*P* < 0.01). For these visually responsive cells, the preferred orientation was determined as the stimulus that elicited the strongest response. To construct the orientation tuning curve, the average Δ*F*/*F*_0_ was measured for each orientation throughout the stimulus duration. The tuning curve was then fitted with two Gaussians centred on *θ*_pref_ and *θ*_pref_ ± 90°. We used the following definition of the OSI: OSI = (*r*_pref_ − *r*_orth_)/(*r*_pref_ + *r*_orth_), where *r*_pref_ is the maximum trial-averaged fluorescence in response to any grating orientation and *r*_orth_ is the minimal response to the 90° offset grating using the fluorescence traces from CNMF-E. In the cortex-wide data analysis, we applied the raw pixel intensity at the detected neuron centre minus a background signal estimated from the spatial average of a 15-pixel-size Gaussian kernel. Then, we fitted the trace by the OASIS to filter the noise and achieved the calcium signal for each neuron. The trimmed standard deviation is calculated from the average trimmed sum of squared deviations around the mean of the visual response excluding the maximum and minimal extreme trials.

### Cell lines

The NSC-34 cell samples were gifts from Prof. Qihui Fan’s laboratory. Cells were purchased from Hunan Fenghui Biotechnology. Before imaging, they were exposed to 1 μM calcein-AM (lot KU723, Dojindo) and incubated at 37 °C for 20 min. After 20 min, the cells were washed with phosphate-buffered saline and then fixed with 4% paraformaldehyde solution for 30 min.

### Image analysis

For vasculature imaging, the temporal standard deviations of the sequence were taken, and the contrast was enhanced using the CLANE algorithm from ImageJ^80^. Neural images were taken from the standard deviation of a background-subtracted, time-filtered time sequence, and signals were extracted using the CNMF-E algorithm. Calcium spikes are deconvoluted from the calcium traces using the OASIS algorithm: AR1 model, foosi method, minimal spike size −5, maximum decay time 10 s. Neutrophil images were filtered in time using a Gaussian filter, and traces were extracted with the TrackMate ImageJ plug-in^66^. MSD and velocity correlations were calculated using msdanalyzer^81^. Simulation of PSF was performed with the code adapted from ref. ^82^.

### Statistics and reproducibility

Sample sizes and statistics are reported in the figure legends and text for each measurement. The sample size was not determined using a statistical method. All attempts at replication were successful, and each main result was at least a duplicate of experiments. Representative imaging results are included in the figures and videos to show the imaging performance. Statistics were performed using built-in MATLAB functions.

### Reporting summary

Further information on research design is available in the Nature Portfolio Reporting Summary linked to this article.

### Supplementary information

Supplementary Information Supplementary figures, notes and video captions.

Reporting Summary

Supplementary Video 1 Animation of the working principle.

Supplementary Video 2 Cortex-wide vasculature imaging in the mouse brain.

Supplementary Video 3 Cortex-wide neural imaging in the mouse brain.

Supplementary Video 4 Cortex-wide immune imaging in the mouse brain.

## Data Availability

The main data supporting the results in this study are available within the paper and its Supplementary Information. Data generated in this study, including source data for the figures, are available from Figshare with the following identifiers: source data for the figures, 10.6084/m9.figshare.24431824; beads data, 10.6084/m9.figshare.20103707; neuron data, 10.6084/m9.figshare.20103749; neutrophil data, 10.6084/m9.figshare.20103791; vasculature data, 10.6084/m9.figshare.20103716. The raw and analysed datasets generated during the study are too large to be publicly shared, yet they are available for research purposes from the corresponding authors on reasonable request.

## Code availability

The custom code for surface detection is available at 10.5281/zenodo.10034030. The custom code for image acquisition is available at 10.5281/zenodo.10033824. Additional custom algorithms and software are available at https://github.com/Crazyonxh/SMART-microscopy.

## References

[1] Kim TH (2016). Long-term optical access to an estimated one million neurons in the live mouse cortex. Cell Rep..

[2] Ghanbari L (2019). Cortex-wide neural interfacing via transparent polymer skulls. Nat. Commun..

[3] Guo ZV (2014). Flow of cortical activity underlying a tactile decision in mice. Neuron.

[4] Li D (2022). A through-intact-skull (TIS) chronic window technique for cortical structure and function observation in mice. eLight.

[5] Wang J, Zhang Y, Xu TH, Luo QM, Zhu D (2012). An innovative transparent cranial window based on skull optical clearing. Laser Phys. Lett..

[6] Zhao Y-J (2018). Skull optical clearing window for in vivo imaging of the mouse cortex at synaptic resolution. Light Sci. Appl..

[7] Kong L (2015). Continuous volumetric imaging via an optical phase-locked ultrasound lens. Nat. Methods.

[8] Demas J (2021). High-speed, cortex-wide volumetric recording of neuroactivity at cellular resolution using light beads microscopy. Nat. Methods.

[9] Sofroniew NJ, Flickinger D, King J, Svoboda K (2016). A large field of view two-photon mesoscope with subcellular resolution for in vivo imaging. eLife.

[10] Nöbauer T (2017). Video rate volumetric Ca^2+^ imaging across cortex using seeded iterative demixing (SID) microscopy. Nat. Methods.

[11] Fan J (2019). Video-rate imaging of biological dynamics at centimetre scale and micrometre resolution. Nat. Photonics.

[12] Zhou P (2018). Efficient and accurate extraction of in vivo calcium signals from microendoscopic video data. eLife.

[13] Zhang Y (2023). Rapid detection of neurons in widefield calcium imaging datasets after training with synthetic data. Nat. Methods.

[14] Shi R (2019). Multi-plane, wide-field fluorescent microscopy for biodynamic imaging in vivo. Biomed. Opt. Express.

[15] Botcherby EJ, Juškaitis R, Booth MJ, Wilson T (2008). An optical technique for remote focusing in microscopy. Opt. Commun..

[16] Anselmi F, Ventalon C, Bègue A, Ogden D, Emiliani V (2011). Three-dimensional imaging and photostimulation by remote-focusing and holographic light patterning. Proc. Natl Acad. Sci. USA.

[17] Xiao S, Tseng H-A, Gritton H, Han X, Mertz J (2018). Video-rate volumetric neuronal imaging using 3D targeted illumination. Sci. Rep..

[18] Pozzi P (2020). Plug-and-play adaptive optics for commercial laser scanning fluorescence microscopes based on an adaptive lens. Opt. Lett..

[19] Bonora S (2015). Wavefront correction and high-resolution in vivo OCT imaging with an objective integrated multi-actuator adaptive lens. Opt. Express.

[20] Kauvar IV (2020). Cortical observation by synchronous multifocal optical sampling reveals widespread population encoding of actions. Neuron.

[21] Hattori A, Yasuda K (2012). Extended depth of field optics for precise image analysis in microfluidic flow cytometry. Jpn J. Appl. Phys..

[22] Tomer, R. et al. SPED light sheet microscopy: fast mapping of biological system structure and function. *Cell* **163**, 1796–1806 (2015). 10.1016/j.cell.2015.11.061 PMC4775738 26687363

[23] Fahrbach FO, Simon P, Rohrbach A (2010). Microscopy with self-reconstructing beams. Nat. Photonics.

[24] Snoeyink C, Wereley S (2013). Single-image far-field subdiffraction limit imaging with Axicon. Opt. Lett..

[25] Tucker SC, Cathey WT, Dowski ER (1999). Extended depth of field and aberration control for inexpensive digital microscope systems. Opt. Express.

[26] Marks DL, Stack RA, Brady DJ, Gracht J (2011). Three-dimensional tomography using a cubic-phase plate extended depth-of-field system. Opt. Lett..

[27] Abrahamsson, S., Usawa, S. & Gustafsson, M. A new approach to extended focus for high-speed high-resolution biological microscopy. In *Proc. SPIE Three-Dimensional and Multidimensional Microscopy: Image Acquisition and Processing XIII* Vol. 6090 (SPIE, 2006).

[28] Lin X, Wu J, Zheng G, Dai Q (2015). Camera array based light field microscopy. Biomed. Opt. Express.

[29] Guo C, Liu W, Hua X, Li H, Jia S (2019). Fourier light-field microscopy. Opt. Express.

[30] Levoy, M., Ng, R., Adams, A., Footer, M. & Horowitz, M. Light field microscopy. *ACM Trans. Graphics* **25**, 924–934 (2006).

[31] Li H (2019). Fast, volumetric live-cell imaging using high-resolution light-field microscopy. Biomed. Opt. Express.

[32] Prevedel R (2014). Simultaneous whole-animal 3D imaging of neuronal activity using light-field microscopy. Nat. Methods.

[33] Kazemipour A (2019). Kilohertz frame-rate two-photon tomography. Nat. Methods.

[34] Song A (2017). Volumetric two-photon imaging of neurons using stereoscopy (Vtwins). Nat. Methods.

[35] Zhou KC (2023). Parallelized computational 3D video microscopy of freely moving organisms at multiple gigapixels per second. Nat. Photonics.

[36] Zhang Z (2021). Imaging volumetric dynamics at high speed in mouse and zebrafish brain with confocal light field microscopy. Nat. Biotechnol..

[37] Lin, X., Suo, J., Wetzstein, G., Dai, Q. & Raskar, R. Coded focal stack photography. In *Proceedings of 2013 IEEE International Conference on Computational Photography (ICCP)* 1–9 (IEEE, 2013).

[38] Favaro, P., Soatto, S. *3-D Shape Estimation and Image Restoration: Exploiting Defocus and Motion-Blur* (Springer Science & Business Media, 2007).

[39] Nayar SK, Nakagawa Y (1994). Shape from Focus. IEEE Trans. Pattern Anal. Mach. Intell..

[40] Namboodiri, V. P. & Chaudhuri, S. Recovery of relative depth from a single observation using an uncalibrated (real-aperture) camera. In *Proceedings of the**IEEE Conference on Computer Vision and Pattern Recognition* 1–6 (2008).

[41] Favaro, P. Recovering thin structures via nonlocal-means regularization with application to depth from defocus. In *Proceedings of the**IEEE Computer Society Conference on Computer Vision and Pattern Recognition* 1133–1140 (2010).

[42] Zhou Q (2022). Three-dimensional wide-field fluorescence microscopy for transcranial mapping of cortical microcirculation. Nat. Commun..

[43] Chen Z, Zhou Q, Rebling J, Razansky D (2020). Cortex-wide microcirculation mapping with ultrafast large-field multifocal illumination microscopy. J. Biophotonics.

[44] Kuthirummal S, Nagahara H, Zhou C, Nayar SK (2011). Flexible depth of field photography. IEEE Trans. Pattern Anal. Mach. Intell..

[45] Levin, A., Fergus, R., Durand, F. & Freeman, W. T. Image and depth from a conventional camera with a coded aperture. *ACM Trans. Graph.* **26**, 70 (2007).

[46] Cossairt, O. & Nayar, S. Spectral focal sweep: extended depth of field from chromatic aberrations. In *Proceedings of 2010 IEEE International Conference on Computational Photography (ICCP)* 1–8 (IEEE, 2010).

[47] Kong H, Akakin HC, Sarma SE (2013). A generalized Laplacian of Gaussian filter for blob detection and its applications. IEEE Trans. Cybern..

[48] Ronneberger, O., Fischer, P. & Brox, T. U-net: convolutional networks for biomedical image segmentation. In *MICCAI* (eds Navab, N. et al.) 234–241 (Springer, 2015).

[49] Falk T (2019). U-Net: deep learning for cell counting, detection, and morphometry. Nat. Methods.

[50] Zhou, Z., Rahman Siddiquee, M. M., Tajbakhsh, N. & Liang, J. UNet++: a nested U-Net architecture for medical image segmentation. In *Deep Learning in Medical Image Analysis and Multimodal Learning for Clinical Decision Support* 3–11 (Springer, 2018). 10.1007/978-3-030-00889-5_1 PMC7329239 32613207

[51] Spadoni I (2015). A gut–vascular barrier controls the systemic dissemination of bacteria. Science.

[52] Daigle TL (2018). A suite of transgenic driver and reporter mouse lines with enhanced brain-cell-type targeting and functionality. Cell.

[53] Harris JA (2014). Anatomical characterization of cre driver mice for neural circuit mapping and manipulation. Front. Neural Circuits.

[54] Zhang Y (2021). Computational optical sectioning with an incoherent multiscale scattering model for light-field microscopy. Nat. Commun..

[55] Friedrich J, Zhou P, Paninski L (2017). Fast online deconvolution of calcium imaging data. PLoS Comput. Biol..

[56] Friedrich J, Paninski L (2016). Fast active set methods for online spike inference from calcium imaging. Adv. Neural Inf. Process. Syst..

[57] Matusica D, Fenech MP, Rogers ML, Rush RA (2008). Characterization and use of the NSC‐34 cell line for study of neurotrophin receptor trafficking. J. Neurosci. Res..

[58] Ecker AS (2010). Decorrelated neuronal firing in cortical microcircuits. Science.

[59] Ercsey-Ravasz M (2013). A predictive network model of cerebral cortical connectivity based on a distance rule. Neuron.

[60] Dong G (2021). Low-dose Il-2 treatment affords protection against subarachnoid hemorrhage injury by expanding peripheral regulatory T cells. ACS Chem. Neurosci..

[61] Brooke CB, Deming DJ, Whitmore AC, White LJ, Johnston RE (2010). T cells facilitate recovery from venezuelan equine encephalitis virus-induced encephalomyelitis in the absence of antibody. J. Virol..

[62] Kolaczkowska E, Kubes P (2013). Neutrophil recruitment and function in health and inflammation. Nat. Rev. Immunol..

[63] Chèvre R (2014). High-resolution imaging of intravascular atherogenic inflammation in live mice. Circ. Res..

[64] Ng LG (2011). Visualizing the neutrophil response to sterile tissue injury in mouse dermis reveals a three-phase cascade of events. J. Invest. Dermatol..

[65] Weninger W, Biro M, Jain R (2014). Leukocyte migration in the interstitial space of non-lymphoid organs. Nat. Rev. Immunol..

[66] Tinevez J-Y (2017). TrackMate: an open and extensible platform for single-particle tracking. Methods.

[67] Lämmermann T (2016). In the eye of the neutrophil swarm—navigation signals that bring neutrophils together in inflamed and infected tissues. J. Leukoc. Biol..

[68] Jung S (2000). Analysis of fractalkine receptor Cx3cr1 function by targeted deletion and green fluorescent protein reporter gene insertion. Mol. Cell. Biol..

[69] Born, M. & Wolf, E. *Principles of Optics: Electromagnetic Theory of Propagation, Interference and Diffraction of Light* (Elsevier, 2013).

[70] Braat J (1997). Influence of substrate thickness on optical disk readout. Appl. Opt..

[71] Braat J (1997). Analytical expressions for the wave-front aberration coefficients of a tilted plane-parallel plate. Appl. Opt..

[72] Ottevaere, H. & Thienpont, H. Optical microlenses. In *Encyclopedia of Modern Optics* Vol. 4 (ed. Guenther, R. D.) 21–43 (Elsevier, 2005).

[73] Lohmann AW, Dorsch RG, Mendlovic D, Zalevsky Z, Ferreira C (1996). Space–bandwidth product of optical signals and systems. J. Opt. Soc. Am. A.

[74] Parot VJ (2019). Compressed Hadamard microscopy for high-speed optically sectioned neuronal activity recordings. J. Phys. D.

[75] Gustafsson MGL (2000). Surpassing the lateral resolution limit by a factor of two using structured illumination microscopy. J. Microsc..

[76] Lim D, Ford TN, Chu KK, Mertz J (2011). Optically sectioned in vivo imaging with speckle illumination hilo microscopy. J. Biomed. Opt..

[77] Abouakil F (2021). An adaptive microscope for the imaging of biological surfaces. Light. Sci. Appl..

[78] Soille, P. *Morphological Image Analysis: Principles and Applications* (Springer, 2003).

[79] Xu N-L (2012). Nonlinear dendritic integration of sensory and motor input during an active sensing task. Nature.

[80] Schneider CA, Rasband WS, Eliceiri KW (2012). NIH Image to ImageJ: 25 years of image analysis. Nat. Methods.

[81] Tarantino, N. et al. TNF and IL-1 exhibit distinct ubiquitin requirements for inducing NEMO–IKK supramolecular structures. *J. Cell Biol.* **204**, 231–245 (2014). 10.1083/jcb.201307172 PMC3897181 24446482

[82] Latychevskaia T, Schachtler D, Fink H-W (2016). Creating airy beams employing a transmissive spatial light modulator. Appl. Opt..

