# Supplementary material for: Multifocal fluorescence video-rate imaging of centimetre-wide arbitrarily shaped brain surfaces at micrometric resolution
Source: Nat Biomed Eng. 2023 Dec 6;8(6):740–53. doi: 10.1038/s41551-023-01155-6 (PMC11250366; doi:10.1038/s41551-023-01155-6)
Supplement: Supplementary file 1 — Supplementary figures, notes and video captions. [file 41551_2023_1155_MOESM1_ESM.pdf]

nature biomedical engineering

Article https://doi.org/10.1038/s41551-023-01155-6

Multifocal fluorescence video-rate imaging of centimetre-wide arbitrarily shaped brain surfaces at micrometric resolution

In the format provided by the authors and unedited

Contents

Supplementary Fig. 1 | Focal shifts resulting from varying glass thickness.

Supplementary Fig. 2 | MFIAS-RUSH imaging of a mouse brain injected with virus and expressing GCaMP in neurons.

Supplementary Fig. 3 | Spatial distribution of neurons in a brain slice obtained from a Rasgrf2-2Adcre;Ai148D mouse.

Supplementary Fig. 4 | Neural imaging of a Rasgrf2-2A-dcre; Ai148D Mouse with a cleared skull.

Supplementary Fig. 5 | NSC-34 cells stained with calcein-AM and observed using the MFIAS-SLR system.

Supplementary Fig. 6 | Detection of cortex-wide neural sources using MFIAS-SLR.

Supplementary Fig. 7 | Analysis of single neural sctivity obtained through MFIAS Imaging .

Supplementary Fig. 8 | Aggregation of neuphophil captured with the MFIAS-SLR system.

Supplementary Fig. 9 | MFIAS images of FITC solution in a leaf.

Supplementary Note 1 | Abberation calculation and PSF Measurements.

Supplementary Note 2 | Depth extension.

Supplementary Note 3 | Comparison of 1p and 2p neural detections.

Supplementary Note 4 | Speed of surface detection and image acquisition.

Supplementary Note 5 | A protocol for building MFIAS.

Supplementary Note 6 | Replications of biological experiments.

Supplementary Note 7 | Comparison of MFIAS and other SOTA techniques.

Supplementary Note 8 | Experimental parameters.

Supplementary Video 1 | Animation of the working principle.

Supplementary Video 2 | Cortex-wide vasculature imaging in the mouse brain.

Supplementary Video 3 | Cortex-wide neural imaging in the mouse brain.

Supplementary Video 4 | Cortex-wide immune imaging in the mouse brain.

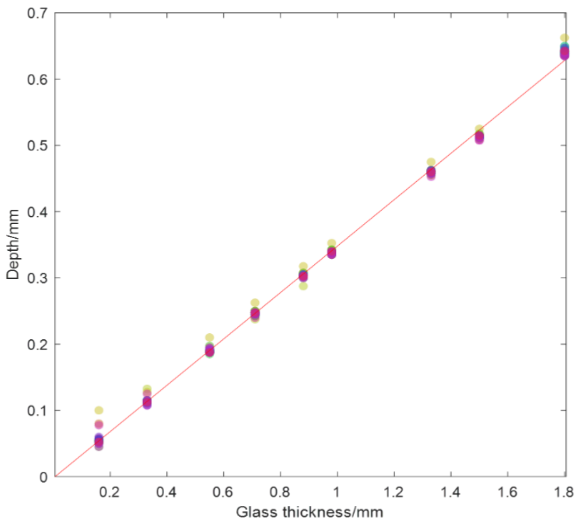

Supplementary Fig. 1 | Focal shifts resulting from varying glass thickness. The focal planes of 31 cameras were shifted upon the insertion of glass, with each camera represented by a distinct color in the RUSH dataset. The slope of the data points allows for the calculation of the refractive index, yielding a value of 1.54 ± 0.04 (mean ± s.d.). The overlapping of the focus shifts across different cameras indicates that the system has consistent performance throughout the entire field of view.

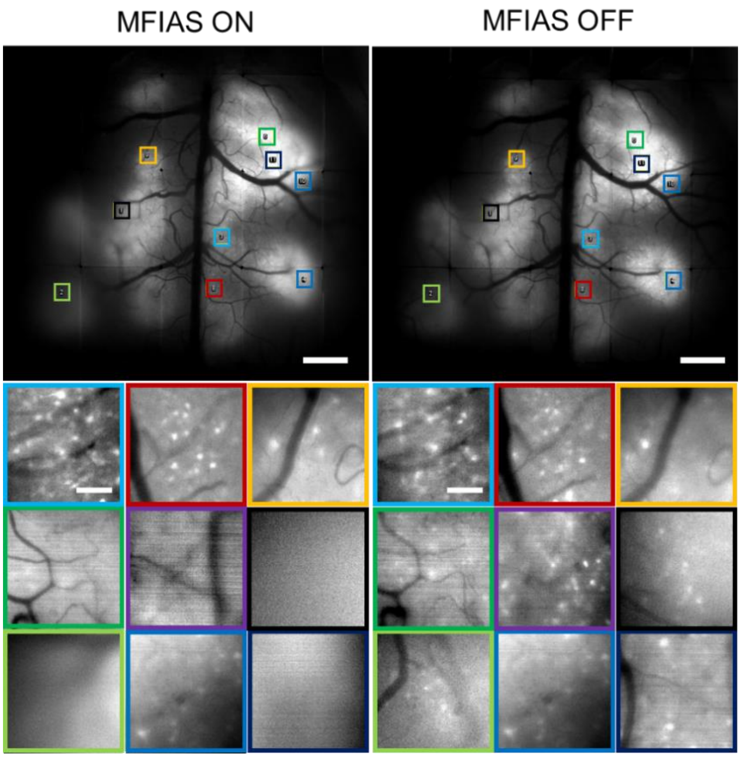

Supplementary Fig. 2 | MFIAS-RUSH imaging of a mouse brain injected with virus and expressing GCaMP in neurons. The temporal standard deviation of the image sequences was demonstrated. Ten injections were strategically distributed throughout the mouse cortex. Top, global views. Bottom, local views. Scale bars, 1 mm in global views and 100 μm in local views. Sample size, n=1 mouse.

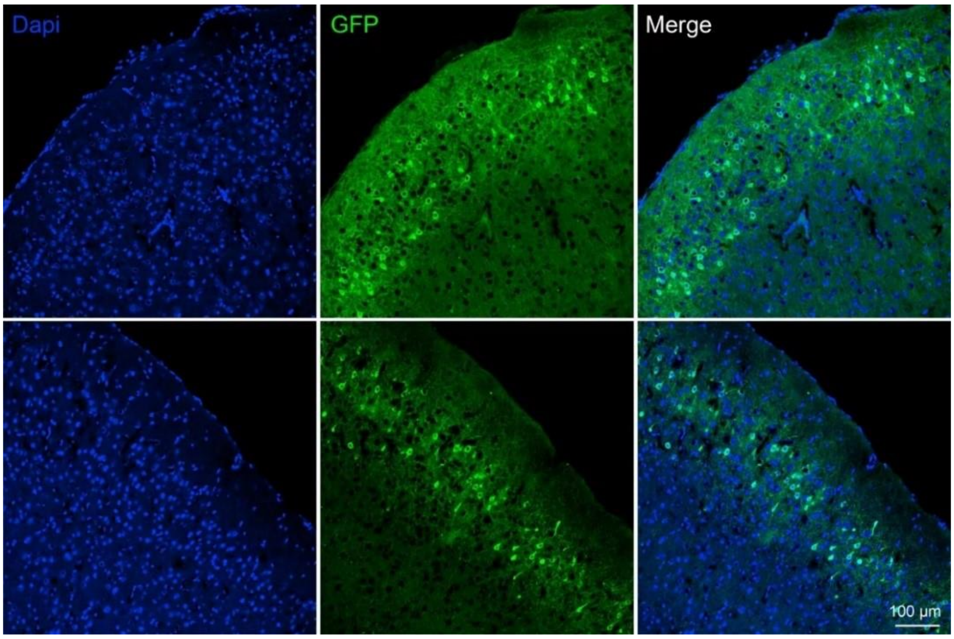

Supplementary Fig. 3 | Spatial distribution of neurons in a brain slice obtained from a Rasgrf2-2AdCre;Ai148D Mouse. Blue: DAPI. Green: GCaMP6f.

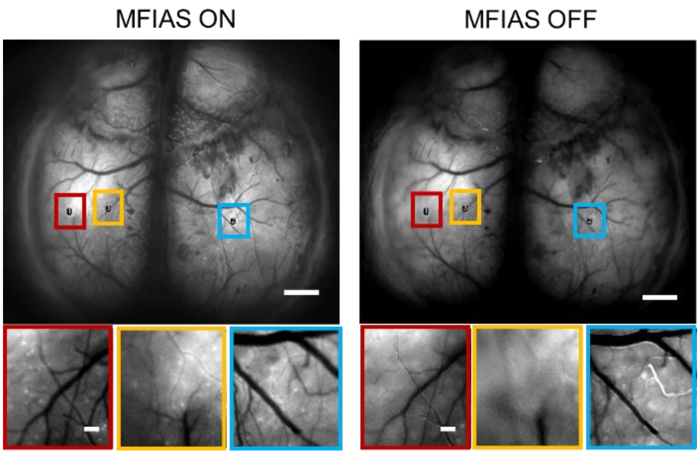

Supplementary Fig. 4 | Neural imaging of a Rasgrf2-2A-dCre; Ai148D mouse with a cleared skull. The figures depict the temporal standard deviation of the image sequence. Top, global view. Bottom, local views. Scale bars: 1 mm in global views and 100 μm in local views. Sample size, n=1 mouse .

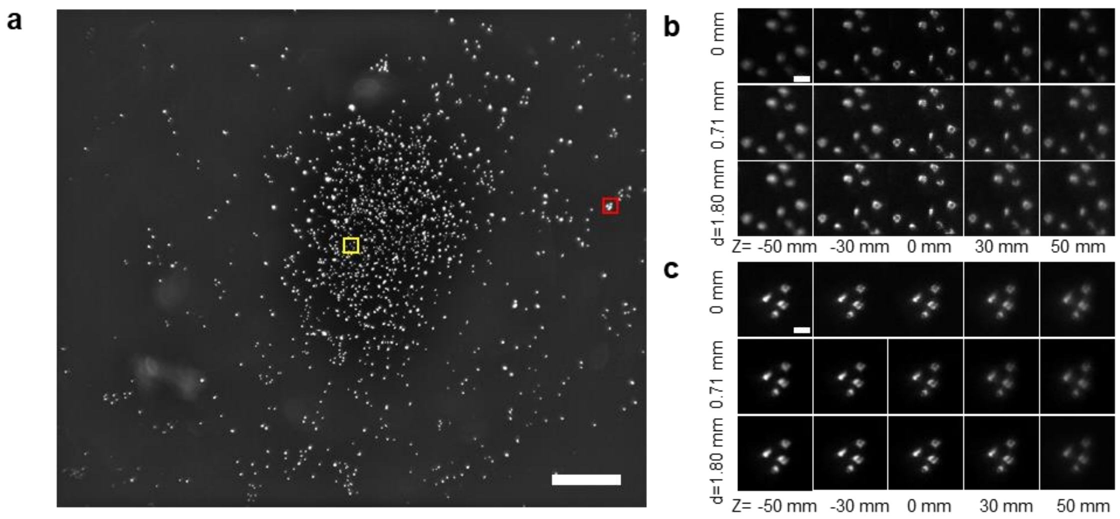

Supplementary Fig. 5 | NSC-34 cells stained with calcein-AM and observed using the MFIAS-SLR system. The sample undergoes axial stage scanning at various depths z, and the cell contours are plotted with different additional glass thicknesses d.

a, A global view of the sample. Scale bar, 1mm.

b, A local view of the yellow magnified area in (**a**). Scale bar, 20 μm.

c, A local view of the red magnified area in (**a**). Scale bar, 20 μm.

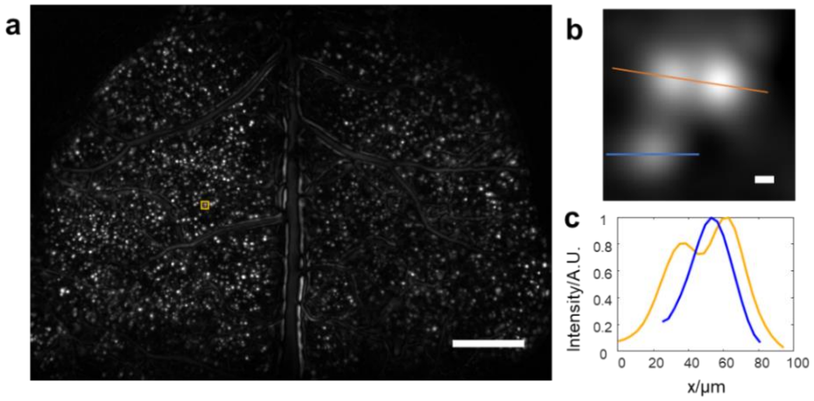

Supplementary Fig. 6 | Detection of cortex-wide neural sources using MFIAS-SLR.
a, The temporal standard derivation projection of the global view. Scale bar, 1 mm.
b, The temporal standard derivation projection of the local view. Scale bar, 10 μm.
c, The intensity profile of the light sources in (**b)**. Two light sources with a 20-μm distance can be separated due to the 18-μm FWHM of a single light source.

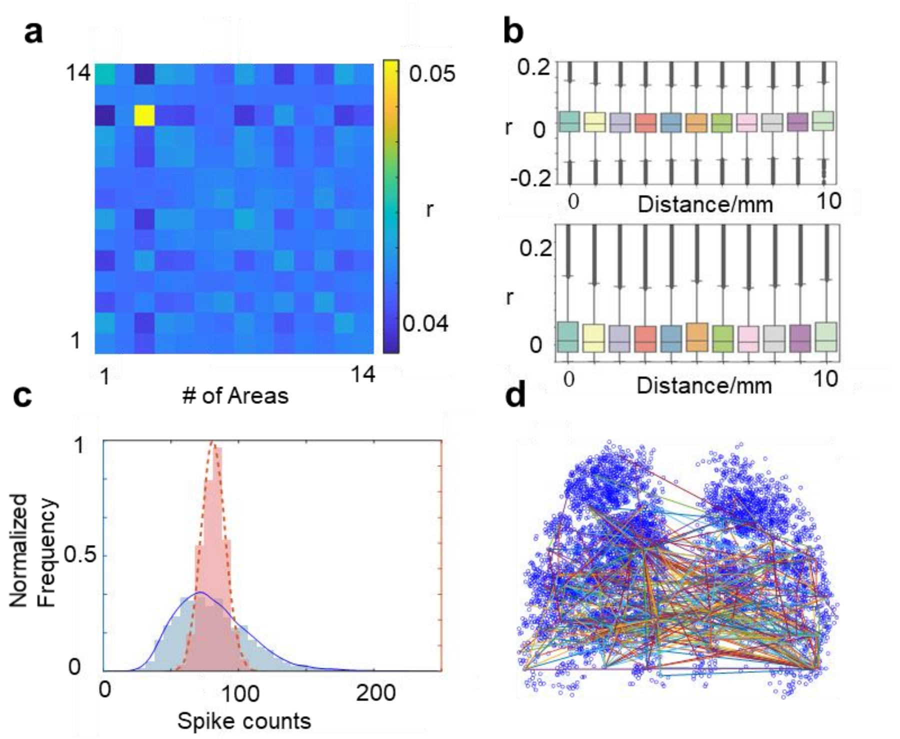

Supplementary Fig. 7 | Analysis of single neural activity obtained through MFIAS imaging.
a, Pairwise neural correlations averaged for each brain region.
b, Top, neural correlation-distance relationship. Bottom, neural correlation strength-distance relationship. Both of the correlations decrease with distance (***p < 0.001, two-sided t-tests for the Pearson correlations from biological replicates of 15,343,030 neural pairs). Central black mark: median of neural pairs (n=312,966, 1,878,888, 2,609,588, 2,647,262, 2,390,476, 2,073,680, 1,640,396, 1,056,173, 549,136, 173,333, 16,636). Bottom and top edges: 25th and 75th percentiles. Whiskers extend to extreme points excluding outliers (1.5 times above or below the interquartile range).
c, Distribution of neural spike bursts for the experimental data (red) and a randomly shuffled sequence (blue).
d, Connections formed by high neural correlations.
Brain regions: 1. Rostrolateral (RL), 2. Primary visual area (V1), 3. Posteromedial visual area (VISpor), 4. Primary somatosensory area barrel field (SSp-bfd), 5. Primary somatosensory area unassigned (S1), 6. Anterior area (Anterior), 7. Primary somatosensory area trunk (SSp-tr), 8. Primary somatosensory area upper limb (SSp-ul), 9. Primary somatosensory area lower limb (SSp-ll), 10. Anteromedial visual area (VISam), 11. Primary motor area (M1), 12. Retrosplenial area lateral agranular part (RSPagl), 13. Retrosplenial area dorsal part (RSPd), 14. Secondary motor area (M2). Sample size, n=1 mouse.

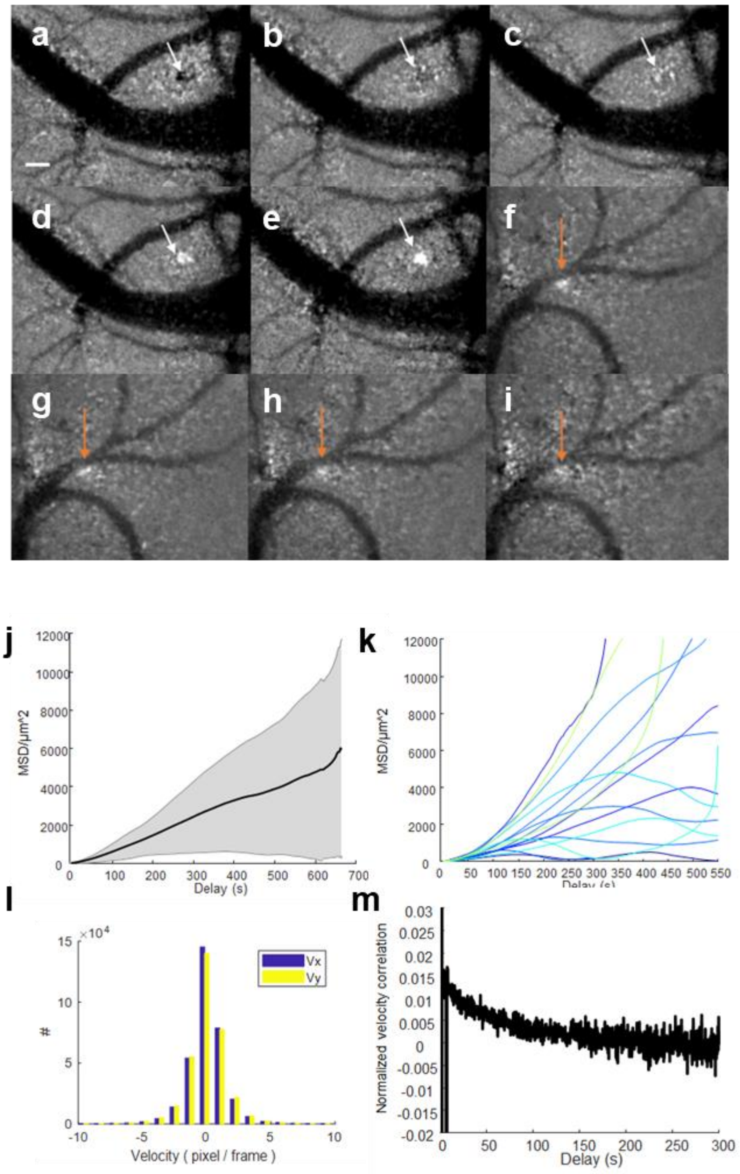

Supplementary Fig. 8 | Aggregation of neutrophil captured with the MFIAS-SLR system.
a-e, Aggregation of a group of neutrophils.
f-i, The resolution of neutrophil aggregation.
The time interval between two frames in (**a**)-(**e**) and (**f**)-(**i**) is 50 s. Scale bar, 100 μm.
j, Analysis of the 364 tracks from **Fig. 4d** reveals a linear relationship between the averaged mean squared distance and temporal delay, indicating Brownian motion within the cell group.
k, Certain neutrophils exhibit anomalous superdiffusion or subdiffusion behavior.
l, The median velocity of cells approaches zero, indicating the absence of collective directional motion.
m, Cell velocities exhibit near-zero autocorrelation.

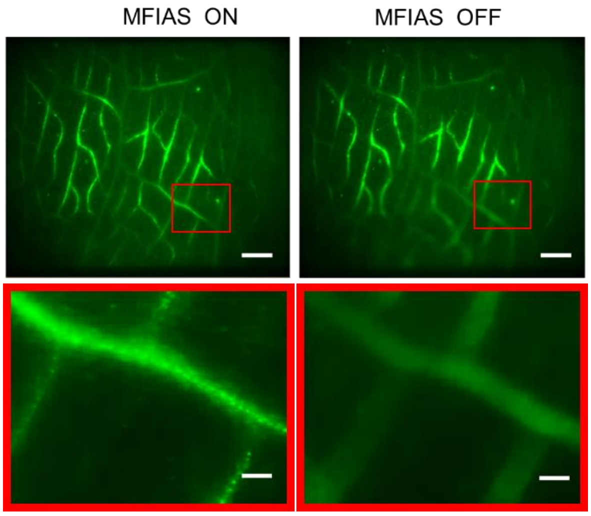

Supplementary Fig. 9 | MFIAS images of FITC solution in a leaf. The standard deviation of the image sequence over time is displayed. Images are captured at 10 frames per second. Scale bars: 1 mm in global views and 200 μm in local views. Sample size, n=1.

Supplementary Note 1

Aberration calculation and PSF measurements

In this section, we derived an analytical expression for the spherical aberration of a glass slab and conducted simulations of the point spread function (PSF) using various glass thicknesses. Our simulation results were compared with those obtained from commercial software to ensure their validity. Additionally, experimental measurements of the PSF were performed on both the RUSH and SLR systems, considering different glass thicknesses.

1.1 Analysis of spherical aberration in MFIAS

We use the angular spectrum method. For a light source at $\mathbf{F}_{\alpha}$, its virtual image is located at F. In an ideal imaging system, each optical ray from **F** has an equal optical path, so the additional optical path is calculated by $F_{\alpha}ECP'$ minus FP. It is easy to find $F_{\alpha}ECP' = PQR$. Therefore, we can calculate the optical path PQR for simplification.

$$PQR-PF = PQ + QR - PF=PQ+QM-PF-RM$$

according to the geometrical relationships

$$PQ = PL/\cos\theta_1 = nd/\cos\theta_1,$$

Since $\sin\theta_0 = n\sin\theta_1$

$$QM = QE/\sin\theta_0 = (LE\text{-}LQ)/\sin\theta_0 = \frac{(d+t)\tan\theta_0 - d\tan\theta_0}{\sin\theta_0} = \frac{(d+t)}{\cos\theta_0} - \frac{d}{n\cos\theta_1}.$$

We also have

$$PF = OF/\cos\theta_0 = (d+t)/\cos\theta_0.$$

The calculation of RM is similar, as $RM = MF_{\alpha}\cos\theta_0 = (ME - EF_{\alpha})\cos\theta_0 = (QE/\tan\theta_0 - EF_{\alpha})\cos\theta_0$. Since $QE = LE - LQ = (d+t)\tan\theta_0 - d\tan\theta_1$, $EF_{\alpha} = EF + FF_{\alpha} = t + d(1 - \tan\beta/\tan\alpha)$, and $FF_{\alpha} = d\left(1 - \frac{\tan\beta}{\tan\alpha}\right)$, we obtain

$$RM = d\left(\frac{\tan\beta}{\tan\alpha} - \frac{\tan\theta_1}{\tan\theta_0}\right)\cos\theta_0 = d\left(\frac{\cos\alpha\cos\theta_0}{\sqrt{n^2 - sin^2\,\alpha}} - \frac{\cos^2\theta_0}{\sqrt{n^2 - sin^2\,\theta_0}}\right)$$

Here, we have also used $\sin\alpha = n\sin\beta$.

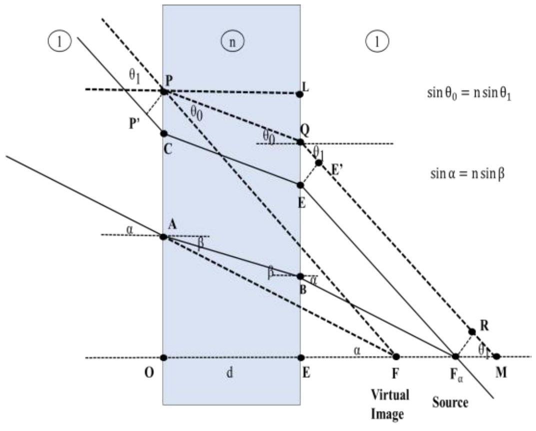

Supplementary Note Fig. 1.1: Optical rays in an imaging system with a glass slab.

Combining the above terms, we have

$$\text{PQR-PF} = = \frac{(n^2-1)d}{n\cos\theta_1} + \frac{\cos^2\theta_0}{\sqrt{n^2-\sin^2\theta_0}}d - \frac{\cos\alpha\cos\theta_0}{\sqrt{n^2-\sin^2\alpha}}d$$

$$= \sqrt{n^2-\sin^2\theta_0}\,d - \frac{\cos\alpha\cos\theta_0}{\sqrt{n^2-\sin^2\alpha}}d = nd\cos\theta_1 - \frac{\cos\alpha\cos\theta_0}{\sqrt{n^2-\sin^2\alpha}}d$$

Replacing $\theta_0 = \alpha$ and $\theta_1 = \beta$, we obtain the optical path for ray$F_\alpha BA$

$$\text{ABF}_\alpha - \text{AF} = \frac{(n^2-1)d}{n\cos\beta} = \frac{(n^2-1)d}{\sqrt{n^2-\sin^2\alpha}}$$

$$W(\theta_0,\alpha) = \text{FPQR-FPQF}_\alpha = \left(nd\cos\theta_1 - \frac{\cos\alpha\cos\theta_0}{\sqrt{n^2-\sin^2\alpha}} - \frac{(n^2-1)}{\sqrt{n^2-\sin^2\alpha}}\right)d$$

$$= \left(nd\cos\theta_1 - \frac{\cos\alpha\cos\theta_0}{n\cos\beta} - \frac{(n^2-1)}{n\cos\beta}\right)d$$

When $\alpha \to 0$, we obtain

$$W(\theta_0) = \left(n\cos\theta_1 - \frac{\cos\theta_0}{n} - \frac{(n^2-1)}{n}\right)d = \frac{1-\cos\theta_0}{n}d + n(\cos\theta_1 - 1)d = \frac{1-\sqrt{1-\sin^2\theta_0}}{n}d + \left(\sqrt{n^2-\sin^2\theta_0} - n\right)d$$

Notice that $(1-t)^\alpha = 1 - \alpha t + \frac{\alpha}{2}(\alpha-1)t^2 - \frac{\alpha}{6}(\alpha-1)(\alpha-2)t^3 + O(t^4)$, and it can be derived that$(1-t)^{1/2} = 1 - \frac{1}{2}t - \frac{1}{8}t^2 - \frac{1}{16}t^3 + O(t^4)$. Therefore, we have

$$W(\theta_0) = \frac{d}{n}\left(\frac{1}{2}\sin^2\theta_0 + \frac{1}{8}\sin^4\theta_0 + \frac{1}{16}\sin^6\theta_0\right) + nd\left(-\frac{1}{2n^2}\sin^2\theta_0 - \frac{1}{8n^4}\sin^4\theta_0 - \frac{1}{16n^6}\sin^6\theta_0 +\right) + O(d\sin^8\theta_0)$$

$$= \frac{n^2-1}{8n^3}d\sin^4\theta_0 + \frac{n^4-1}{16n^5}d\sin^6\theta_0 + O(d\sin^8\theta_0) \sim \frac{n^2-1}{8n^3}d\theta_0^4$$

Then, the spherical aberration can be estimated as $\sim d\theta_0^4/20$ for n~1.5.
For the edge ray at $\theta_0 = 0.3$ $W(\theta_0) \sim 3d/8000$

1.2. Simulations

The calculation of peak intensity with quartic aberration is addressed in the study by Leutenegger, Rao, Leitgeb, and Lasser (2006), titled "Fast focus field calculations" published in Optics Express. The authors analyse the induced quartic aberration by calculating the electric field in the image space. The resulting data are presented in a tabular format, consisting of three columns. The first column represents the x-z intensity distribution surrounding the apparent focal point. The second column corresponds to the light intensity specifically at the focal plane. The third column provides the intensity variation along the optical axis.

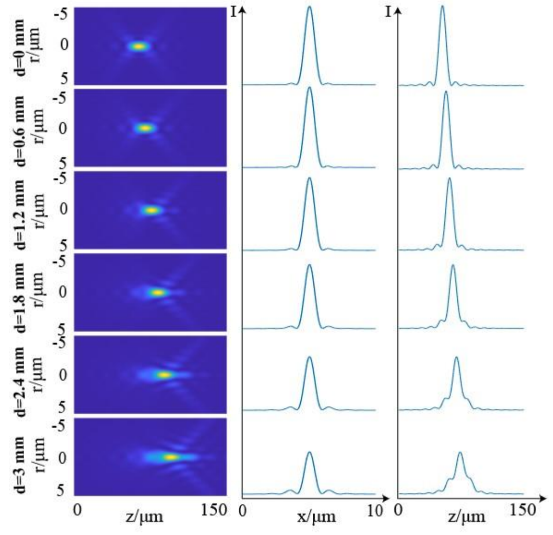

Supplementary Note Fig. 1.2 | Simulated light intensity at various glass thicknesses. The light intensity in the xz plane, along the lateral x-axis and the axial z-axis, is plotted in three columns. Each row of the figure matrix corresponds to one glass thickness ranging from 0 to 3 mm. The simulation parameters used include a numerical aperture of 0.3, a wavelength of 0.5 μm, and circular polarization. The results demonstrate a decrease in both peak intensity and axial resolution with increasing glass thickness.

In addition, we compiled the intensities of the various glass thicknesses and calculated the full width at half maximum (FWHM) for each thickness in **Extended Data Fig. 4**.

Based on our findings, it can be concluded that for glass slabs with a thickness smaller than 2 mm, both the axial and lateral resolution exhibit gradual changes. However, when the thickness exceeds 3 mm, a notable decrease in axial resolution is observed.

1.3. Relay systems

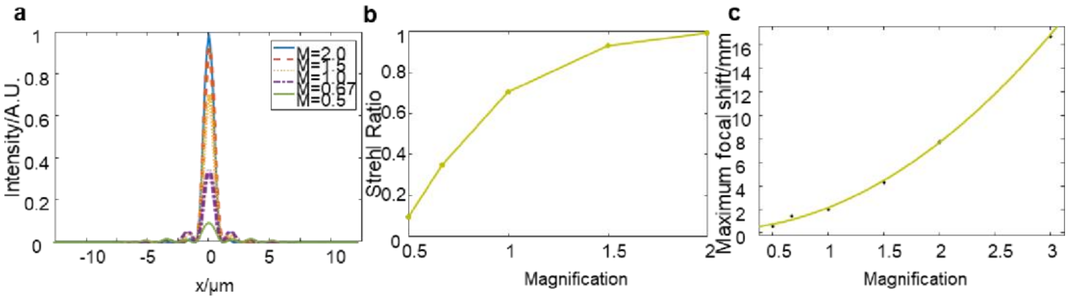

Supplementary Note Fig. 1.3 | Cover glass analysis in different intermediate image spaces. To evaluate the optical performance of MFIAS, we compare the system with varying magnification factors M with a reference case of 0.3 NA and 2 mm cover glass. **a**, The light distribution is shown when we maintain equal focus shifts and vary the magnification. **b**, The Strehl ratio is plotted against magnification. **c**, If we keep the Strehl ratio at 0.7, as in the case of 0.3 NA, we also plot the maximum allowed focus shift under different magnifications. It is observed that the focus shift increases quadratically with magnification, as predicted by theory.

1.3. Experimental validation

1.3.1 RUSH system

Our developed RUSH microscopy enables the detection of a centimeter-scale field of view (FOV) at micrometer resolution through the parallel acquisition of 35 cameras, facilitating the assessment of MFIAS performance on the RUSH platform. Optimization of the point spread functions was carried out for the central 31 cameras.

Data acquisition involved the utilization of 0.5-μm fluorescent beads as the test sample. By placing glass at various thicknesses, we scanned the axial position of the stage at 2.5 μm intervals to move the sample. In total, we captured 40 z-stacks for 35 cameras across all 10 glass thicknesses. However, only the data from the central 31 cameras were subjected to analysis. Therefore, all statistical analyses were performed on a total of 12,400 images, each captured at a resolution of 2560*2160 and 16-bit depth. The illumination was uniformly distributed throughout the entire FOV. To determine the focal depth of each stack, we employed a method inspired by two-photon imaging, summing up the squared intensity of each pixel and obtaining the focal depth from the stack with the maximum intensity.

Initially, we imported the image stack into MATLAB and divided the entire image into small patches containing beads. Subsequently, we used the image located 50 μm away as the background image, where the bead intensity dropped 5% below the peak intensity. We subtracted this background image from the original images. The connected components of the image were then identified using the *imextendedmax* and *regionprops* functions on the z-averaged images. We applied filters to remove bead aggregations based on the area and intensity of the beads. Once the bead positions were determined, we divided the original images into small 40*40*40 image stacks.

For the curve fitting process, we initially identified the stack with the highest light intensity and performed Gaussian fitting on all the beads. To further eliminate aggregates and noise, we excluded the 1% largest and smallest FWHM beads.

We generated point spread function plots for five beads at different glass thicknesses. Additionally, we conducted statistical analyses on the lateral resolution using fluorescent beads from the central camera, with

an average of 50 beads per glass thickness. The maximum measured axial resolution for our system was 18 μm, slightly larger than the theoretical resolution of 10 μm.

We plotted the point spread function for five beads at different glass thicknesses. We further obtained statistics on the lateral resolution from fluorescent beads from the central view (50 beads for each glass thickness on average.). The maximum axial resolution measured for our system is 18 μm, which is larger than the 10 μm resolution in theory but much smaller than the induced focal shift.

Please note that the RUSH system incorporates a 1-mm glass between the objective and the specimen, with the first glass inducing the most significant aberration.

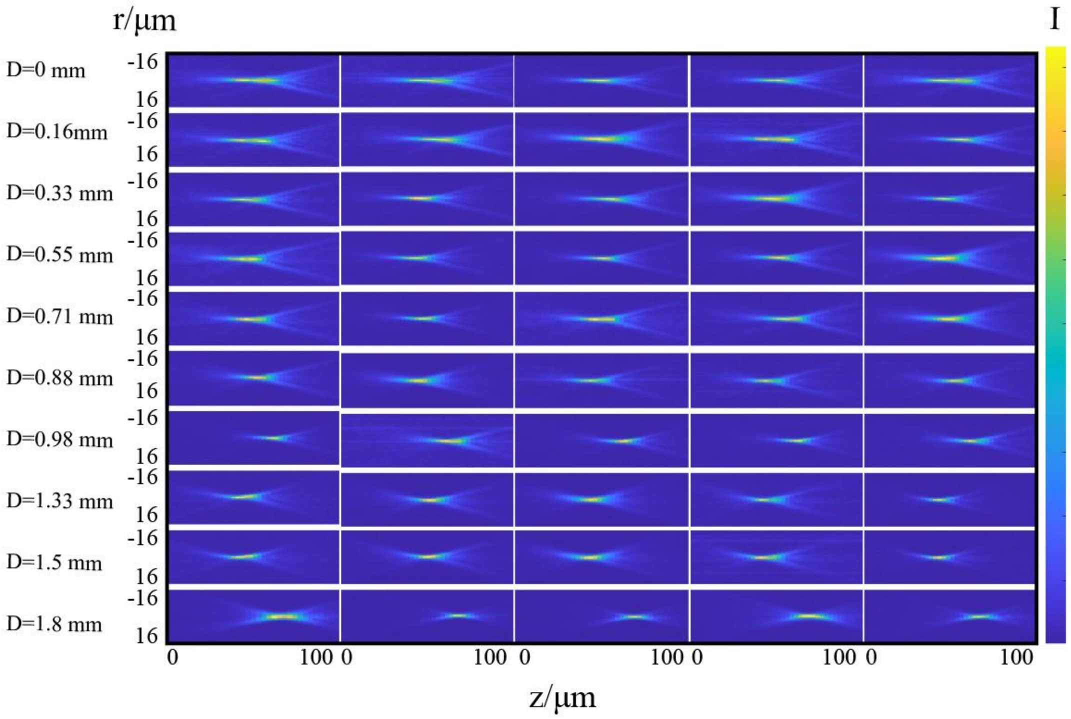

Supplementary Note Fig. 1.4 | xz intensity profiles captured by the central camera using various thicknesses of glasses.

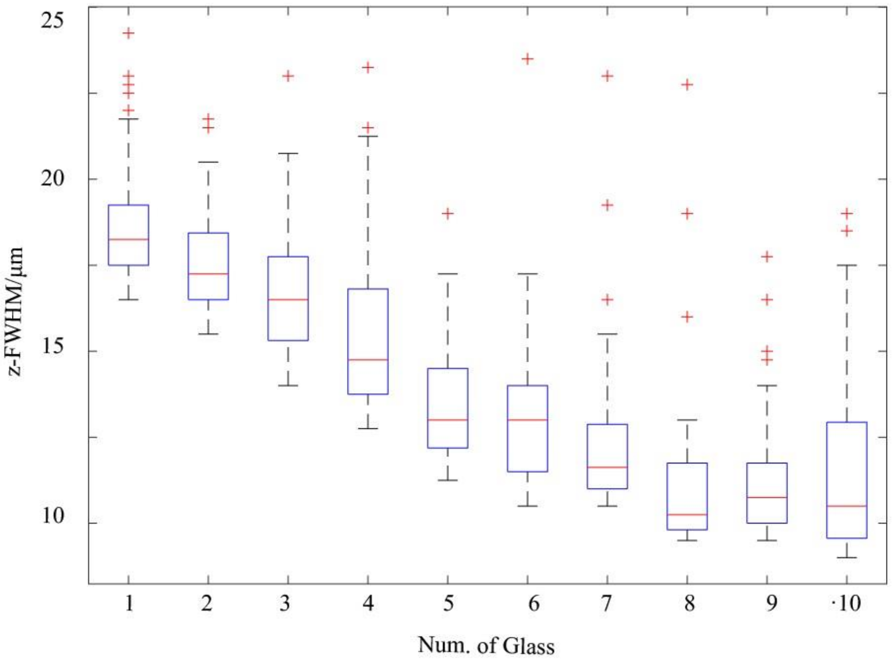

Supplementary Note Fig. 1.5 | Axial FWHMs for fluorescent beads with varying glass thickness at the central camera. Bead number, n=76, 27, 31, 41, 41, 22, 44, 43, 43, 139 for each thickness. Central black mark: median of the biological replicates. Bottom and top edges: 25th and 75th percentiles. Whiskers extend to extreme points excluding outliers (1.5 times above or below the interquartile range).

1.3.2 SLR System

We perform a similar procedure as in the RUSH. We interpolate on the whole FOV, as shown in **Supplementary Note Fig. 1.6**. It is shown that the edge of the FOV has aberration resulting from the system coma.

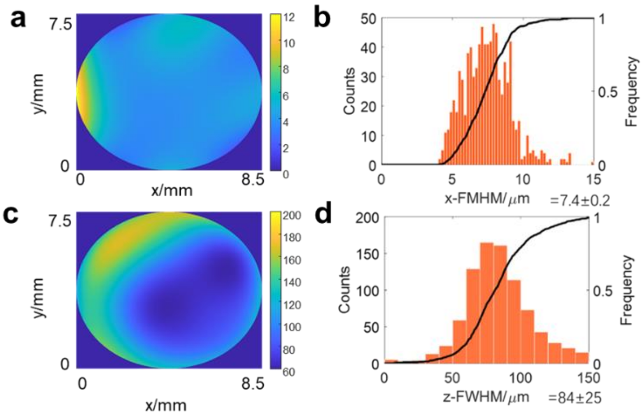

Supplementary Note Fig. 1.6 | Distribution of the lateral and axial resolutions of the SLR system. a, Spatial distribution of lateral resolution. **b**, The histogram of lateral resolution. **c**, Spatial distribution of axial resolution. **d**, The histogram of axial resolution. The FWHMs are presented as the mean±s.d.

We captured approximately 900 beads for 10 thicknesses and calculated the lateral and axial resolution for each bead. The lateral resolution (mean±s.d.) are 7.5±1.6 μm, 7.5±1.5 μm, 7.7±1.6 μm, and 7.5±2.0 μm at glass thicknesses of 0, 0.55 μm, 0.98 μm, and 1.8 μm, respectively. The axial resolution (mean±s.d.) are 91±29 μm, 88±27 μm, 88±23 μm, and 86±20 μm at glass thicknesses of 0, 0.55 μm, 0.98 μm, and 1.8 μm, respectively.

1.4. Discussion on the higher order aberrations

RUSH is telecentric in the sample space, so all the light is telecentric for our RUSH system. For customized SLR systems, the principle optical rays from the edge FOVs are not telecentric. Here, we list the higher order aberration induced by the tile incident:

$$W_{\{22\}} = \frac{n^2 - 1}{4n^3}\alpha^2 d(NA)^2$$
$$W_{\{31\}} = -\frac{n^2 - 1}{2n^3}\alpha d(NA)^3$$

We scan the axial of the beads to obtain the α of the rays. In the corner of the FOV, the lateral movement of beads is approximately 10 μm, corresponding to 500 μm focal movement, so $\alpha < 0.01$ in a circular FOV with diameter $d < 8$ mm. We noticed that the linear coma is less than $\lambda/4$ for a 1.3-mm glass thickness and much smaller than the system coma, and we ignore the induced field-dependent aberrations in the nontelecentric system.

Supplementary Note 2 | Depth extension

As previously discussed, the axial range of MFIAS can be extended with the help of relay systems. In this section, we implement these systems and experimentally measure the point spread functions with high DOF. We have made three additional components for large DOF scanning, small step scanning, and continuous scanning in **Extended Data Fig. 1**. The large z-scale scanning results are shown in **Supplementary Note Fig. 2.1**, Small step scanning results are shown in **Supplementary Note Fig. 2.2**. Continuous scanning results are shown in **Supplementary Note Fig. 2.3**. In **Supplementary Note Fig. 2.4**, we show the application of MFIAS in high NA systems.

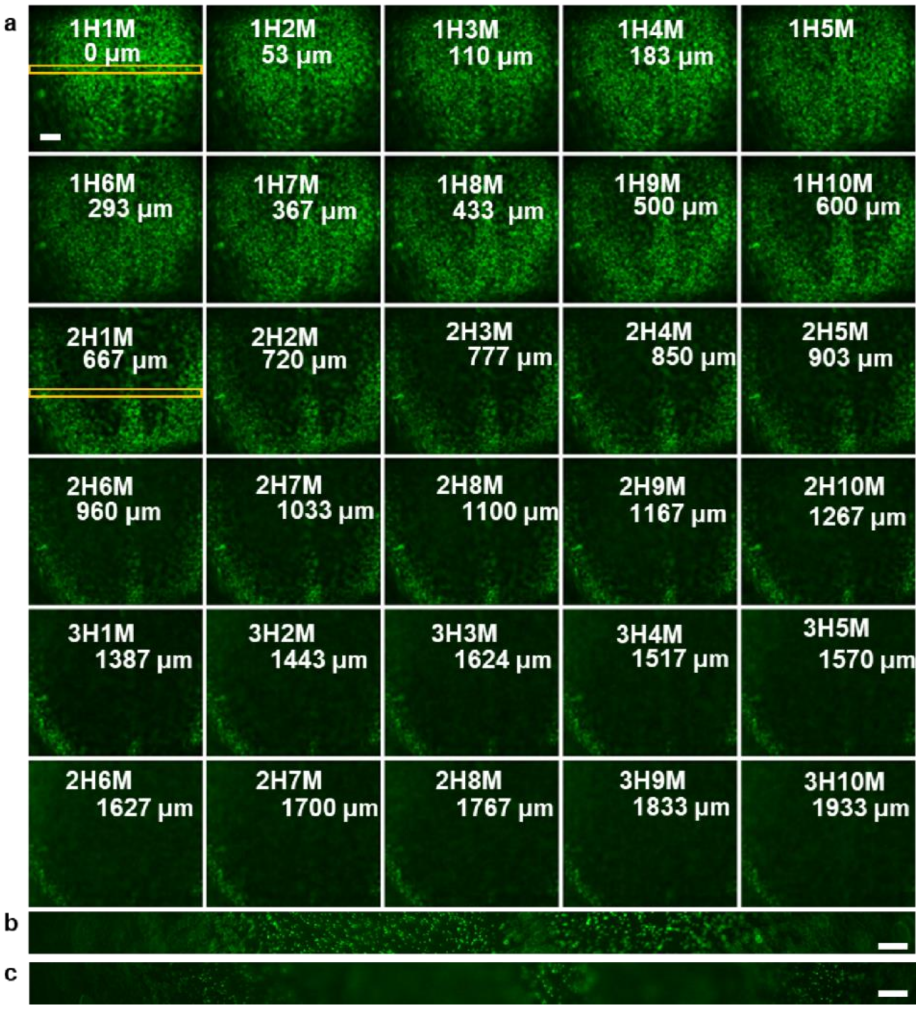

Supplementary Note Fig. 2.1 | Large-scale z-scan in MFIAS. A z-scan of the fluorescent beads in a mouse brain model using the "hour" and "minute" disk from 0 to ~2 mm. **a**, A continuous z-scan. **b-c**, Two close-ups form **(a)**. Scales bar, 1 mm in **(a)** and 100 µm in (**b)** and (**c)**

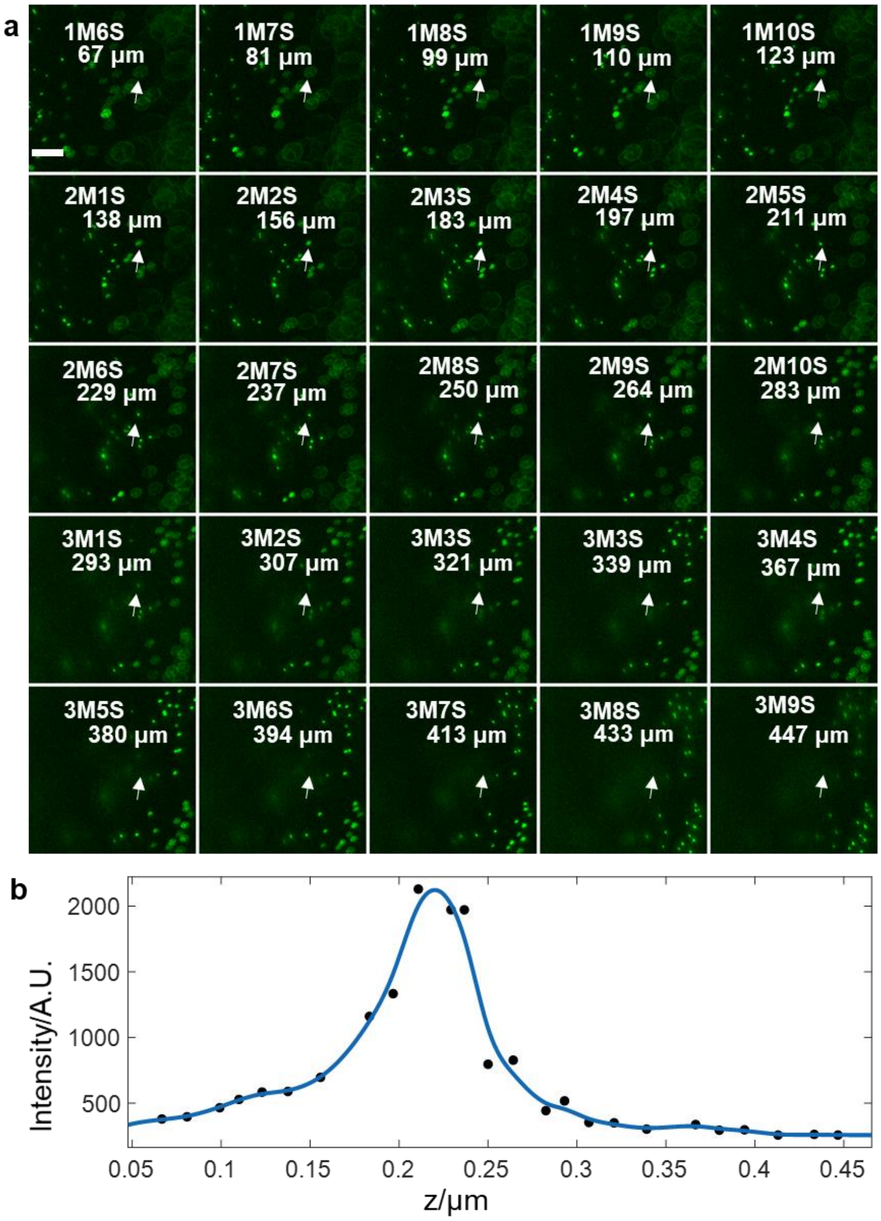

Supplementary Note Fig. 2.2 | A scan of a mouse brain model, taken with the "minute" and "second" disks. a, 1M1S refers to the first cover glass in the "minute" disk and the first disk in the "second" disk, located at a depth of 0 μm. **b,** The lateral intensity profile of the bead in **a**. Scale bar, 1 mm.

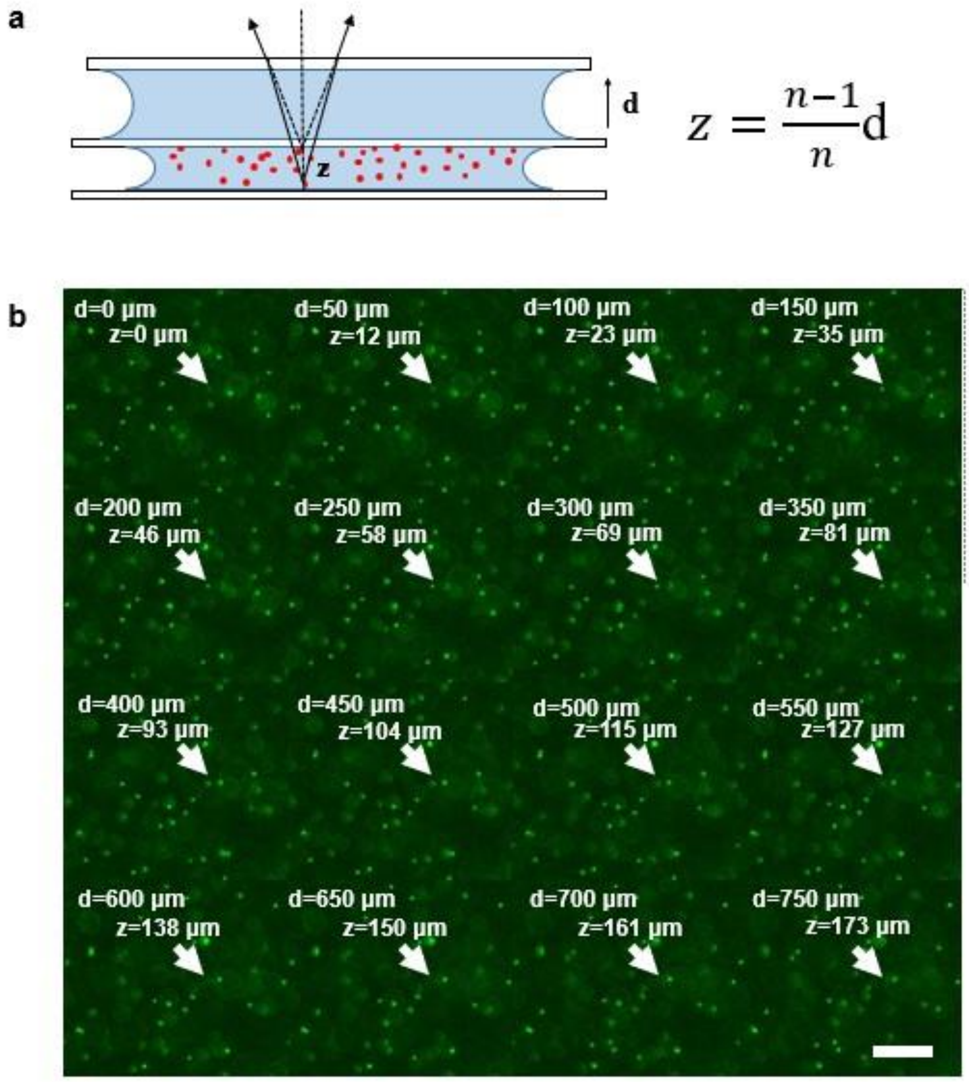

Supplementary Note Fig. 2.3 | A z-scan of 3-µm fluorescent beads conducted by the liquid disk. a, The schematic diagram of the continuous scan. **b**, Result of the continuous scan. "d" represents the position of the upper cover glass, "n" is the refractive index of the media between the two cover glasses, and "z" is the apparent depth of the focal plane in the specimen. Scale bar, 100 µm.

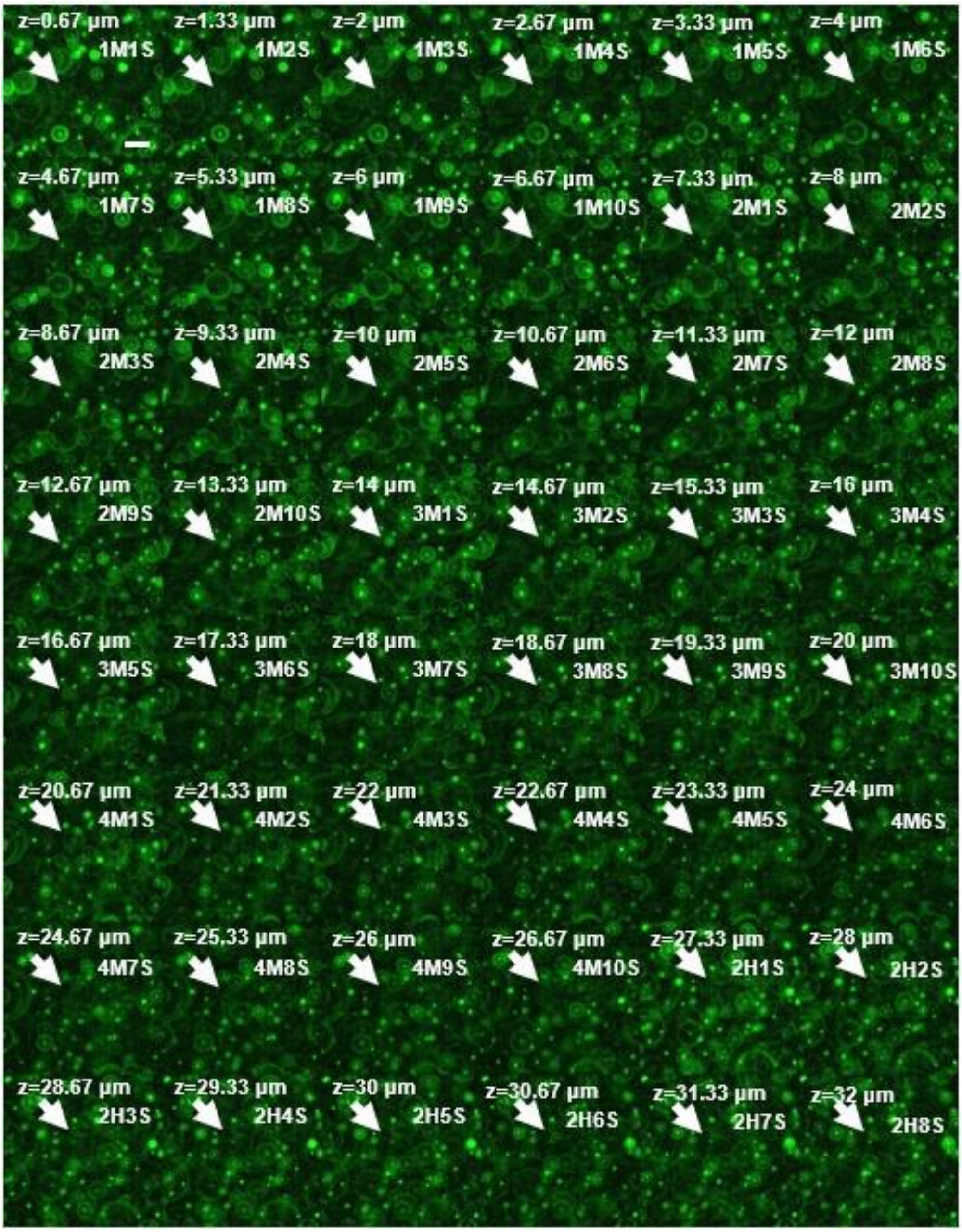

Supplementary Note Fig. 2.4 | A z-scan of 200-nm fluorescent beads using the high-NA MFIAS system. The 1M1S refers to the first cover glass in the hour disk, the first cover glass in the minute disk, and the first disk in the second disk, located at a depth of 0.67 µm. The 2H8S refers to the second cover glass in the hour disk, the first cover glass in the minute disk, and the eighth disk in the second disk, located at a depth of 32 µm. Scale bar, 10 µm.

Supplementary Note 3 | Comparison of 1p and 2p Neural Detections.

3.1. Validation of neural resolution with a hybrid 1p and 2p system

In a previous study (Zhang, Y., Zhang, G., Han, X. et al., 2023), wide-field detection of single neural activity was compared with a two-photon microscope. The experimental setups and animal models were consistent with our current configuration, except for the use of a 10X/0.3 objective as the secondary imaging objective. We have developed a system to validate the capability of our mesoscopic imaging setup in capturing single neural activity. The system comprises a titanium-sapphire laser system (MaiTai HP, Spectra-Physics) for twophoton excitation (920 nm central wavelength, pulse width <100 fs, 80 MHz repetition rate). Beam scanning is achieved through a resonant scanner (8315K/CRS8K, Cambridge Technology), while a high-NA water immersion objective (25×/1.05 NA, XLPLN25XWMP2, Olympus) and a piezo actuator (P-725, Physik Instrumente) enable precise focusing and axial scanning, respectively. To ensure compatibility between excitation and widefield detection, an iris is employed to reduce the beam size, and a longpass dichroic mirror (DMLP505L, Thorlabs) is used to separate fluorescence signals from the laser beam.

For widefield excitation, a longpass dichroic directs blue LED light (M470L4-C1 and MF475-35, Thorlabs) toward the objective. A nonpolarizing plate beam splitter (BSW27, Thorlabs) separates fluorescent signals, which are then detected by a photomultiplier tube (PMT, PMT1001, Thorlabs) and a camera (Zyla 4.2, Andor). Fluorescence filters (MF525-39, Thorlabs; ET510/80M, Chroma) are employed to block the laser and widefield excitation beams. Additionally, a linear galvo serves as an optical shutter to deflect widefield fluorescent photons during LED illumination, and an electro-optic modulator (EOM, 350-80LA-02, Conoptics) is blocked during widefield imaging to prevent crosstalk and safeguard the PMT. The LED operates in trigger mode with reduced duration to prevent PMT overexposure. Finally, a pair of 50 mm/100 mm SLR lenses relays the wide-field signals to the camera, following the configuration of our MFIAS system.

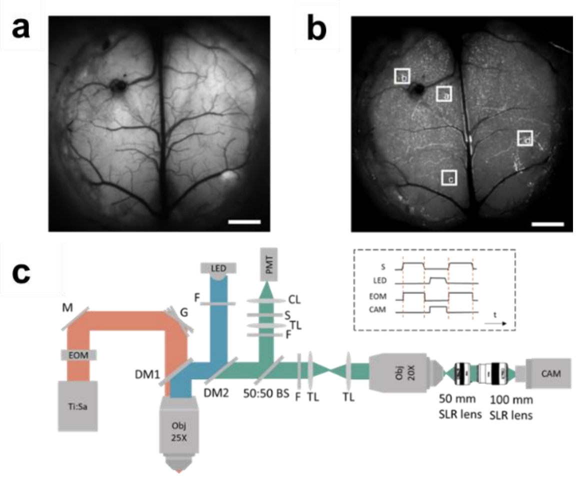

Supplementary Note Fig. 3.1 | The comparison neural signals of single-photon and two-photon microscopy. a, A single-photon image captured by the SLRM system is shown. While active neurons can be identified through background subtraction, silent neurons remain undetected. . Scale bar, 1 mm. **b**, The standard deviation of the image stacks, showing the presence of active neurons. Further comparison of neural activity in four subfields of view (FOV) is presented in the subsequent supplementary note figure. Scale bar, 1 mm. **c**, Schematic diagram illustrating simultaneous 1-p and 2-p imaging.

In our study, simultaneous imaging was conducted in four distinct subregions, as depicted in **Supplementary Note Fig. 3.1**. Initially, we presented the standard deviation of the 1p and 2p sequences. The CNMF-E algorithm was employed to extract the contours of identified neurons in the 1p sequence. In **Supplementary Note Fig. 3.2**, (**a3**)-(**d3**) demonstrates the algorithm's accurate detection of neuron contours. Additionally, we overlaid the 1p contours onto the 2p images, revealing that the majority of neurons detected by the 1p microscope were individual neurons. (**a5**)-(**d5**) illustrates the close correspondence between the neural traces of 1p (blue) and 2p (red). Notably, the performance of the 1p results is reliant on the signal-to-noise ratio of the detected neurons.

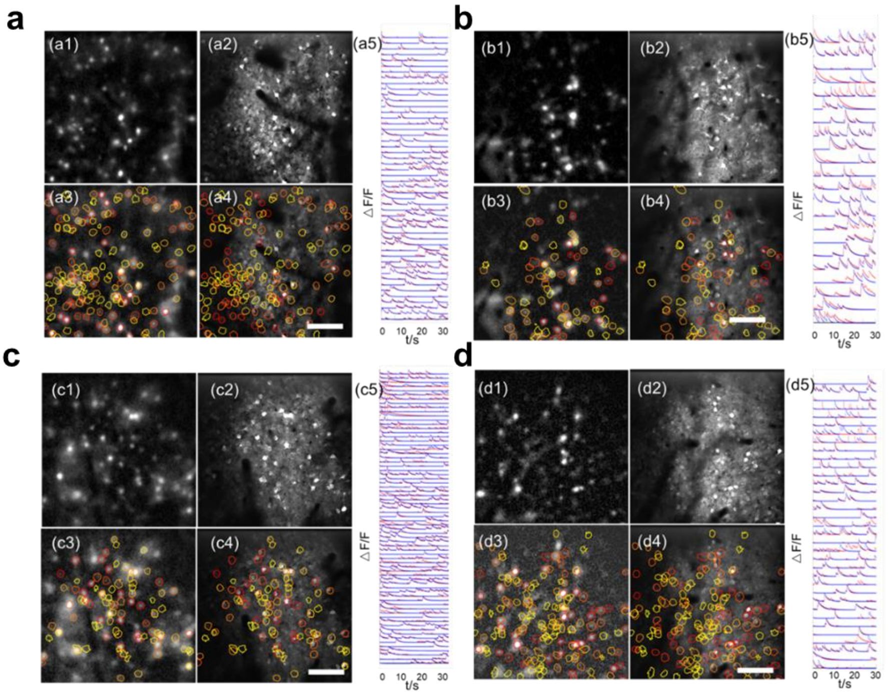

Supplementary Note Fig. 3.2 | Close-ups of comparison between singlepPhoton and two-photon results. a1, b1, c1, and **d1,** Standard deviation images of single-photon image sets. Scale bar, 100 μm. **a2, b2, c2, and d2,** Standard deviation images of two-photon image sets. The CNMF-E algorithm was applied to both types of image sequences. **a3, b3, c3,** and **d3**, The detected neural contours in the single-photon image sequence overlaid on the corresponding single-photon images, demonstrating the successful detection of a majority of neurons in wide-field detection. **a4, b4, c4,** and **d4**, The detected neural contours in the singlephoton image sequence overlaid on the two-photon images, highlighting the strong correspondence between neurons detected in single-photon microscopy and those in two-photon microscopy. **a5, b5, c5,** and **d5**, Wellmatched neural traces obtained using the two different imaging modalities, which is attributed to the sparse neural labelling technique employed in the Rasgrf2-2A-dCre;Ai148D mouse model.

In our study, we employed a semiautomatic algorithm to match the neural signal between 1p and 2p images. Initially, we utilized the CNMF-E algorithm with a neural diameter of 13 pixels to identify the neurons. The minimum local correlation threshold for seed pixels was set at 0.8. Subsequently, we employed this mask to match the neurons in the maximum projection of 2p images and locate the corresponding light source in the 1D signal. The 2p signals were obtained by subtracting a minimal temporal projection from the original 2p images. To extract the signal from the 1p images, we employed a ring-shaped background estimation technique. This involved applying the same background subtraction procedure and subtracting a blurred image using a spatial Gaussian kernel with σ=20.

Our analysis revealed that the 1p results exhibited a consistent correlation of approximately 0.8 with the 2p results across all six fields of view (FOVs).

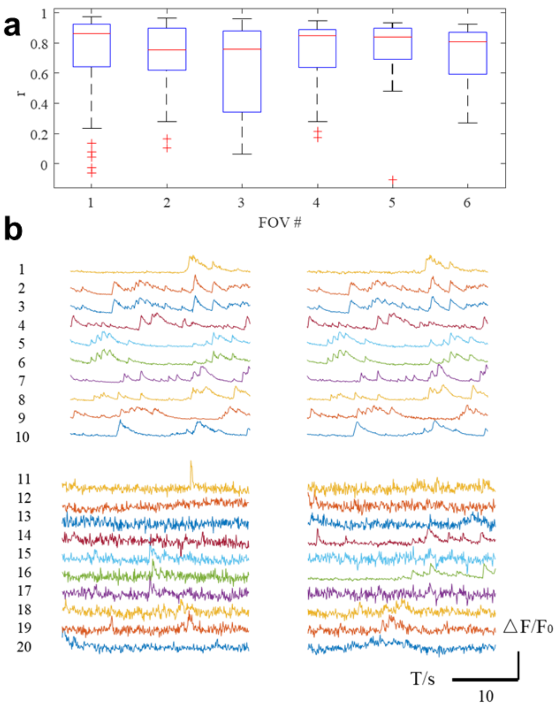

Supplementary Note Fig. 3.3 | Statistics of the 1p and 2p neural signal results. a, 1p-2p correlation of biological replicates of 279 neurons from 6 different brain regions in a mouse brain. Central black mark: median of the biological replicates. Bottom and top edges: 25th and 75th percentiles. Whiskers extend to extreme points excluding outliers (1.5 times above or below the interquartile range). **b,** Ten measurements with the largest correlations and ren measurements with the smallest correlations.

To estimate the background noise level, we computed the median standard deviation of the background pixels. Specifically, we sampled 30 regions from the background and obtained a value of σ=860±256. The signal-to-noise ratio (SNR) of the trace was determined by dividing the maximum intensity by the standard deviation. We observed a correlation value of 0.8 when the peak-signal-to-noise ratio (PSNR) exceeded 1.5.

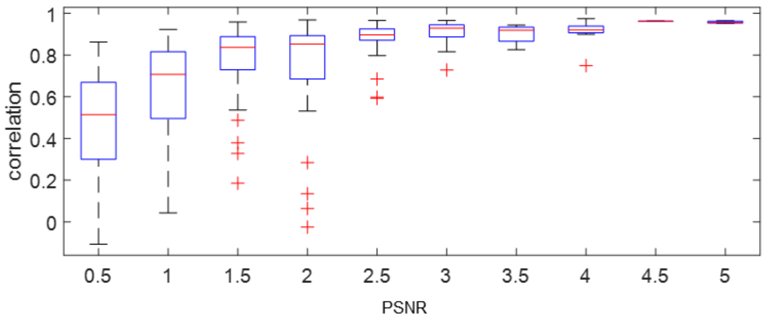

Supplementary Note Fig. 3.4 | Boxplot of 1p-2p correlation and PSNR. The correlation surpasses 0.8 when the peak signal exceeds the background noise level by a factor of 1.5. The 1p-2p correlations were taken from 279 neurons. Central black mark: median of the biological replicate. Bottom and top edges: 25th and 75th percentiles. Whiskers extend to extreme points excluding outliers (1.5 times above or below the interquartile range)

3.2 Conditions for accurate neural signal extraction

To assess the limitations of a 1p microscope in achieving high correlation with 2p, we examined a dataset comprising both directly acquired data and data adapted from our previous work obtained from depths ranging from 130-230 μm. In addition to the peak-signal-to-noise ratio (PSNR), we identified sparsity as a crucial factor for accurate signal extraction. For instance, our findings indicate that correct signal extraction can be achieved when the distance between neurons exceeds 10 μm at depths below 200 μm. However, contamination from nearby neurons can occur when the distance is only 8 μm at a depth of 130 μm.

According to the measurements in **Supplementary Fig. 5**, the diameter of the signal emitted by each neuron is approximately 20 μm in the superficial cortex. To ensure a minimum separation of 20 μm between individual neurons, the neuronal density in the brain should be significantly lower than 2,500 neurons/mm².

3.3 Comparison of 1p and 2p correlations

We present the correlation distribution of 7,475 neural pairs obtained from both the 1p and 2p results. **Supplementary Note Fig. 3.5a** illustrates that correlations derived from 1p and 2p data exhibit similar strengths and patterns. In five out of six field-of-views (FOVs), the median neural correlations from 1p are not higher than those from 2p (**Supplementary Note Fig. 3.5b**). Additionally, the correlation across all FOVs is not higher in 1p than in 2p (**Supplementary Note Fig. 3.5c**).

To assess the error in correlations between 1p and 2p, we plot the distributions of absolute neural correlations from both methods. We observe an approximate 5% difference in the median correlation. Furthermore, over half of the neural correlations from 1p exhibit an error of less than 0.05 when compared to 2p results, suggesting that 1p data can be utilized to infer neural correlations.

Theoretically, raw 1P calcium traces from each source should demonstrate a higher correlation compared to high-resolution 2P recordings due to cross-contamination from neighboring neurons and defocused dendritic backgrounds. However, the demixing algorithm effectively separates signals from different neurons and eliminates background signals. For instance, the CNMF-E algorithm addresses cross-contamination of neighboring neurons through temporal correlation in the spatial update step, as demonstrated in

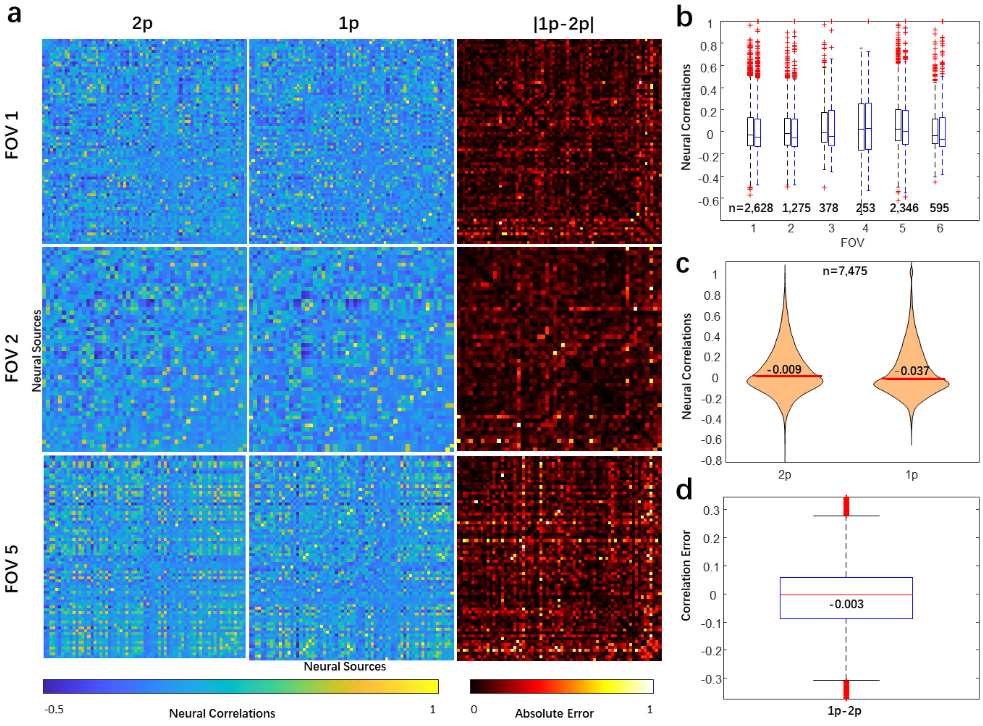

Supplementary Note Fig. 3.5 | Comparison between 1p and 2p correlations. a, The correlations of neurons in FOVs 1, 2, and 5 calculated from both 1p and 2p results in the first two columns and their difference in the third column. The autocorrelation is set to zero for better visualization. **b**, The distribution of correlations for all six FOVs. Neuron numbers, 73,51,28,23,69,35. Black bars: two-photon correlations. Blue bars: single-photon correlation. Central mark: median of the biological replicates, r=-0.026, -0.017, -0.01, 0.024, -0.036 for two-photon, and r=-0.048, -0.053, -0.043, 0.028, -0.067. Bottom and top edges: 25th and 75th percentiles. Whiskers extend to extreme points excluding outliers (1.5 times above or below the interquartile range). **c**, A violin plot of the distributions of pairwise correlations and the correlation strengths from all FOVs, showing that the correlation across all FOVs is not higher in 1p than in 2p (***p=9e-7, onesided paired t-test). **d**, Box plot of the correlation errors. Central black mark: median of the biological replicates. Bottom and top edges: 25th and 75th percentiles. Whiskers extend to extreme points excluding outliers (1.5 times above or below the interquartile range). In (**c**) and (**d**), statistics are made from the biological replicate of 279 neurons.

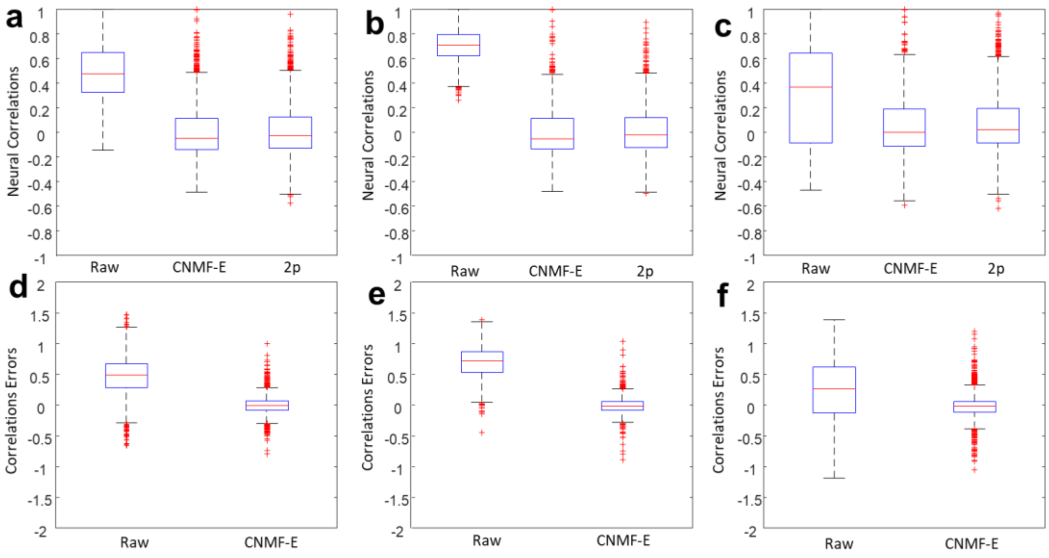

Supplementary Note Fig. 3.6 | Comparison of neural correlations obtained from raw intensity measurements and those derived using the CNMF-E algorithm. a –c, Distributions of neural correlations for FOVs 1, 2, and 5, respectively. **d-f**, Distributions of correlation errors for the same FOVs. Central black mark: median. Bottom and top edges: 25th and 75th percentiles. Whiskers extend to extreme points excluding outliers (1.5 times above or below the interquartile range). Statistics are made from the biological replicate of 73,51,28,23,69,35 neurons in (**a**)-(**f**), respectively.

Supplementary Note 4 | Speed of surface detection and image acquisition

4.1 Detection speed

In this section, we present the process of single-frame acquisition and the time for surface detection. Our findings demonstrate that single-frame acquisition algorithms can detect surfaces with subsecond temporal resolution, enabling the detection of fast shape variations in the sample.

4.1.1. Setup of the evaluation system

To evaluate our algorithm, we utilized a tilted cover glass sample with a tilt angle of approximately 5° and a height difference of approximately 400 μm. The parameters of our computational platform are summarized in the table.

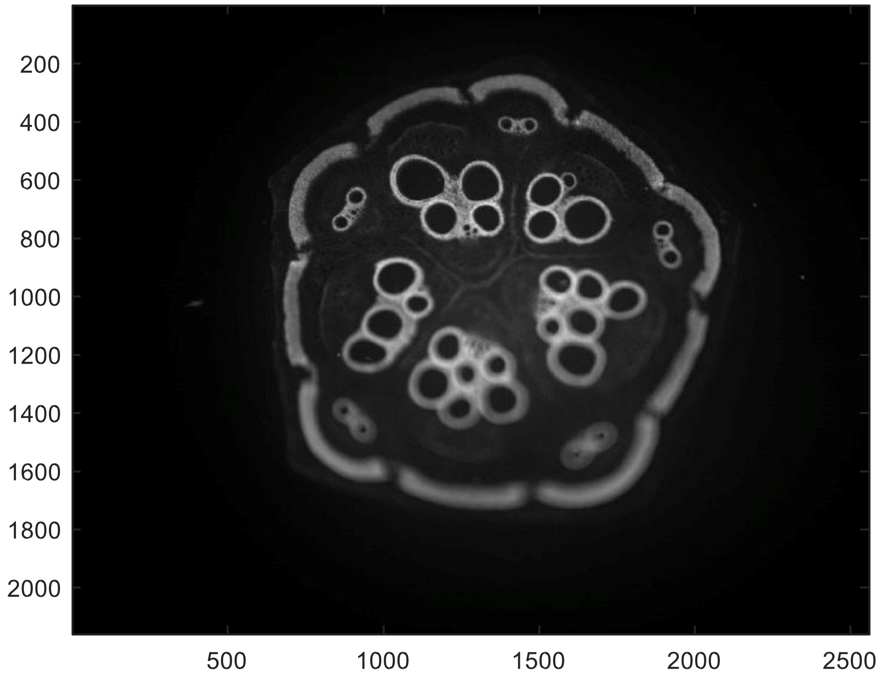

Supplementary Note Fig. 4.1 | A tilt slice sample when MFIAS is OFF. The units for the x- and y-axes are pixels.

Table 4.1: Platform parameters for our evaluation

CPU	Intel(R) Core (TM) i5-6400 CPU @ 2.70 GHz 2.70 GHz
Operation system	Windows 10 Professional
RAM	64.0 GB
Hard Disk	Intel SSD SC2KW010T8, 1TB
GPU	NVIDIA GeForce GT 710 2GB
Software	MATLAB R2020a

4.1.2. Data acquisition (0.1 s)

The DMD memory stores and activates illumination patterns during the surface detection stage. By illuminating different positions with varying glass thicknesses, the correct combination generates image patches with high-contrast edges. **Supplementary Note Fig. 4.2** illustrates the illumination pattern, where different colors represent different glass thicknesses. For example, the yellow patches correspond to the tenth glass thickness, indicating a depth of approximately 600 μm from the focal plane. To achieve singleshot data acquisition, the illumination sequence on the image plane is converted into the DMD illumination pattern using a precalibrated transformation matrix. The data acquisition process was completed within 100 ms in our experiment.

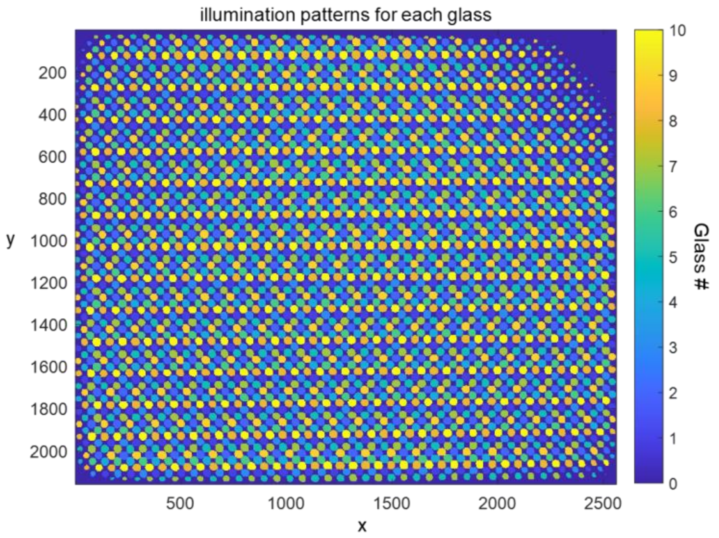

Supplementary Note Fig. 4.2 | The Illumination pattern used for single-frame detection. The color represents the depth when the illumination is set on. The units for the x- and y-axes are pixels.

4.1.3. In-focal Patches Detection (0.820±0.005 s)
To assess the limits of the focal detection algorithm, we employ a straightforward algorithm consisting of the following steps:

1. Laplacian Intensity Detection: The algorithm initially calculates the Laplacian of the original image to detect sharp edges, enabling the identification of regions with significant intensity changes or boundaries.
2. Gaussian Filtering: The detected sharp edges undergo Gaussian filtering to obtain the regional average for each patch.
3. Maximum Patch Selection: The algorithm sequentially selects the patch with the highest average intensity, records its central coordinates, and removes it from the original image.
4. Iteration and Patch Removal: Steps 2 and 3 are iterated 30 times, with each iteration identifying and removing patches with the highest average intensity.
5. Curve Fitting: Following the 30 iterations, the remaining clear patches undergo a curve fitting process. This step utilizes prior surface information, such as linearity or smoothness, to accurately fit the surface shape.
6. Illumination Generation: Based on the reconstructed surface shape, illumination patterns are generated for subsequent surface detection or analysis.

The experimental results reveal that the entire surface detection algorithm has an average execution time of 0.820±0.005 seconds (n=10). It is noteworthy that the most time-consuming aspect of the algorithm is the computation of the Laplacian of the image.

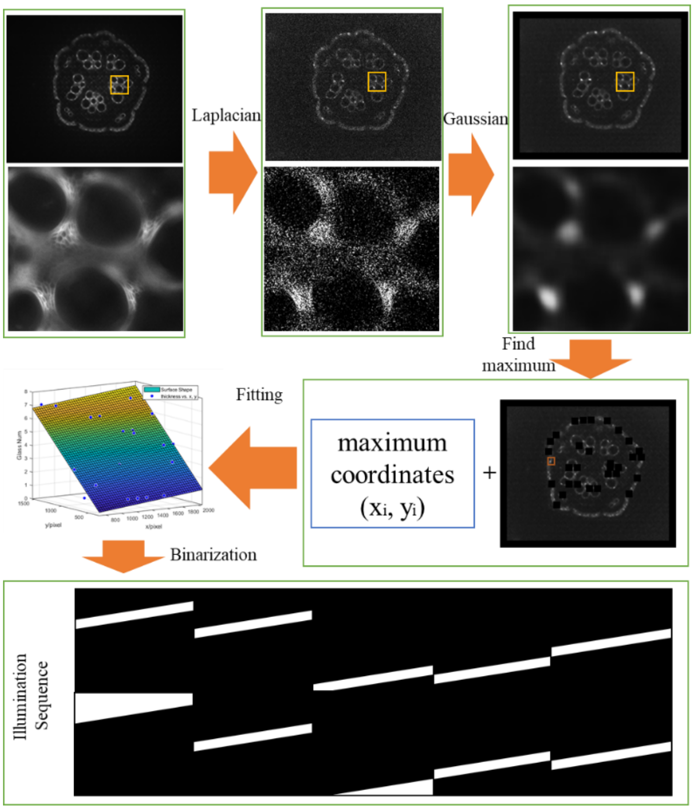

Supplementary Note Fig. 4.3 | Pipeline of the surface detection algorithm.

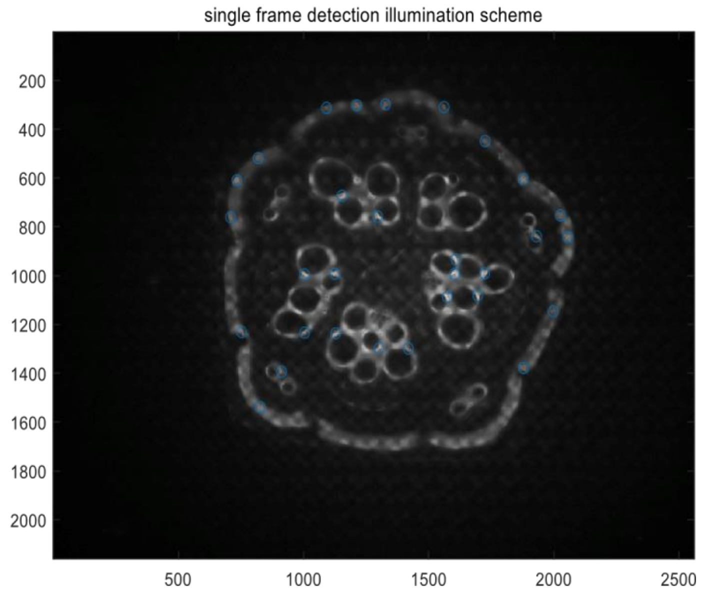

Supplementary Note Fig. 4.4 | Significant patches in surface detection. The blue circles represent the detected in-focus patches in this image. The surface can then be fitted based on the position of these patches. The units for the x- and y-axes are pixels.

4.1.4. Results

The illumination sequence can be transferred to the DMD memory and initiated at the start of the subsequent frame. This enables the entire specimen to come into focus, as depicted in **Supplementary Note Fig. 4.5.**

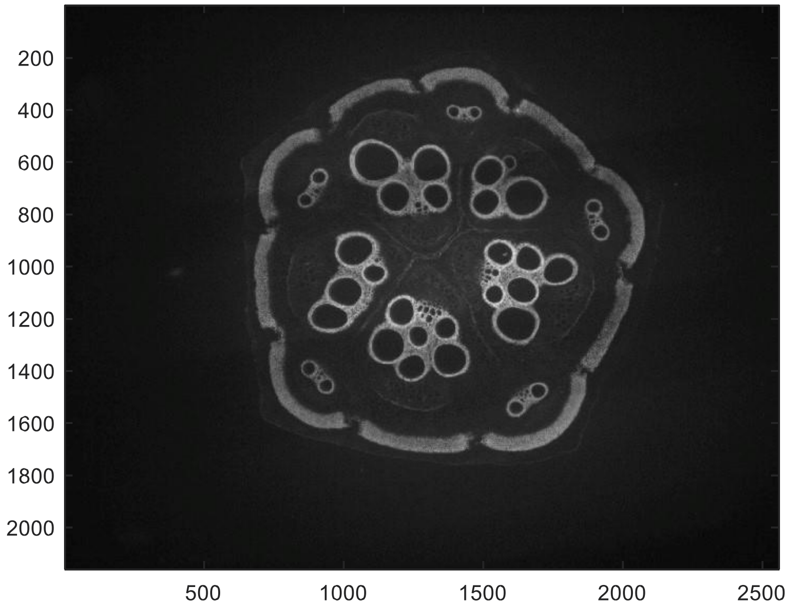

Supplementary Note Fig. 4.5 | The whole image is in-focus when MFIAS is on. Units for the x- and y-axes, pixels.

4.2 Acquisition speed

In the main context, the acquisition speed is set up to 10 fps for the sample properties. Here, we show two examples of fast acquisitions.

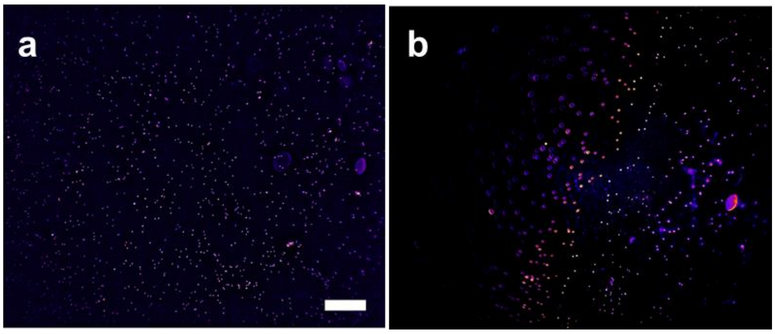

Supplementary Note Fig. 4.6 | Fluorescent beads observed through MFIAS and conventional microscopy at 24 fps. Microspheres with a diameter of 0.5 μm were suspended in phosphate-buffered saline and deposited onto a tilted coverslip. The movement of the beads was recorded through high-speed acquisition in **a** as MFIAS on and in **b** as MFIAS off. Scale bar: 1 mm.

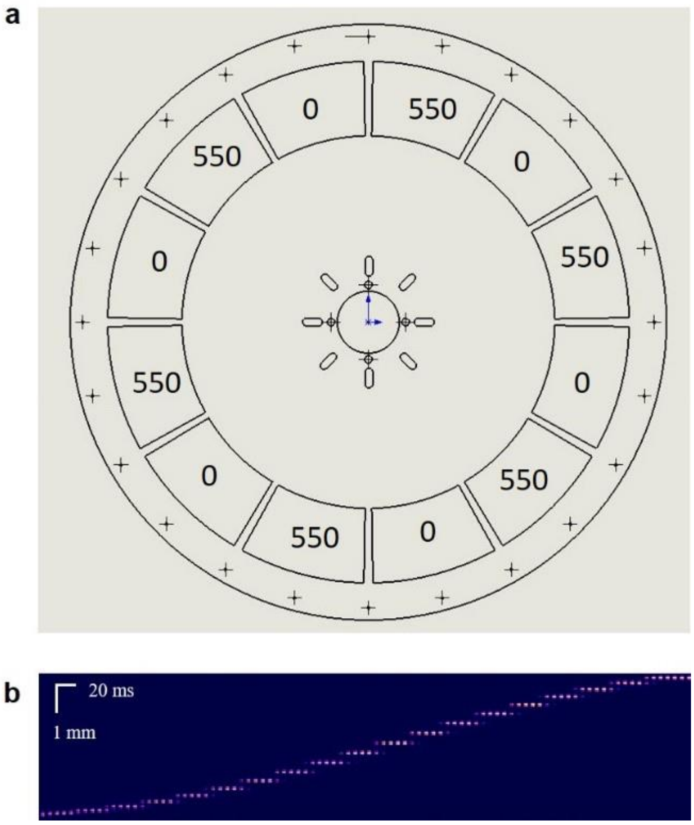

Supplementary Note Fig. 4.7 | High-speed scan of a tilted LCD screen. The design consisted of a disk with 6 thick cover glasses (550 μm) and 6 empty slots, which allowed for the focusing of two planes of 183 μm in a single snapshot. The camera was cropped to a 2560*300 area and recorded at 140 frames per second to capture the dynamics. **a**, A draft of the spinning disk is shown. **b**, An image sequence from 0 ms to 600 ms is presented, with an interval of 7 ms.

Supplementary Note 5 | A Protocol for building MFIAS.

5.1 List of materials

Spinning Disk

- Disk, Customized from CNC
- Glass, Thickness 0.16, 0.33, 0.55, 0.71, 0.88, 0.98, 1.33, 1.43, 1.5 mm
- IR detector
- Rotation stage, IM6824H Lika Tech
- Motor drive, 12-36 V Lika Tech,
- UV glue, 3218uv Valigoo
- Tapes

Selective illumination

- Digital Micromirror Device, V-7001 Vialux
- LED, SOLIS-470C & DC20 Thorlabs
- Relay lenses, AC508-75-A Thorlabs

Synchronization

- DAQ, USB-6363 NI
- Jump wire
- PC

Macroscope

- SLR Lenses, Canon EF 50 mm f/1.4 USM & MINILTA AF 100 mm f/2.8
- Dichroic Mirror, DMLP505L Thorlabs
- Excitation Filter, FESH0500, Thorlabs
- Emission Filter, MF525-39, Thorlabs
- sCMOS Detector, Zyla 5.5 Andor

5.2 Implementation of MFIAS

Step 1: Assembly of the Spinning Disk.

The spinning disk was constructed using alumina, which was oxidized and painted black for optimal performance. A stick was attached to the disk's edge for synchronization. The manufacturer provided a fanshaped cover glass with specific dimensions (outer radius: 110 mm, inner radius: 50 mm, angle: 34 degrees). The glass plates were securely fixed to the disk using UV glue and tape, ensuring that the center of mass remained on the axis. The disk was connected to a rotation stage and motor driver, while an IR detector was positioned close to the disk to detect the stick's position for triggering.

Step 2: Assembly of the illumination.

The illumination setup followed the standard structured illumination light path, with regional illumination design for lower optical requirements. The illumination LED was directed toward the digital micromirror device (DMD) to generate a uniform pattern. The DMD output passed through a doublet lens and objective lens to reach the specimen. To calibrate the system, a fluorescent solution was placed on a slide covered with a cover glass. Various patterns were generated on the DMD, and the resulting images were recorded by the camera. The camera and DMD coordinates were aligned using MATLAB's control point selection tool.

Step 3: Signal Synchronization.

Two modes of synchronization were implemented: master mode and slave mode. In the master mode, the spinning disk's position served as the trigger source, dictating the timing for the DMD and camera. The motor drive voltage controlled the disk's speed. At the beginning of each cycle, the stick on the disk triggered the IR detector, sending a message to the DAQ counter. This triggered the camera's exposure and initiated the DMD display. The camera's exposure time matched the disk's rotation duration, and the DMD's display time matched the time for each cover glass. If multiple cover glasses were present in the field of view, the LED was turned off. In the slave mode, the same process was followed, but a PID process was used to set the motor control voltage, ensuring that the disk's rotation period matched the camera's exposure time.

Step 4: Surface Detection.

A preliminary scan was conducted with the disk rotating at a slow pace, capturing one image per cover glass using the camera. The images were rearranged based on the order of glass thickness. Algorithms were then applied for feature identification. For instance, to locate neurons, the time sequence was subjected to

standard deviation analysis, followed by thresholding to create a binary image. Morphological algorithms were utilized to identify connected regions in the image, which were then sorted based on parameters such as area and eccentricity. The surface shape was estimated assuming a continuous 3D structure and could be modelled using a polynomial or other smooth surface representation. The surface was divided into 10 levels, and corresponding binary masks were created for each level.

Step 5: Image Acquisition.
We installed the following software before image acquisition:
- NI LabVIEW 2019 64bit.
- NI DAQmx.
- NI Vision Acquisition.
- Andor LabView SDK3. We use version 3.15.30000.0, which no longer exists on the website.
- DMD driver ALP 4.3. We used ALP43_install-R706.exe, which no longer exists on the website. We do not guarantee compatibility with the newer version ALP43_install-R765.exe.

To use API in LabVIEW, we first imported the library by 'Tools->Import->Shared library(.dll)' after installing the driver.

We controlled the speed of the rotary stage using an analog output (Dev1/ao1). We connected the IR detector to a digital input (Dev1/PFI9), which triggered a digital output task that measured the speed of the rotary stage using Dev1/ctr0. Finally, we linked digital output (Dev1/port0/line0:2) to the external trigger of the DMD (line0), camera (line1), and LED (line2). The example used was a LabVIEW program.

1. The 'Free Run' mode was used to test the rotary stage, camera, DMD, and LED.
First, we applied a voltage to the 'rotary voltage', and the stage should start to move. By clicking the button next to the 'rotary voltage', the speeds of 'rps' and 'T(ms)' should be displayed. After that, we clicked the 'DMD' and 'LED' buttons to check if they could be controlled. To test the sCMOS camera, we followed these steps: 'open camera' -> 'apply settings' -> 'live' ->'stop live'. Finally, we used'save images' to capture and save some images. (Note: If the camera temperature is high, turn on 'SensorCooling' in Micro-Manager.)
2. The 'Calibration' mode was used to obtain images for surface detection.
First, we set'signal time' to a number that is slightly smaller than 'T(ms)' and set 'Frame number to 1 for the normal sample or set it to 50 for neural detection. After 'apply settings' ->'start cali', images were stored in the folder '.\default_image_path\default_save_path\'.

3. Surface detection, which has been discussed in the Methods.

4. Use 'Acquisition' mode to image the surface
We set the 'acq pattern path' to the folder that contained the calculated DMD patterns. We started the acquisition process by clicking 'apply settings'->' start acq'. Images on the surface would be stored in '.\default_image_path\default_save_path\'.

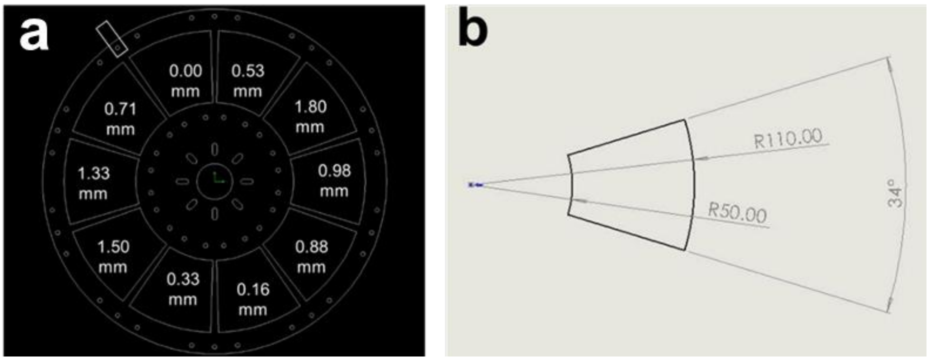

Supplementary Note Fig. 5.1 | Design of the spinning disk.
a, Glass of varying thickness is symmetrically distributed to achieve a balanced moment of inertia.
b, The drawing of the cover glass. Unit, mm.

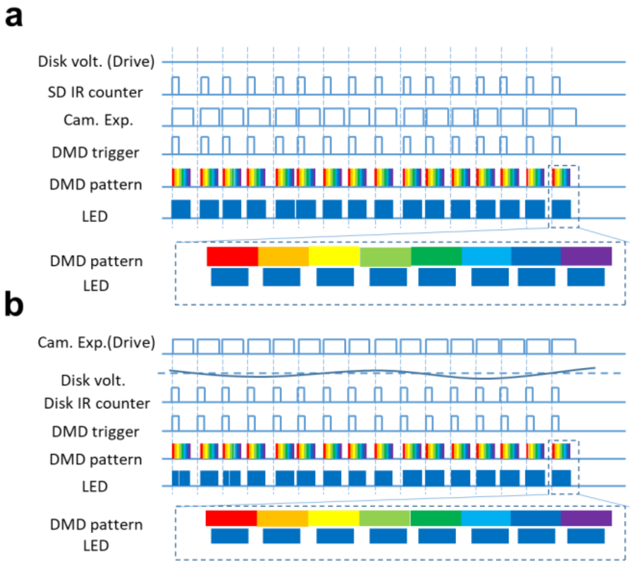

Supplementary Note Fig. 5.2 | Control sequences of the MFIAS system.
a, In the master mode, the spinning disk serves as the trigger for the camera and DMD. **b**, In the slave mode, the camera serves as the trigger for the spinning disk and the DMD. SD, spinning disk. Cam., camera. Volt., voltage. Exp., exposure. IR, infrared light receptor.

Supplementary Note 6 | Replications of biological experiments

6.1. PSNR of neural activities

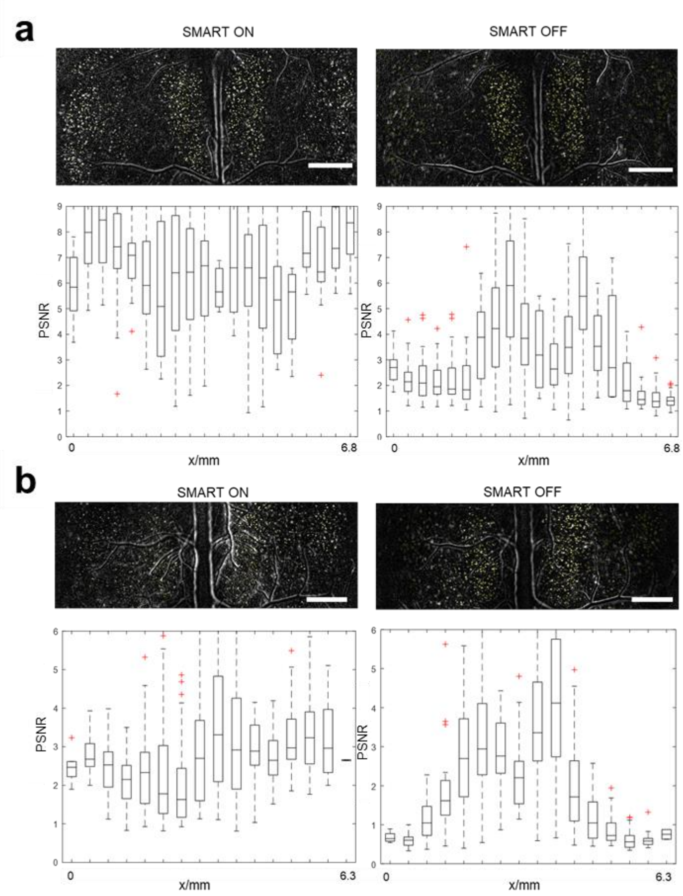

Supplementary Note Fig. 6.1 | Comparison of PSNR distribution for neurons across the field of view between MFIAS and the conventional microscope. a, The result from the biological replicates of 337 neurons from one mouse. **b**, The result from biological replicates of 808 neurons from another mouse. Yellow circles indicate the positions of neurons.
Scale bars, 1 mm. Central black mark: median. Bottom and top edges: 25th and 75th percentiles. Whiskers extend to extreme points excluding outliers (1.5 times above or below the interquartile range). **(a)** and **(b)** are representative micrographs out of 3 biological replicates obtained.

6.2 Visually responsive neurons

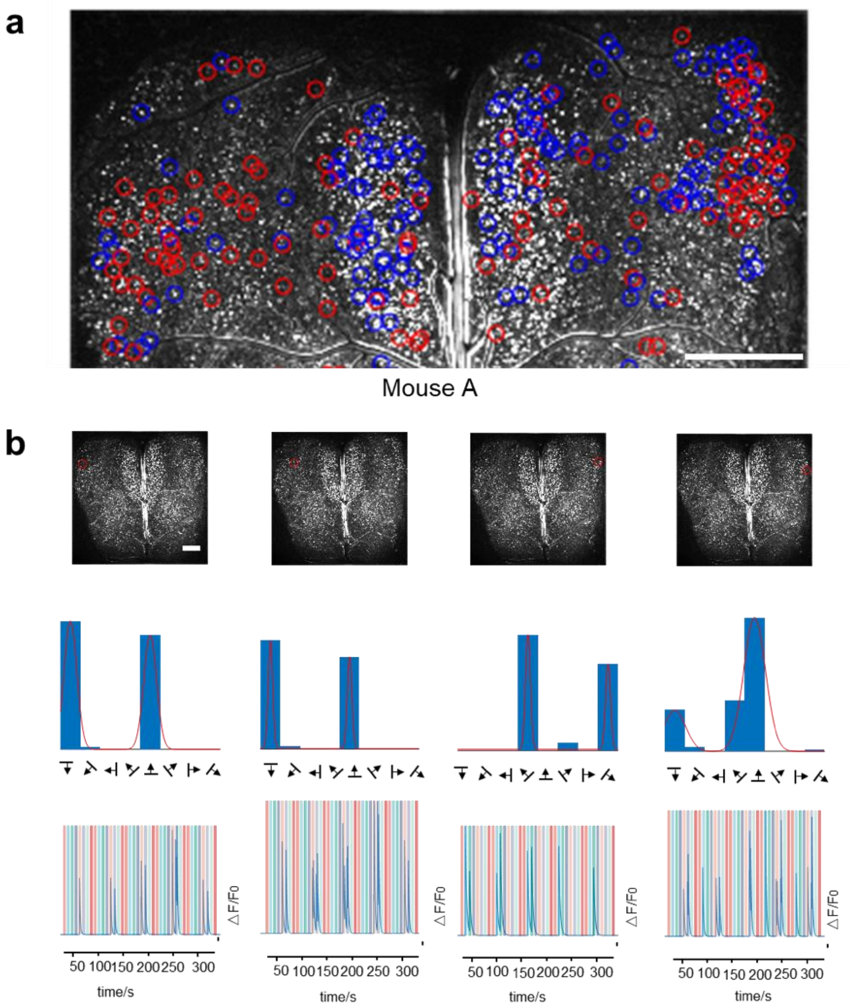

Supplementary Note Fig. 6.2 | Visually responsive neurons in the visual areas of mouse A.
a, Circles indicate visually responsive neurons, and red circles indicate visually responsive neurons with OSI>0.8 (161 out of 330 visually responsive neurons). Scale bar, 1 mm. **b**, Four typical visually responsive neurons from the visual cortex. Top: locations of neurons. Middle: the averaged calcium trace for each orientation and the fitted orientation selectivity curve. Bottom: Calcium traces under the visual stimulus. Scale bar, 1 mm.

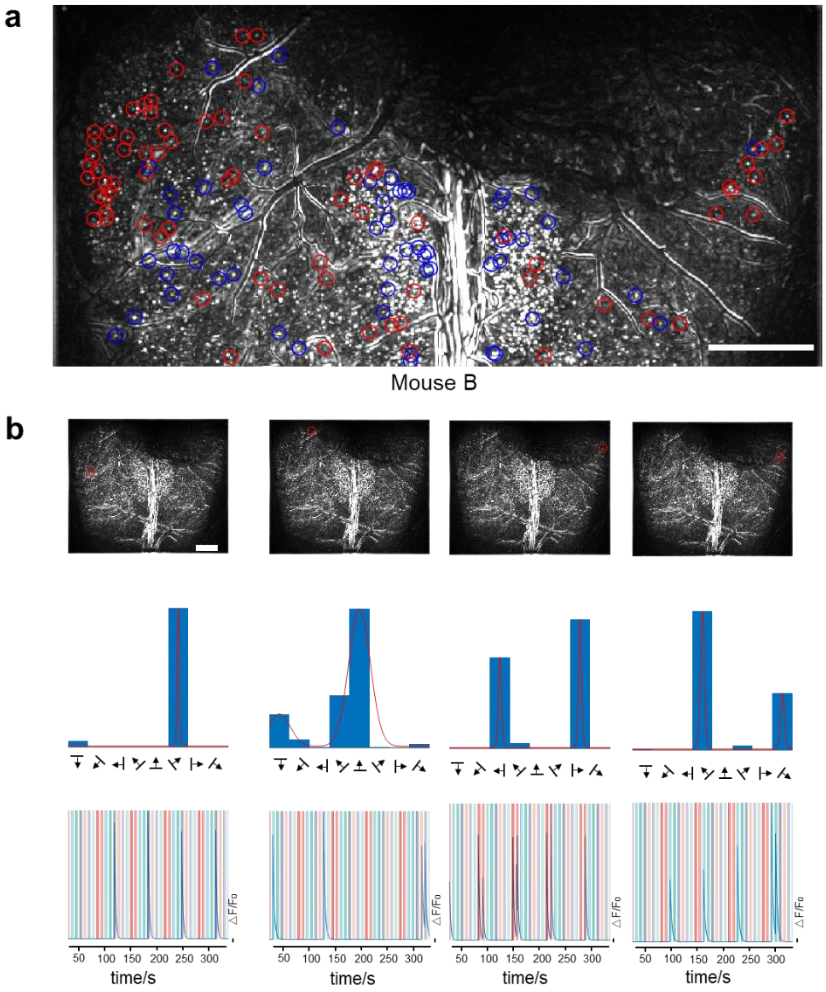

Supplementary Note Fig. 6.3 | Visually responsive neurons in the visual areas of mouse B.
a, Circles indicate visually responsive neurons, and red circles indicate visually responsive neurons with OSI>0.8 (80 out of all 145 visual-responsive neurons). Scale bar, 1 mm. **b**, Four typical visually responsive neurons from the visual cortex. Top: locations of neurons. Middle: the averaged calcium trace for each orientation and the fitted orientation selectivity curve. Bottom: Calcium traces under the visual stimulus. Scale bars, 1 mm.

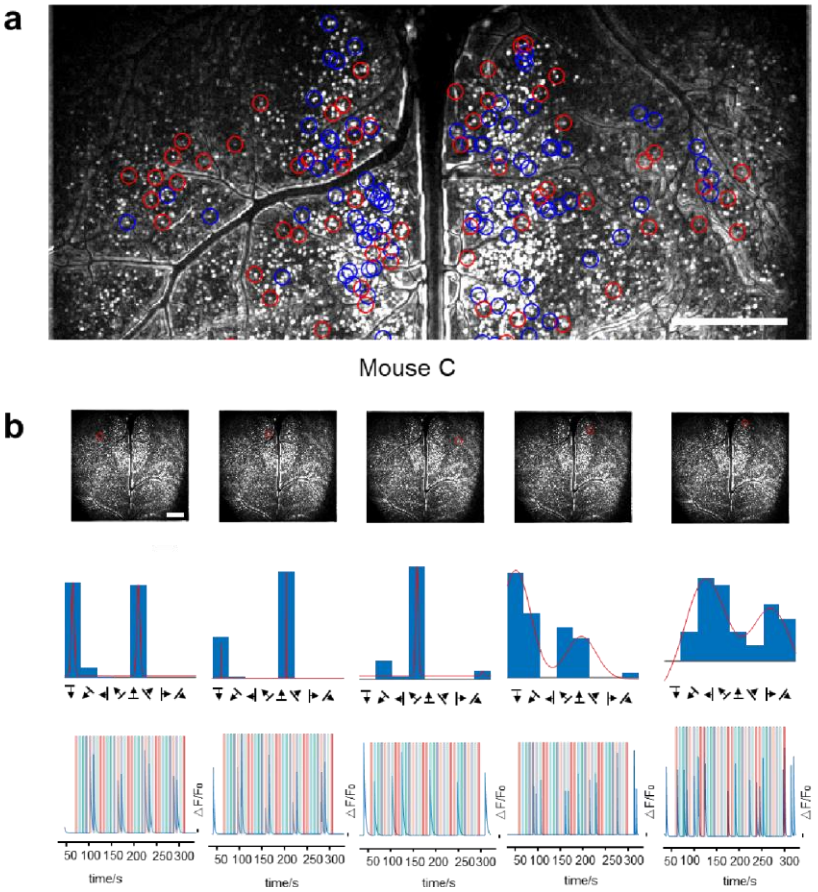

Supplementary Note Fig. 6.4 | Visually responsive neurons in the visual areas of mouse C.
a, Circles indicate visually responsive neurons, and red circles indicate visually responsive neurons with OSI>0.8 (91 out of all 194 visually responsive neurons). Scale bar, 1 mm. **b**, Five typical visually responsive neurons from the visual cortex. Top: locations of neurons. Middle: the averaged calcium trace for each orientation and the fitted orientation selectivity curve. Bottom: Calcium traces under the visual stimulus. Scale bars, 1 mm.

Supplementary Note 7 | Comparison of MFIAS and other SOTA techniques

In this section, we provide a comparative analysis of focal depth extension techniques and present a novel achievement in gigapixel imaging of biological dynamics. Our work demonstrates, for the first time, the ability to capture video-rate gigapixel images with micron resolution on curved surfaces at the centimeter scale. While existing focal adjustment techniques fall short in imaging microscopically curved surfaces, we have overcome this challenge by developing a spinning disk system with multiple cover glasses and a symmetrical planar design. Complementing this hardware innovation, we employed a computational illumination algorithm and a custom high-throughput detector. The key parameters of our MFIAS microscopy approach are summarized in Table 1. The key parameters of our MFIAS microscopy are listed in Table 1:

Table 7.1 | System parameters.

	**MFIAS-RUSH**	**MFIAS-SLR**
Pixel size	0.8 μm	3.5 μm
Maximum FOV	14 mm*12 mm	7 mm*7 mm
Pixel	14000*12000	2560*2160
Max. Frame Rate	10 fps (Experiment)<br>30 fps (Theory, limited by the camera)	24 fps (Experiment, fluorescent beads) 50 fps (theory, limited by the camera’s global shutter mode)<br>140 fps (Reduced FOV and dual planar, LCD screen)<br>>1k fps (dual planar, Estimated by the ultrasonic limit)
Maximum DOF	~400 μm （0.8 strehl ratio）	1 mm （Sample space, 90% enclosed energy in a r=3.2 μm circle）<br>~2 mm (In the image space)<br>8 mm (Theory, in the image space)
Max. Layers. of Depth	10	10 (snapshot)<br>120 (z-scanning)
Num. of Scanning Voxels	16.8 G voxels/s (Experiment)<br>50.4 G voxels/s (Theory)	1 G voxels/s (experiment)
Advantage	High resolution<br>Uniform optical performance<br>Large data throughput	High SNR<br>High excitation and detection efficiency<br>Low cost and high flexibility

Our method outperforms other state-of-the-art 3D imaging techniques in terms of data throughput and planar symmetry. While point scanning microscopes offer high-speed axial scanning, their performance is constrained by the limitations of the resonant galvo or PMT amplifier bandwidth. Light field microscopes, although capable of capturing 3D images in a single shot, suffer from significantly reduced lateral resolution. Focal modulation techniques such as SPED microscopy encounter challenges due to dispersed point spread functions, which impede throughput due to sequential selective planar illumination and recording. COSMOS, on the other hand, employs EMCCD cameras with low intrinsic throughput and compromises lateral resolution by simultaneous recording of two different depths.

Table 7.2 | Comparison of different focal extension techniques.

<table>
<tr><td colspan="3"></td><td></td></tr>
<tr><td rowspan="8">2D detection</td><td rowspan="3">Z-sweeping</td><td>Mechanic z-sweeping of lens or sample</td><td>The trade-off between speed and weight</td></tr>
<tr><td>Electrically tunable lens (ETL, deformable lens)</td><td>1. Trade-off between aperture and speed; 2. Need to be located close to the pupil plane,</td></tr>
<tr><td>Remote Focusing</td><td>Optical relay reduces optical transmission, increases system complexity, extremely difficult for mesoscopic systems like RUSH</td></tr>
<tr><td rowspan="3">3D projection</td><td>Reduce</td><td rowspan="2">Low SNR, need deconvolution because of sidelobes. Lower flexibility, low z-resolution</td></tr>
<tr><td>PSF Engineering (Airy, Bessel)</td></tr>
<tr><td>Wavefront Slitting</td><td>Performance between the above two</td></tr>
<tr><td>Tomography</td><td>Light Feld Microscopy</td><td>Low xy resolution, needs strong before fully recovering the full resolution; Reconstruction artifacts</td></tr>
<tr><td rowspan="5">Point scanning</td><td>z-sweeping</td><td>TAG Lens</td><td rowspan="3">Speed is fundamentally limited by the raster scanning (galvo frequency &PMT amplifier bandwidth)</td></tr>
<tr><td>PSF engineering</td><td>Diffraction-free beam Airy Bessel DOE</td></tr>
<tr><td>Remote focusing</td><td>Remote Focusing ASLM Microscopy</td></tr>
<tr><td rowspan="2">computational</td><td>Z-encoding(vTwINS)</td><td rowspan="2">Need strong prior in reconstruction (like the sparsity prior). Induce artifacts.</td></tr>
<tr><td>Tomography</td></tr>
</table>

Table 7.3 | Comparison of throughput in different microscopes.

Name	FOV	Depth	Pixel Size	Scanned Voxel/second	Reference
Remote Focusing ASLM Microscopy	55μm* 130 μm	130 μm	387 nm	55 M (128*128 pixel *21 slices *156 fps)	Reto Fiolka et al. Light Sci Appl 9, 165 (2020).
Multi-Z Confocal Microscopy	1200 μm* 1200 μm	130 μm	2.6μm	31 M (512*512 pixel* 4 slices *30 fps)	Jerome Mertz at. al. Optica. 2019 Apr 20; 6(4): 389–395.
SID Microscopy.	Φ 900 μm	260 μm	3.2 μm	411 M 2560*2160 pixel *75 fps	Allipasha Vaziri et al., Nat Methods 14, 811–818 (2017).
SPED Microscopy	0.75 mm* 2.99 mm	480 μm	1.6 μm	256 M 2560*400 pixel* 40 slices *6.23 fps	Karl Deisseroth et al., Cell 2015
COSMOS Microscopy	10 mm* 10 mm	D=1300 μm	11μm	69 M 810*1410 pixel* 2 slices *30 fps	Karl Deisseroth et al. Neuron, 2020
MFIAS Microscopy	12 mm* 14 mm	D=450 μm	800 nm	16.8G 14000*12000 pixel*10 slices*10 fps	This work

A comparison with stages and ETLs.

We compared our method with other prevalent techniques, including powerful motorized stages, DSLR lenses, and electrically tunable lenses. Commercial products from companies such as PI, Newport, and Thorlabs were surveyed, and the main parameters are summarized in the table below. Our method exhibits a speed improvement of over 10 times compared to the best powerful stages, DSLR lenses, or electrically tunable lenses. Powerful stages have limitations in terms of load capacity, stroke, and settling time. DSLR lenses, driven by ultrasonic motors, demonstrate performance similar to voice coil motors. Electrically tunable lenses exhibit a tradeoff between aperture and response time.

Table 7.4 | Comparison between MFIAS and stages.

**Type**	**Model**	**Aperture**	**Response & setting time**	**Specifics**
Motorized Stage	PI High-Load Precision Z Stage M202.00	--	70 ms @ 10 kg load & 100 μm axial movement	Experiment test data
Piezo Linear Actuator	New port 8302 Picomotor	--	>500 ms @10 μm step	Inferred from the maximum velocity of 1.2 mm/min
Voice Coil Motor	PIFOC Objective Scanning System ND72Z2LAQ	--	20 ms @ 0.2 kg, 3 μm axial movement	Experimental test data.
Piezo Stage	P-726	--	6 ms @ 10 μm axial movement, without load	Only 100 μm stroke https://www.pi-usa.us/en/products/piezoflexure-nanopositioners/z-nanofocus-piezoscanners-for-microscope-lenses/p-726-pifochigh-load-objective-scanner-200380
Voice Coil Motor	Canon EOS R3	~50 mm	~30 ms	Inferred from the user's manual from https://www.usa.canon.com/support/p/eos-r3
Electrically Tunable Lens (ETL)	Opotune EL-16-40-TC	16 mm	30 ms	https://www.optotune.com/el-16-40-tc-lens
Deformable Lens		10 mm	2 ms (10 mm diameter)	Optics Express Vol. 23, Issue 17, pp. 21931-21941 (2015)<br>Optics Letters Vol. 45, Issue 21, pp. 59015904 (2020)
MFIAS	This work	30-50 mm	4 ms @ 100 μm axial movement (Experiment)<br>0.1 ms* (theory)	*This limitation is inferred from the edge velocity up to the supersonic speed

Supplementary Note 8 | Specific experimental parameters.

Table 8 | Parameters for the experiments.

Experiment	Specimen	Ref./Sample Size	System	Parameters
PSF of MFIAS-RUSH	0.5 µm Fluorescent beads	**Fig. 2b Supplementary Note Fig. 1.5-1.6** n>1,000 beads	MFIAS on RUSH	Stack-by-stack acquisition, one image for each thickness, 140 ms exposure time
PSF of MFIASSLR	0.5 µm Fluorescent beads	**Supplementary Note Fig. 1.7** 900 beads	MFIAS on SLR	Stack-by-stack acquisition, one image for each thickness, 200 ms exposure time
Vasculature dynamics	WT mouse injected with FITC	**Fig. 2c** n=3 mice	MFIAS on SLR	3D surface, 10 fps, 70 ms exposure time
Neural activities	Rasgrf2-2AdCre;Ai48D mouse with skull window	**Fig. 3** n=6 mice for (a)-(c) n=3 mice for (d)-(h)	MFIAS on SLR	3D surface, 10 fps, 70 ms exposure time
		**Extended Data Fig. 2** n=1 mouse	MFIAS on RUSH	3D surface, 10 fps, 70 ms exposure time
	Virus injected mouse	**Supplementary Fig. 3** n=1 mouse	MFIAS on RUSH	3D surface, 10 fps, 70 ms exposure time
	Rasgrf2-2AdCre;Ai48D mouse with skull cleaning	**Supplementary Fig. 4** n=1 mouse	MFIAS on SLR	3D surface, 10 fps, 70 ms exposure time
Immune cells	WT mouse injected with Ly-6G	**Fig. 3** n=4 mice	MFIAS on SLR	3D surface, 5 fps, 140 ms exposure time
	Cx3cr1-GFP mouse	**Extended Data Fig. 3** n=1 mouse	MFIAS on SLR	3D surface, 5 fps, 140 ms exposure time
Solution in a Scindapsus Aureus leaf	Scindapsus Aureus leaf in FITC solution	**Supplementary Fig. 9** n=1	MFIAS on SLR	3D surface, 10 fps, 70 ms exposure time
Reflective modality	Inset specimens	**Extended Data Fig. 5** n=4	MFIAS on a Reflective SLR	3D surface, 10 fps, 70 ms exposure time
Video-rate capture	0.5-µm Fluorescent beads on a tilted glass	**Supplementary Note Fig. 4.6** n=1	MFIAS on SLR	3D surface, 24 fps, 30 ms exposure time
High-speed capture	LCD screen	**Supplementary Note Fig. 4.7** n=1	MFIAS on SLR	Dual planar, 240 fps
The higher axial resolution, larger DOF	0.5-µm fluorescent microspheres on a mouse brain model	**Supplementary Note Fig. 2.1-2.3** n=1	MFIAS on SLR	Stack-by-stack acquisition, one image for each thickness, 140 ms exposure time
High NA lens applications	0.2-µm Fluorescent beads on a tilted glass	**Supplementary Note Fig. 2.4** n=1	MFIAS on high NA objective	Stack-by-stack acquisition, one image for each thickness, 140 ms exposure time

Supplementary Video 1 | Animation of the working principle. The MFIAS system comprises two components: selective illumination and focal modulation. The selective illumination component incorporates a digital micro-mirror device (DMD) that is conjugated with the image plane and modulates the illumination patterns. The focal modulation component is a spinning disk with varying thicknesses of cover glass, which shifts the focal image to different depths. Before frame acquisition, a depth map is generated and converted into a series of illumination patterns displayed on the DMD. During each exposure period, the spinning disk completes one full rotation while the DMD displays one pattern at each glass thickness. The resulting image is an integration of focal areas from different depths.

Supplementary Video 2 | Cortex-wide vasculature imaging in the mouse brain. Capture of a mouse brain image with FITC injection in vessels. Scale bars, 1 mm.

Supplementary Video 3 | Cortex-wide neural imaging in the mouse brain. Images of a Transgenic Mouse Brain that Expresses GCaMP6f in Neurons. This video presents the comparison of SMART images with conventional macroscopes, and local views from SMART. Scale bars, 1 mm in the global view and 100 μm in local views.

Supplementary Video 4 | Cortex-wide immune imaging in the mouse brain. The Recording of Neutrophil Trafficking in a Mouse Brain after a Craniotomy. Left, the global view. Right, local views. Scale bars, 1 mm in the global view, 100 μm in yellow, purple, green, and blue boxes, and 200 μm in the red box.
