# Supplementary material for: Multifocal fluorescence video-rate imaging of centimetre-wide arbitrarily shaped brain surfaces at micrometric resolution
Source: Nat Biomed Eng. 2023 Dec 6;8(6):740–53. doi: 10.1038/s41551-023-01155-6 (PMC11250366; doi:10.1038/s41551-023-01155-6)
Supplement: Supplementary file 2 — Reporting Summary [file 41551_2023_1155_MOESM2_ESM.pdf]

nature portfolio

Corresponding author(s): Hao Xie and Qionghai Dai

Last updated by author(s): Oct 22, 2023

Reporting Summary

Nature Portfolio wishes to improve the reproducibility of the work that we publish. This form provides structure for consistency and transparency in reporting. For further information on Nature Portfolio policies, see our Editorial Policies and the Editorial Policy Checklist.

Statistics

For all statistical analyses, confirm that the following items are present in the figure legend, table legend, main text, or Methods section.

n/a	Confirmed	
☐	☒	The exact sample size (*n*) for each experimental group/condition, given as a discrete number and unit of measurement
☐	☒	A statement on whether measurements were taken from distinct samples or whether the same sample was measured repeatedly
☐	☒	The statistical test(s) used AND whether they are one- or two-sided<br>*Only common tests should be described solely by name; describe more complex techniques in the Methods section.*
☒	☐	A description of all covariates tested
☒	☐	A description of any assumptions or corrections, such as tests of normality and adjustment for multiple comparisons
☐	☒	A full description of the statistical parameters including central tendency (e.g. means) or other basic estimates (e.g. regression coefficient) AND variation (e.g. standard deviation) or associated estimates of uncertainty (e.g. confidence intervals)
☐	☒	For null hypothesis testing, the test statistic (e.g. *F*, *t*, *r*) with confidence intervals, effect sizes, degrees of freedom and *P* value noted<br>*Give P values as exact values whenever suitable.*
☒	☐	For Bayesian analysis, information on the choice of priors and Markov chain Monte Carlo settings
☒	☐	For hierarchical and complex designs, identification of the appropriate level for tests and full reporting of outcomes
☐	☒	Estimates of effect sizes (e.g. Cohen's *d*, Pearson's *r*), indicating how they were calculated

Our web collection on statistics for biologists contains articles on many of the points above.

Software and code

Policy information about availability of computer code

Data collection	The wide-field imaging data were acquired using Micro-Manager 1.4.23 (Free release), Labview2019 64 bit (National Instruments), Matlab R2021a (Mathworks), QT (QT group). Custom algorithms and software are available at https://github.com/Crazyonxh/SMART-microscopy.
Data analysis	All data analyses were performed in Matlab R2020a (Mathworks), Python 3.8.8, ImageJ 1.53c (NIH).<br>The wide-field neural-signals extraction algorithm was modified based on CNMF-E (https://github.com/zhoupc/CNMF_E, version: 1.1.2).<br>For 1p and 2p comparison, local 1p and 2p signals were extracted and compared using CNMF-E (https://github.com/zhoupc/CNMF_E, version: 1.1.2).<br>Calcium traces were filtered and extracted using the OASIS algorithm implemented in CNMF-E (https://github.com/zhoupc/CNMF_E, version: 1.1.2).<br>Histograms of fluorescent beads were plotted with Seaborn (https://seaborn.pydata.org, version: 0.11.1).<br>Tracks of neutrophils were extracted using Trackmate (https://imagej.net/plugins/trackmate/, version: 6.0.1).<br>Circular graphs of neural correlations were plotted using circularGraph (https://www.mathworks.com/matlabcentral/fileexchange/48576-circulargraph, version: 2.0.0).<br>Allen Mouse Brain Common Coordinate Framework (http://labs.gaidi.ca/mouse-brain-atlas)<br>PSF simulation code was modified based on Debye diffraction code (https://github.com/jdmanton/debye_diffraction_code)<br>Custom algorithms and software are available at https://github.com/Crazyonxh/SMART-microscopy.

For manuscripts utilizing custom algorithms or software that are central to the research but not yet described in published literature, software must be made available to editors and reviewers. We strongly encourage code deposition in a community repository (e.g. GitHub). See the Nature Portfolio guidelines for submitting code & software for further information.

Data

Policy information about availability of data

All manuscripts must include a data availability statement. This statement should provide the following information, where applicable:

- Accession codes, unique identifiers, or web links for publicly available datasets
- A description of any restrictions on data availability
- For clinical datasets or third party data, please ensure that the statement adheres to our policy

The main data supporting the results in this study are available within the paper and its Supplementary Information. Data generated in this study, including source data for the figures, are available from figshare with the following identifiers: source data for the figures, https://doi.org/10.6084/m9.figshare.24431824; beads data, https://doi.org/10.6084/m9.figshare.20103707; neuron data, https://doi.org/10.6084/m9.figshare.20103749; neutrophil data, https://doi.org/10.6084/m9.figshare.20103791; vasculature data, https://doi.org/10.6084/m9.figshare.20103716. The raw and analysed datasets generated during the study are too large to be publicly shared, yet they are available for research purposes from the corresponding authors on reasonable request.

Research involving human participants, their data, or biological material

Policy information about studies with human participants or human data. See also policy information about sex, gender (identity/presentation), and sexual orientation and race, ethnicity and racism.

Reporting on sex and gender	The study did not involve human research participants.
Reporting on race, ethnicity, or other socially relevant groupings	—
Population characteristics	—
Recruitment	—
Ethics oversight	—

Note that full information on the approval of the study protocol must also be provided in the manuscript.

Field-specific reporting

Please select the one below that is the best fit for your research. If you are not sure, read the appropriate sections before making your selection.

☒ Life sciences ☐ Behavioural & social sciences ☐ Ecological, evolutionary & environmental sciences

For a reference copy of the document with all sections, see nature.com/documents/nr-reporting-summary-flat.pdf

Life sciences study design

All studies must disclose on these points even when the disclosure is negative.

Sample size	Sample sizes, in our case the number of mice and recordings, were chosen to ensure that animal-to-animal and recording-to-recording variability was reflected in the captured data. We chose $n \geq 3$ for in vivo neural-imaging results and immune-cell imaging results unless otherwise stated. Only animals were included in the study, for which all animal procedures (as described in Methods) worked successfully to allow for cellular imaging. Provided that animal procedures, surgeries, viral injections and the expression of genetically encoded Ca^{2+} indicators were successful, as verified using a standard mesoscope, we found the imaging results and data quality to be reliably reproducible and consistent, both across imaging sessions with the same animal and across animals. Because the goal of the work was to establish an imaging method rather than to report biological findings, we considered this sample size sufficient to verify the performance of the method.
Data exclusions	No data were excluded from the analyses.
Replication	All attempts at replication were successful, and each main result was at least a duplicate of experiments. The numbers of independent experiments are stated in the figure captions or in the main text.
Randomization	Randomization was not relevant to the study, because there were no experimental groups.
Blinding	Blinding was not relevant to the study, because no group allocation was performed.

Reporting for specific materials, systems and methods

We require information from authors about some types of materials, experimental systems and methods used in many studies. Here, indicate whether each material, system or method listed is relevant to your study. If you are not sure if a list item applies to your research, read the appropriate section before selecting a response.

Materials & experimental systems

n/a	Involved in the study	
☐	☒	Antibodies
☐	☒	Eukaryotic cell lines
☒	☐	Palaeontology and archaeology
☐	☒	Animals and other organisms
☒	☐	Clinical data
☒	☐	Dual use research of concern
☒	☐	Plants

Methods

n/a	Involved in the study	
☒	☐	ChIP-seq
☒	☐	Flow cytometry
☒	☐	MRI-based neuroimaging

Antibodies

Antibodies used: Alexa Fluor 488 anti-mouse Ly-6G Antibody, Cas# 127626, lot. B350441, BioLegend.

Validation: The antibody used was validated by the manufacturer. Species relativity, Mouse. Applications, Flow Cytometry , Immunohistochemistry – Frozen, Spatial biology. (https://www.biolegend.com/en-us/products/alexa-fluor-488-anti-mouse-ly-6g-antibody-7085?GroupID=BLG7232#productCertificate)

Eukaryotic cell lines

Policy information about cell lines and Sex and Gender in Research

Cell line source(s): The NSC-34 cell line used was a gift from Qihui Fan's lab, and had been purchased from Hunan Fenghui Biotechnology Co., Ltd.

Authentication: None of the cell lines used were authenticated.

Mycoplasma contamination: The cell lines were not tested for mycoplasma contamination.

Commonly misidentified lines (See ICLAC register): No commonly misidentified cell lines were used.

Animals and other research organisms

Policy information about studies involving animals; ARRIVE guidelines recommended for reporting animal research, and Sex and Gender in Research

Laboratory animals: C57BL/6 and transgenic mice (Rasgrf2-2A-dCre/Ai148D/Cx3CR1-GFP), male and female, 8–12 weeks, 20–30 g, were obtained from The Jackson Laboratory. Mice were housed in standard cages with a maximum of 5 mice per cage. Cages were housed in an environment with a 12-h/12-h dark/light cycle, an ambient temperature of 72F and an ambient humidity of ~30%. Mice were provided food and water ad libitum.

Wild animals: The study did not involve wild animals.

Reporting on sex: Genders are randomized in the experiments.

Field-collected samples: The study did not involve samples collected from the field.

Ethics oversight: All experimental procedures were approved by the Animal Care and Use Committee of Tsinghua University.

Note that full information on the approval of the study protocol must also be provided in the manuscript.
